# The dynamic chemistry of the boron–nitrogen bond

**DOI:** 10.1039/d5sc07665j

**Published:** 2025-11-27

**Authors:** Federico Frateloreto, Giorgio Capocasa, Martina De Angelis, Greta Sandri, Osvaldo Lanzalunga, Chiara Massera, Stefano Di Stefano

**Affiliations:** a Dipartimento di Chimica, Università di Roma La Sapienza and ISB-CNR Sede Secondaria di Roma – Meccanismi di Reazione P.le A. Moro 5 I-00185 Roma Italy federico.frateloreto@uniroma1.it giorgio.capocasa@uniroma1.it stefano.distefano@uniroma1.it; b Dipartimento di Scienze Chimiche, della Vita e della Sostenibilità Ambientale, Università degli Studi di Parma Parco Area delle Scienze 17/A 43124 Parma Italy chiara.massera@unipr.it

## Abstract

Here we report that fully reversible B←N bond formation/cleavage is a promising tool for the achievement of dynamic libraries (DLs) of rapidly interconverting compounds. The composition of a number of minimal DLs of adducts between phenylboronic acid catechol ester 1 and a series of nitrogen-based aromatic heterocycles (*N*ArHets) is demonstrated to be predictable taking into account the association constants related to the formation processes of the single adducts involved. Furthermore, such composition can be controlled over time by the use of activated carboxylic acids (ACAs). Depending on the amount of added ACA, a B←N based DL can be either overturned in terms of composition, transiently overexpressing an adduct initially under-expressed, or transiently fully disassembled into its building blocks.

## Abstract 2

Here we show that dynamic libraries of B←N adducts with a predictable composition can be easily obtained from the related building blocks and that changes of such composition can be programmed over time by use of an activated carboxylic acid.

## Graphical abstract

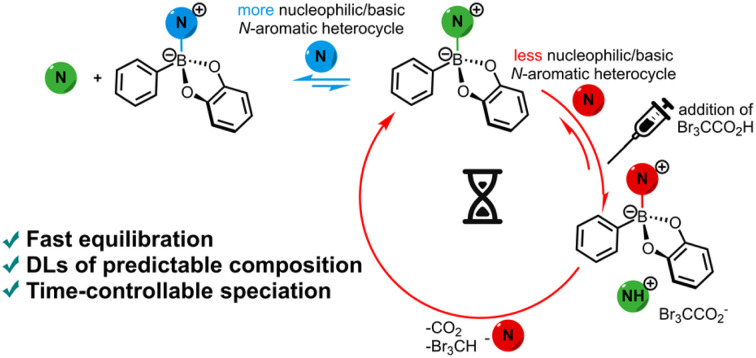

## Introduction

Dynamic combinatorial chemistry (DCC)^1–8^ has been an intense field of investigation for the past two decades due to its implications in different topics such as supramolecular recognition,^9–12^ catalysis,^13–17^ dissipative systems,^18–29^ and systems chemistry.^30–36^ Both covalent and supramolecular reversible bonds have been largely employed to constitute dynamic libraries (with this term, DLs, one typically refers to collections of compounds able to interconvert by exchanging building blocks under equilibrium conditions), with the latter generally having the advantage of very fast kinetics on the human timescale. Nevertheless, the presence of a catalyst may confer the same feature also to covalent dynamic bonds such as imine, acetal, and olefin bonds, among others. Here we report a systematic investigation of the exchange reactions involving the Lewis-pairs formed between phenylboronic acid catechol ester (1) and several nitrogen-based aromatic heterocycles (*N*ArHets), specifically a series of pyridines and *N*-methylimidazole. Such reaction entails the formation/cleavage of the dynamic, covalent (dative) B←N bond. The process turns out to be fast on the human timescale and fully reversible, even in the absence of any catalyst. The B←N exchange process involving catechol boronic esters and *N*ArHets has been already exploited to build a variety of complex molecular structures.^37–40^ Several examples involve the achievement of macrocycles,^41–45^ molecular cages,^46,47^ rotaxanes,^48,49^ supramolecular adducts,^50–55^ covalent organic frameworks (COFs),^56–61^ and polymeric materials.^62–67^

However, despite the episodic use of the B←N bond in the design of (supra)molecular architectures, we feel that a systematic investigation of its properties is still missing. In this work, we aim to build a deeper mechanistic comprehension of the properties of such bonds and to frame the B←N Lewis pair formation within the field of dynamic combinatorial chemistry as a versatile tool for the generation of DLs.

## Experimental section

### Materials

All the *N*ArHets, amines, catechol, phenylboronic acid, and tribromoacetic acid were purchased from Fluorochem, TCI or Merck. Deuterated chloroform was purchased from Fluorochem. Non-deuterated solvents were purchased from Carlo Erba. Deuterated chloroform was passed through short plugs of Na_2_SO_4_ (anhydrous) and Al_2_O_3_ (activated, basic) before use to remove excess water and traces of acids. NMR spectra were recorded on either a BrukerDPX300 or a BrukerDPX400 spectrometer and were internally referenced to the residual proton solvent signal. See SI for further details.

### Synthesis of 1

Catechol (451 mg, 4.1 mmol) was added to a dichloromethane (14 mL) suspension of phenylboronic acid (500 mg, 4.1 mmol). Then, ethyl acetate was added dropwise under stirring until a homogeneous solution was obtained. The resulting mixture was stirred at room temperature overnight. The solution was dried over anhydrous Na_2_SO_4_, filtered, and concentrated under reduced pressure. Recrystallization from hot hexane afforded 1 as clear needle-like crystals (650 mg, 81%). See SI for further information.

## Results and discussion

**Fig. 1:**
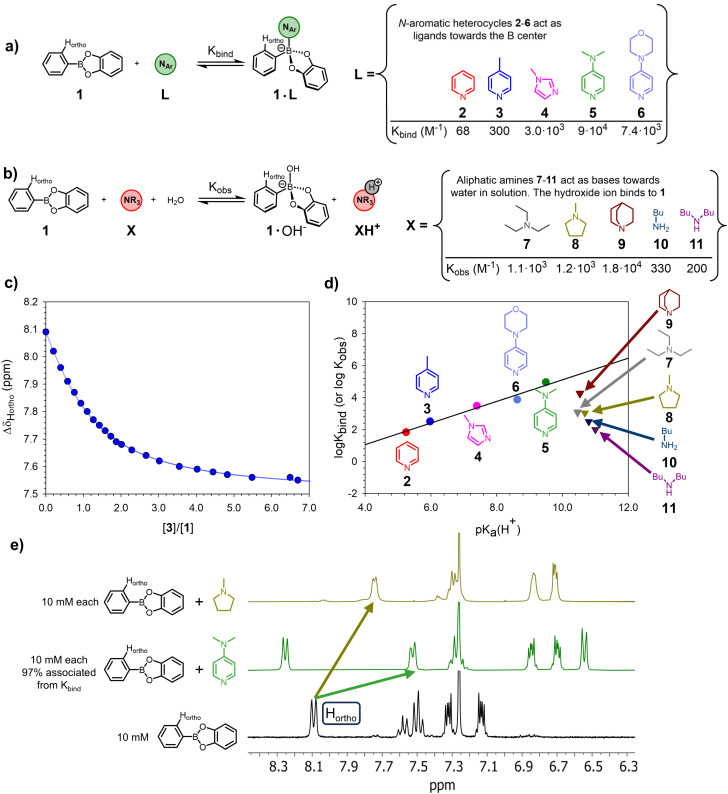
(a) Binding processes between phenylboronic acid catechol ester 1 and *N*ArHets 2–6. (b) Overall proposed transformation occurring in the presence of amines 7–11. (c) ^1^H-NMR titration of 5.0 mM 1 with 3 in CDCl_3_ at 25 °C (see Fig. S23–S32 for the other titrations). (d) Brønsted correlation between log *K*_bind_ (or log *K*_obs_, for aliphatic amines 7–11) and p*K*_a_H^+^ for compounds 2–11 (for pyridines, *ρ* = 0.68, *R*^2^ = 0.97, see Table S2 for details). The linear correlation only holds for *N*ArHets 2–6; the data points relative to amines 7–11 lay outside the line. (e) Comparison of ^1^H-NMR traces of 1 (bottom, black), a mixture of 1 and 5 (centre, green), and a mixture of 1 and 8 (top, gold). Spectra recorded at 25 °C; all compounds are 10 mM in CDCl_3_. Only the aromatics portion is shown. See Fig. S51 for full spectra. The H_*ortho*_ protons on the phenyl moiety on 1 are rendered equivalent in the ^1^H-NMR spectra by the fast rotation about the Ph–B bond.

First, a series of titration experiments was carried out in order to quantify the strength of the interaction between phenylboronic acid catechol ester 1, which can be considered a benchmark boron-based substrate, and a series of *N*ArHets in CDCl_3_ at 25 °C, see Fig. 1a for the relevant equilibria.

The equilibrium constants (*K*_bind_) for the formation of complexes 1·L with L = {2–6} were obtained by plotting the chemical shift of the ^1^H NMR signal of proton H_*ortho*_ on 1 against the *N*ArHets concentration. A 1 : 1 binding model was fitted to the experimental data (see Fig. 1c for the case of the 1·3 adduct and SI for the other cases). For complexes 1·2 and 1·5, the obtained *K*_bind_ values are in good accordance with the values previously measured in the same solvent.^38^

Upon addition of *N*ArHets 2–6 to a solution of 1, the formation of complexes 1·(2–6) occurs immediately and smoothly with no detectable side-product accumulating in solution. These equilibria are fast on the ^1^H NMR timescale, as evidenced by the resolved signals (Fig. 1e, green trace). Furthermore, the higher the p*K*_a_H^+^ of the employed heterocycle, the larger the *K*_bind_ with a very good log *K*_bind_ − p*K*_a_H^+^ Brønsted correlation (Fig. 1d, *ρ* = 0.68, *R*^2^ = 0.97. For any base, p*K*_a_H^+^ is the p*K*_a_ of its conjugate acid.‡‡The log *K*_bind_ values also nicely correlate with Mayr's nucleophilicity parameter; the points related to the log *K*_obs_ of aliphatic amines behave differently (see Fig. S61 and Table S29). All the p*K*_a_H^+^ values are measured in water, see Table S2 and the related caption for details, as well as Fig. S13–S22 for the titration curves and the NMR spectra).

The addition of aliphatic amines 7–11 to a CDCl_3_ solution of 1 also produced a shielding of the H_*ortho*_ proton on 1 with concomitant deshielding of the protons on the amine backbone. Again, a 1 : 1 binding model could be fit to the experimental data. However, even though aliphatic amines 7–11 are more basic than *N*ArHets 2–6, the obtained *K*_obs_ values were significantly lower than the *K*_bind_ values of most *N*ArHets, with no clear dependence on the strength of the base (see Fig. 1d).

Furthermore, in all cases, the ^1^H NMR spectra feature broad signals and spurious peaks not belonging to any readily identifiable species (see Fig. 1e, gold trace).

Additionally, while the saturation value for the H_*ortho*_ chemical shift was the same in all the titrations of 1 with *N*ArHets 2–6 (namely 7.50 ppm, with 4 leading to the only slight outlier of 7.57 ppm), no clear pattern was found for aliphatic amines (all the titration curves are reported in the SI. See Fig. S23–S32). Taken together, this evidence points towards different reactivities of 1 in the presence of *N*ArHets or aliphatic amines.

**Fig. 2:**
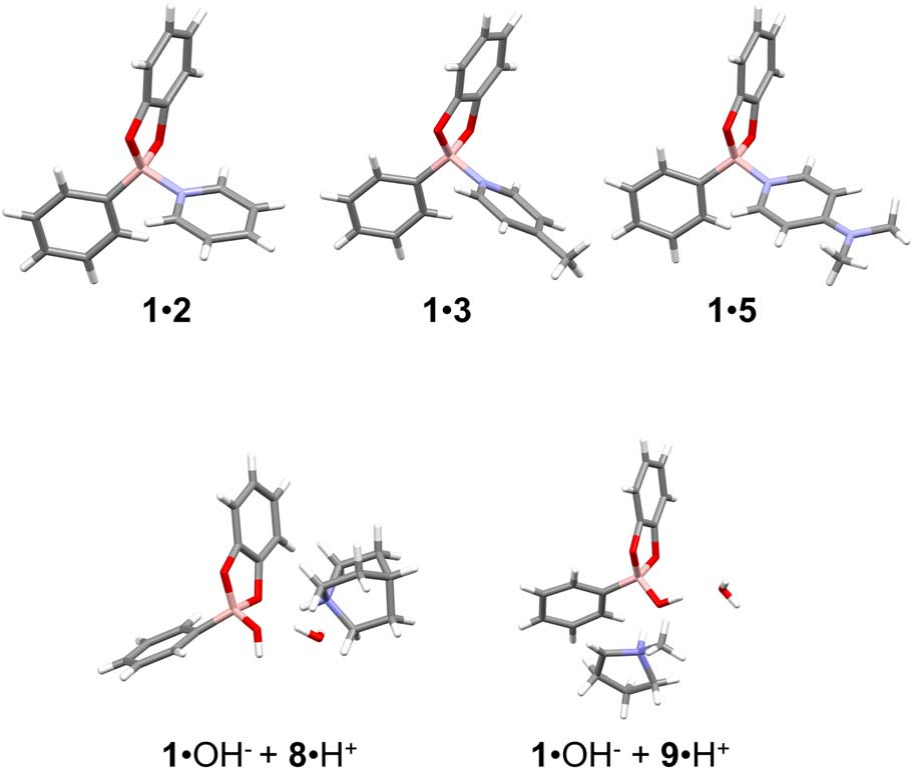
Perspective view of the X-ray molecular structures. Top row: adducts 1·2, 1·3,^70^ and 1·5. Bottom row: co-crystals 1·OH^−^ + 8H^+^ and 1OH^−^ + 9·H^+^ Colour code: B, pink; N, blue; O, red; C, grey; H, white.

Eventually, crystals were obtained from the 1 : 1 mixtures of 1 and amines 8 or 9. The structures feature the binding of a hydroxide ion to the B centre of 1 and the protonated amine engaging in H-bonding with the oxygen of the hydroxide group together with a water molecule (see Fig. 2, Tables S23 and S25). Although these are solid-state structures, we propose that the increased steric bulk around the nitrogen atom and higher basicity of aliphatic amines compared to *N*ArHets favours the deprotonation of adventitious water in the chloroform and the subsequent coordination of the hydroxide ion to the boron (compare Fig. 1a and b), consistently with a reactivity model proposed by Anslyn.^68^ This view is further supported by the fact that sterically bulky lutidine does not appreciably bind to 1 despite its increased basicity with respect to 3 (see Fig. S34 and caption). Similarly, bulky phenylboronic acid pinacol ester is hardly reactive towards 5, the strongest binder for 1 employed in this work (Fig. S36).

Since the formation of 1·HO^−^ will occur to some extent every time that wet chloroform is employed, we tested whether its presence in large quantities would impact the formation of a B←N complex. Therefore, to a 1 : 1 mixture of 1 and 7 (both 12.5 mM), an equimolar amount of 5 was added. The ^1^H NMR spectrum of the resulting mixture was very similar to that of the 1 : 1 mixture of 1 and 5, suggesting that, when possible, the formation of a B←N complex is favoured with respect to that of 1·OH^−^. A similar result, albeit less marked, was obtained by adding 4 in place of 5 (see Fig. S51 and S52).

Having shown that the presence of 1·OH^−^ is not detrimental to the formation of a B←N complex (or, more precisely, that 1·OH^−^ and 1·L with L = *N*ArHet can co-exist as components of a DL, with 1·L being the predominant species), we returned to characterising the B←N adducts.

All the adducts dissociate upon dilution and mass spectroscopy measurements are particularly difficult on the neutral compounds 1·L (L = 2–6) yielding no reliable information. Instead, for the definite identification of such adducts we resorted to X-ray crystallography which also provided us with valuable structural information. For compounds 1·2 and 1·5 it was possible to isolate single crystals suitable for X-ray analysis (Fig. 2) by double layer crystallisation (chloroform/hexane) from 1 to 1 solution mixtures of the two components.

In the case of adduct 1·3, the crystals obtained were not suitable for diffraction analysis. However, the structure of this adduct has been previously solved and deposited in the Cambridge Structural Database (CSD)^69,70^ and has been used as a reference in the present discussion. The crystallographic data and experimental details for data collection and structure refinement of 1·2 and 1·5 are reported in Table S23. The two adducts crystallise with more than one molecule in the asymmetric unit and their geometrical parameters are listed in Table S24. The B–N distances are all quite similar, ranging from 1.634(13) to 1.664(3) Å, with the exception of a slight outlier [1.607(7) Å] displayed by one of the three molecules in adduct 1·5. These values are in good agreement with the average distance of 1.645 Å obtained comparing 126 structures found in the CSD, all containing the B–N_pyridine_ fragment (see Tables S24 and S26).

Unfortunately, all the attempts to obtain single crystals of 1·4 and 1·6 failed. Nevertheless, on account of the similarities in the NMR spectra with the confirmed adducts, we propose that the prevalent process is that of B←N bond formation, rather than water deprotonation.

In order to gain further insights into the nature of the B←N bond occurring between 1 and the *N*ArHets, a computational study was conducted. Calculations were performed with the ORCA 6.1.0 program package.^71,72^ The starting geometries of 1, *N*ArHets, and those of their adducts with 1 were optimised at the r^2^SCAN-3c level of theory, using the SMD solvation model (chloroform).^73,74^ Single point energies and frequences of the optimised structure were then computed at the r^2^SCAN-3c/def2-QZVPP(SMD) level of theory, following a benchmark study focused on Lewis acid–base interaction.^75^

**Fig. 3:**
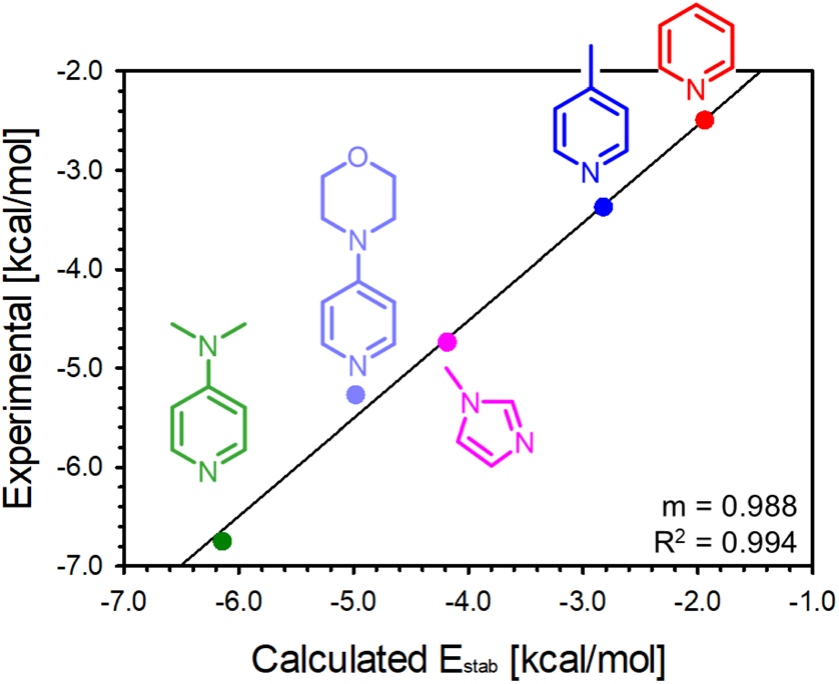
Linear correlation between the experimental stabilisation energies derived from *K*_bind_ measurements, and the computed stabilisation energies for the 1-*N*ArHet adducts.

The stabilisation energies (*E*_stab_) of the interaction between 1 and *N*ArHets were calculated as the difference between the energy of the adduct (*E*_adduct_) and the sum of the energies of the donor *N*ArHet (*E*_d_), and the acceptor 1 (*E*_a_), *i.e. E*_stab_ = *E*_adduct_ − (*E*_d_ + *E*_a_).^76^*E*_stab_ values were then compared with those derivable from the experimentally measured binding constants. Fig. 3 shows a good linear correlation between experimental and theoretical data, with a systematic discrepancy, on average, of about 0.57 kcal mol^−1^ (see Table S27). Moreover, the calculated B–N distances are comparable with those obtained from X-ray crystallography (see Table S28). To rationalise the similar H_*ortho*_ chemical shift saturation values observed for the 1-*N*ArHets adducts, Hirshfeld partial charges were computed (see Table S28). The averaged H_*ortho*_ partial charges for each 1-*N*ArHets range from 0.0313 for 1·5, to 0.0329 for 1·2 with the average value between all the adducts being 0.0321. These values are all very similar, but they are markedly different than the one obtained for free 1, which is 0.0479. The computed partial charges fit well with lower, very similar values of the H_*ortho*_ chemical shift for all the adducts with respect to 1.

Having gained some chemical understanding of the dynamic B←N bond, we turned to building and controlling dynamic libraries of increasing complexity by exploiting its properties.

**Fig. 4:**
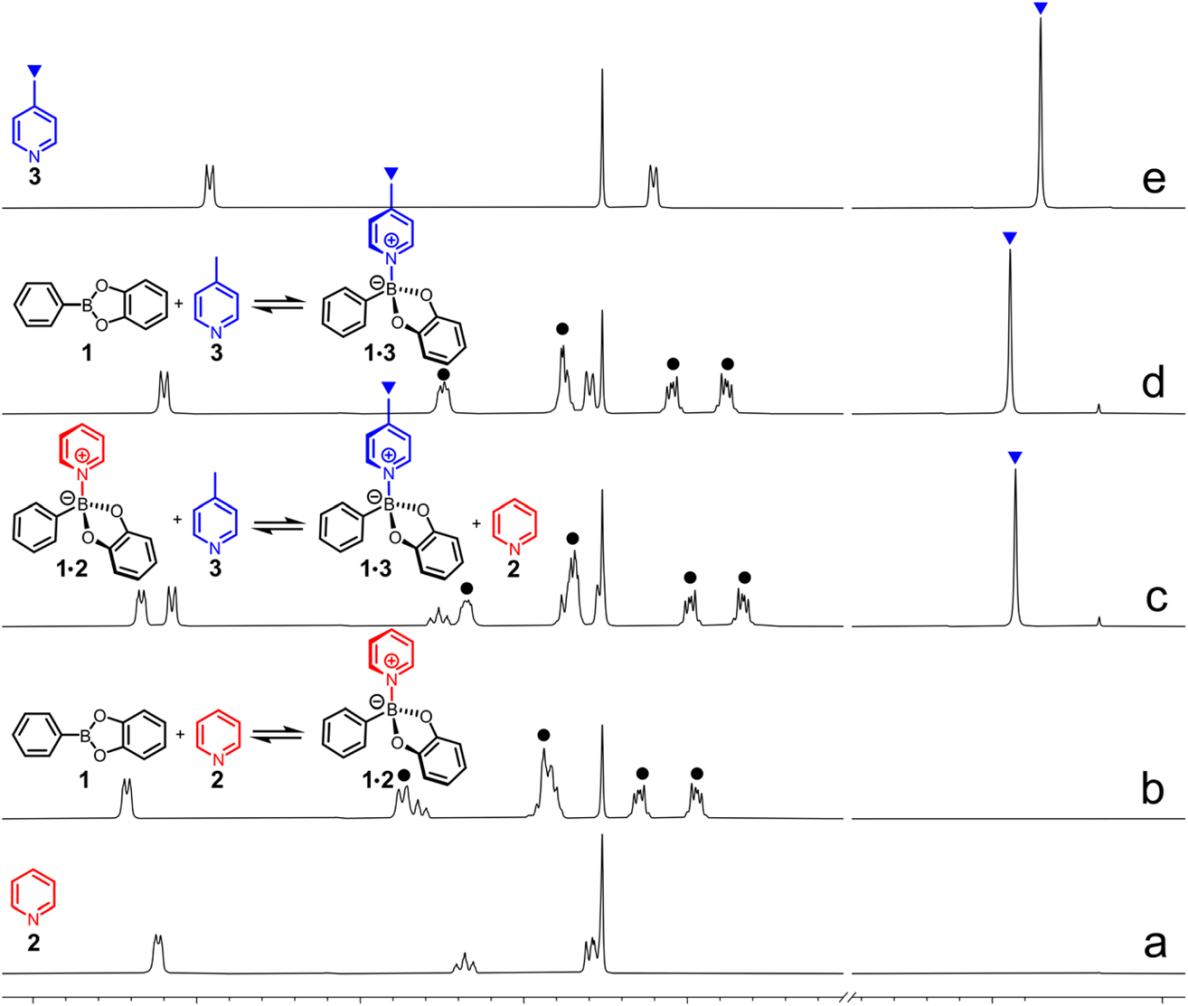
An exchange experiment in which pyridine 2 (red) and 4-methyl pyridine 3 (blue) compete for phenylboronic acid catechol ester 1 (CDCl_3_, 25 °C). All the equilibria are fast on the ^1^H-NMR time scale with adduct 1·3 prevailing on adduct 1·2 under competitive conditions (trace c). Trace a 12.5 mM 2, trace b 12.5 mM 1 and 2, trace c 12.5 mM 1, 2 and 3, trace d 12.5 mM 1 and 3, trace e 12.5 mM 3. The black circles are relative to the signals on the 1 backbone.

Therefore, six competitive experiments, each one involving two couples of adducts 1·L (with L = *N*ArHets 2–5) were carried out in CDCl_3_ at 25 °C as depicted in Fig. 4, where the equilibrium for the couples 1·2 and 1·3 is shown. In all cases, compound 1 and the two given *N*ArHets were added in equimolar amount (12.5 mM), and it was found that the exchange reaction was fast on the ^1^H NMR time scale, with the equilibrium reached immediately after the addition of the reagents (first spectrum recorded). *K*_eq_ for each exchange reaction can be calculated from the ratio between the corresponding *K*_bind_ for each adduct. For example, in the case of the equilibrium 1·2 + 3 ⇄ 1·3 +2 which corresponds to trace c in Fig. 3, *K*_eq_ = *K*_bind_ (1·3)/*K*_bind_ (1·2) = 4.4 with adduct 1·3 abundantly prevailing over 1·2 (free 1 30% of 1_tot_, 1·3 50% of 1_tot_ and 1·2 20% of 1_tot_, see Fig. S45 and the related caption in the SI for details on the mixture composition obtained both experimentally and theoretically). As expected, since in the case of both adducts 1·2 and 1·3 the adopted conditions do not allow for a complete binding, the presence of two molar equivalents of bases (2 + 3 with respect to 1) in the competitive experiment causes a further up-field shift of all signals related to the boronic ester (identified by a black circle in Fig. 4). As for the remaining five DLs the speciation was calculated and the theoretical amount of free 1 was satisfactorily compared to that calculated from NMR data (see Tables S7–S9). Being the strongest binder for ester 1, 4-dimethylaminopyridine 5 shifts the equilibrium toward adduct 1·5 whenever it is involved (see Fig. S47, S49, and S50). These experiments clearly demonstrate the ease of achieving DLs of B←N adducts able to rapidly interconvert on the human timescale, with a predictable composition.

**Fig. 5:**
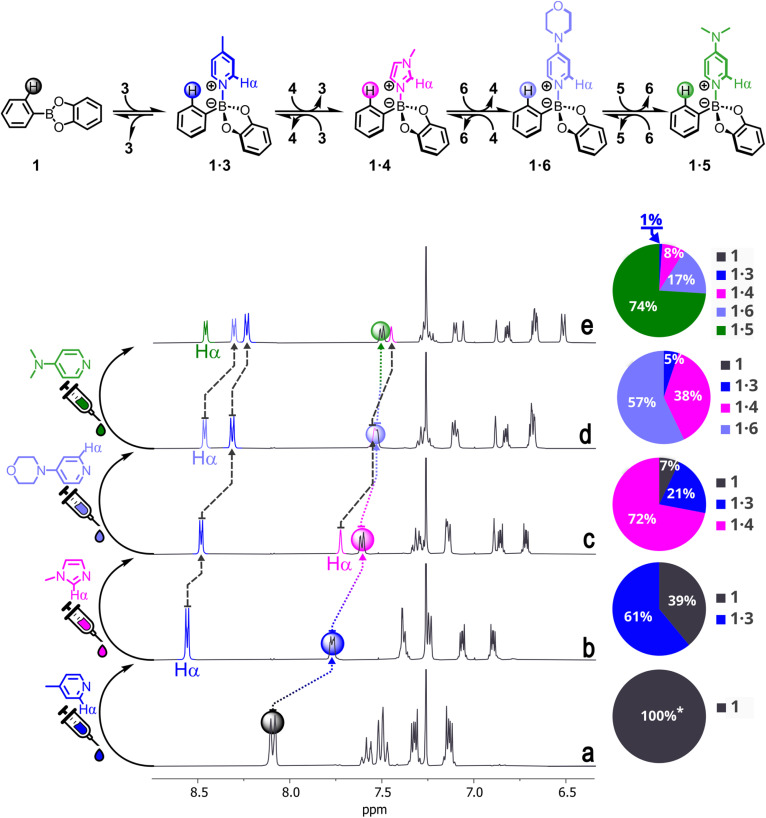
Top: Simplified reaction scheme for the consecutive generation of DLs containing compounds 1 (black), 3 (blue), 4 (pink), 6 (light purple), and 5 (green, in order of addition) together with complexes 1·3, 1·4, 1·6, and 1·5 (in order of increasing stability). Bottom: NMR spectra of 1 (trace a); a mixture of 1 and 3 (trace b); a mixture of 1, 3, and 4 (trace c); a mixture of 1, 3, 4, and 6 (trace d); a mixture of 1, 3, 4, 6, and 5 (trace e). In each spectrum, the signal relative to each Hα proton is highlighted in the same colour as the *N*ArHet (see structures). The H_*ortho*_ proton is marked with a circle in the colour corresponding to the most prevalent adduct. To the right of each spectrum, a pie chart reports the calculated speciation of each DL. See Tables S17 and S18 for the ChemEQL-assisted speciation of the mixtures. All compounds are added 12.5 mM in CDCl_3_, the spectra are recorded at 25 °C. Only the aromatics region is shown. See Fig. S53 for the full spectra. * = in the absence of any ligands, 1 is in equilibrium with a small amount (<5% in our conditions) of phenylboronic acid and catechol due to partial hydrolysis from traces of water in the solvent. The addition of *N*ArHets suppresses this side-reaction.

Next, we performed consecutive competition experiments, building DLs with an increased number of components (Fig. 5 shows the reaction scheme, the NMR spectra, and the computed speciation for each DL). Thus, 1.0 mol equiv. of 3 was added to a 12.5 mM CDCl_3_ solution of 1 (1.0 mol equiv., black trace). Complex 1·3 partially formed and, as expected, the signal relative to the H_*ortho*_ proton on 1 was shifted upfield, while those belonging to 3 moved downfield (blue trace). Addition of 1.0 mol equiv. of 4 to this mixture partially displaced 3, as evidenced by the upfield shift of the protons on the latter (pink trace). The H_*ortho*_ signal also moved upfield, indicating increased binding of 1, which was distributed between its free form and the bound ones (1·3 and 1·4). Then, 1.0 mol equiv. of 6 was added to the solution, causing a further upfield shift of the signals belonging to 3 and 4, as well as of the H_*ortho*_ proton (light purple trace). Finally, 1.0 mol equiv. of 5 were added to the mixture. This resulted in an upfield shift of the signals belonging to all the previously added ligands and brought the H_*ortho*_ proton to its saturation value of 7.50 ppm, indicating complete binding.

Thus, at the end of the experiment, the DL contained compounds 1, 3, 4, 6, and 5 (in order of addition) together with complexes 1·3, 1·4, 1·6, and 1·5 (in order of increasing stability). After each addition, the computed amount of free 1 was satisfactorily compared to that estimated from NMR data (see Fig. S53, Tables S17 and S18).

**Fig. 6:**
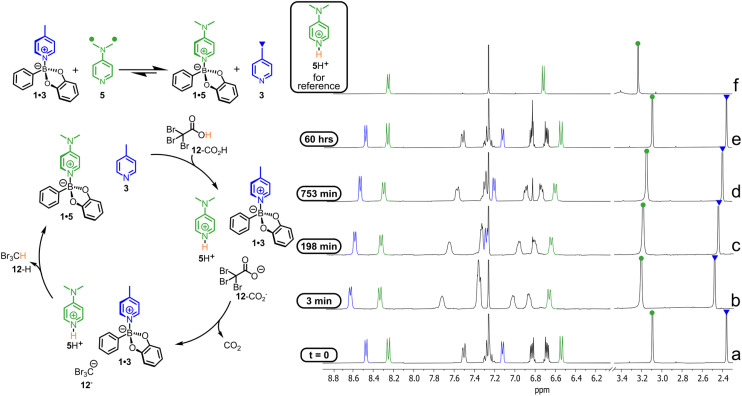
A minimal DL composed of 1·5, 1·3, and related free building blocks driven by ACA 12-CO_2_H (see text). All compounds (1, 3, 5 and 12-CO_2_H) are added in solution at 15.0 mM concentration (CDCl_3_, 25 °C). On each spectrum, the relevant signals for 3 are marked in blue, while those of 5 are coloured in green. Spectra (a–e) are recorded over the course of the reaction. Spectrum (f) shows the signals of 5H^+^, obtained by adding in solution 5 and trifluoroacetic acid at 15.0 mM concentration.

Next, we investigated the chance to control over time a minimal DL composed of two B–N adducts and related *N*ArHets using an activated carboxylic acid (ACA) as a stimulus. ACAs are used for the operation of dissipative chemical systems, which possess one or more Brønsted basic sites.^77–89^ For instance, the DL 1·3 + 5 ⇄ 1·5 + 3 was rapidly obtained by adding in CDCl_3_ 15.0 mM equimolar 1, 3 and 5 (Fig. 6, ^1^H NMR, trace a). As expected from the *K*_bind_ values reported in Fig. 1a, adduct 1·5 largely prevails on 1·3. Addition of 15.0 mM ACA 12-CO_2_H (tribromoacetic acid) causes the extensive protonation of 5, the strongest base present in solution, which is subtracted from the equilibrium. The latter is now shifted to the left with 1·3 prevailing on 1·5.

Comparison between traces a and b of Fig. 6 shows that all signals are shifted down-field soon after the addition of 12-CO_2_H (trace b). Such shifts are due to protonation of 5 to 5H^+^, whose signals are found down-field shifted with respect to the corresponding ones in adduct 1·5 and to partial association of 3 to 1, which causes the down-field shift of the signals related to both moieties 3 and 1.

However, the new state (Fig. 6, trace b) is not an equilibrium one since the ACA conjugate base 12-CO_2_^−^ slowly decarboxylates and the corresponding, just formed carbanion 12^−^ retakes the proton from 5H^+^ to give free pyridine 5 and bromoform 12 (traces b to e).

Consequently, the initial equilibrium with 1·5 prevailing on 1·3 is restored (trace e). Thus, when the decarboxylation of ACA 12-CO_2_H is complete, the DL goes back to the initial equilibrium state. The above interpretation of the experimental results is strictly consistent with the higher sensitivity of the affinity of the basic nitrogen atoms for the proton than for the boron atom of 1 (for log *K*_bind_*vs.* p*K*_a_H^+^*ρ* = 0.68, see Fig. 1d and S33 and the related caption for more details). Furthermore, a computer assisted calculation of the DL speciation based on the p*K*_a_H^+^ of 3 and 5 and p*K*_bind_ of 1·3 and 1·5 confirms such reading (see Tables S21 and S22). However, another corroborating experiment that further shows the fidelity of an ACA driven DL based on the B←N bond was carried out in the presence of excess acid 12-CO_2_H.

**Fig. 7:**
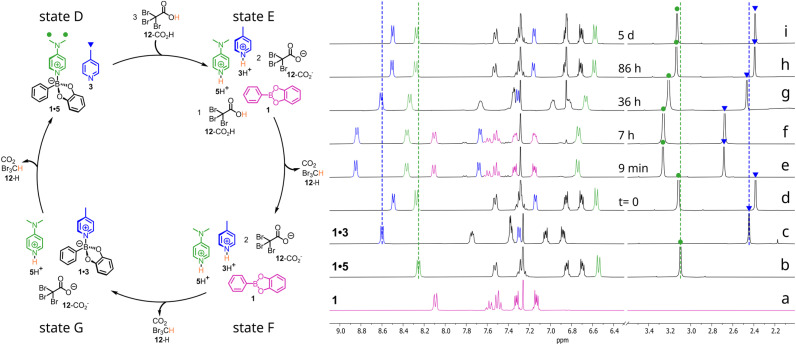
Behaviour of a DL based on the B←N bond under the action of excess ACA 12-CO_2_H. Traces (a) 10 mM 1; (b) adduct 1·5 obtained by mixing 10 mM 1 with 10 mM 5; (c) adduct 1·3 obtained by mixing 10 mM 1 and 10 mM 3; (d) equilibrated solution of 10 mM 1, 3 and 5 before addition of 30 mM ACA 12-CO_2_H (*t* = 0); (e–i) monitoring over time of solution in trace d after addition of ACA 12-CO_2_H. CDCl_3_, 25 °C. Dashed vertical lines are guides for the eye. On each spectrum, the relevant signals for 1 are marked in pink, those of 3 in blue, while those of 5 are coloured in green.

Compounds 1, 3 and 5, 10.0 mM each, were added in CDCl_3_ at 25 °C. The thermodynamic equilibrium with adducts 1·5 prevailing on 1·3 was immediately reached as expected (see state D and trace d in Fig. 7 where the ^1^H NMR spectra of free 1, 1·3 and 1·5 are also reported as traces a, b and c, respectively, for the sake of comparison). Addition of 30.0 mM 12-CO_2_H causes protonation of both pyridines 3 and 5, which are liberated in solution affording free 1 (see trace e in Fig. 7 recorded at *t* = 9 min from addition of the ACA). The new state E, which can be defined as a dissipative state, persists as long as the excess acid is present (traces e and f, recorded at time *t* = 9 min and 7 h, are both consistent with the presence of excess acid). When the excess is over (from state F onward), the less basic 3 is first deprotonated forming adduct 1·3, as shown by trace g (*t* = 36 h), which roughly corresponds to state G of Fig. 7. At this point the strongest base 5 is still mostly protonated and cannot effectively engage in bonding with 1. Then, ACA 12-CO_2_H is further consumed and free base 5 starts to be available for the formation of 1·5 at the expense of 1·3. Eventually, when ACA 12-CO_2_H is exhausted, the system goes back to the initial equilibrium state with 1·5 again prevailing on 1·3 (see trace i in Fig. 7, *t* = 5 days, which corresponds again to state D). A corollary of this experiment is that ACA 12-CO_2_H can also be used to temporally drive the simple binding equilibrium between ester 1 and bases 2–6. And in fact, Fig. S40 shows that addition of ACA 12-CO_2_H to complex 1·5 causes the liberation of 1 in solution due to the transient protonation of 5. When the decarboxylation of 12-CO_2_H is over, adduct 1·5 is reversibly and completely restored. The process turned out to be fully reversible as demonstrated by an experiment in which three subsequent cycles were triggered by three successive additions of 12-CO_2_H. At the end of each cycle (see Fig. S44), adduct 1·5 was found completely reassembled, also proving that bromoform is not detrimental to the B←N bond chemistry to any extent. Thus, similarly to what was observed in the case of the transimination reaction,^27,29,89^ the use of ACA also allows the B←N bond exchange to drive dissipative^27^ dynamic libraries (DDLs) over time in a predictable fashion.^28^

## Conclusions

In this report we show that the B←N bond between phenylboronic acid catechol ester and a series of *N*-based aromatic heterocycles is fully reversible and can be conveniently used to obtain dynamic libraries of interconverting compounds. In chloroform, such B←N exchange reaction turns out to be rapid on the ^1^H NMR time scale and its thermodynamic fate easily predictable from the formation equilibrium constants (*K*_bind_) of the exchanging adducts. Moreover, it is shown that the exchange reactions among B←N adducts as well as their formation can be finely controlled over time in a dissipative fashion using activated carboxylic acids (ACAs). It is expected that such reversible interaction will be exploited in the near future for a number of applications such as (i) the generation of dynamic libraries composed of more complex chemical structures whose composition can be controlled in a dissipative fashion, (ii) the design of boron-based receptors for *N*ArHet anchoring groups with the chance to control the related binding process over time, and (iii) the achievement of stimuli responsive materials and dynamic polymer networks based on the exchange of the *N*-donor species.

## Author contributions

Conceptualization: F. F, G. C, and S. D. S. Data curation: F. F, G. C, M. D. A, and C. M. Formal analysis: G. C, F. F, and C. M. Funding acquisition: S. D. S and C. M. Investigation: all authors. Methodology: F. F, G. C, O. L, and M. D. A. Supervision: F. F, C. M, and S. D. S. Writing: F. F, G. C, M. D. A, C. M, and S. D. S.

## Conflicts of interest

There are no conflicts to declare.

## Supplementary Material

SC-OLF-D5SC07665J-s001

SC-OLF-D5SC07665J-s002

## Data Availability

CCDC 2413767, 2413768 and 2492086 contain the supplementary crystallographic data for this paper.^90*a*–*c*^ The data supporting this article have been included as part of the supplementary information (SI). Supplementary information: details on the synthesis of 1, titration of 1 with *N*ArHets 2–6, crystallographic data, NMR spectra, ORCA input and output files, and speciation of the DDLs obtained. See DOI: https://doi.org/10.1039/d5sc07665j.
