# Supplementary material for: The dynamic chemistry of the boron–nitrogen bond
Source: Chem Sci. 2025 Nov 27;17(5):2582–91. doi: 10.1039/d5sc07665j (PMC12687285; doi:10.1039/d5sc07665j)
Supplement: SC-017-D5SC07665J-s001 [file SC-017-D5SC07665J-s001.pdf]

Supplementary Information (SI) for Chemical Science.
This journal is © The Royal Society of Chemistry 2025

Electronic Supplementary Information (ESI)

The Dynamic Chemistry of the Boron-Nitrogen Bond

Federico Frateloreto,[*,a,§] *Giorgio Capocasa,*[*,a,§] *Martina De Angelis,*[a,§] *Greta Sandri,*[a] *Osvaldo Lanzalunga,*[a] *Chiara Massera*[*,b] *and Stefano Di Stefano*[*,a]

[a] Dipartimento di Chimica, Università di Roma La Sapienza and ISB-CNR Sede Secondaria di Roma - Meccanismi di Reazione, P.le A. Moro 5, I-00185 Roma, Italy, E-mail: federico.frateloreto@uniroma1.it, giorgio.capocasa@uniroma1.it, stefano.distefano@uniroma1.it

[b] Dipartimento di Scienze Chimiche, della Vita e della Sostenibilità Ambientale, Università degli Studi di Parma, Parco Area delle Scienze 17/A, 43124 Parma, Italy, E-mail: chiara.massera@unipr.it

§ These authors contributed equally

Table of Contents

Instruments and Materials......2

Experimental procedures......3

Synthetic procedures......3
Synthesis of phenylboronic acid catechol ester. (1)......3
General procedure for the preparation of phenylboronic acid catechol ester adducts.......3

Spectral data......4

NMR titration experiments......16

Effect of the bulkiness around the B atom......38
Selected example of speciation for the DL obtained from the combination of 1 with bases 2 and 3.......43
Repeatability tests on the time-controlled, 12-CO_2H-enabled dissociation of 1•5......46

NMR competitive experiments......51

Transiently controlling the composition of a DL generated from 1, 3 and 5 with ACA 12-CO_2H......65

X-Ray Diffraction Analysis......70

Crystal data and structure refinement information for adducts 1•2 and 1•5, and for the cocrystal 1•OH^- + 9•H^+......71
Selected geometrical parameters (Å, °) for 1•2 and 1•5......72
Ortep views......73
List of B-N pyridine distances (Å) found in the CSD......75

Computational Study......77

List of optimized geometries in Cartesian coordinates......77
Energy calculation, computed B-N bond distances, and Hirshfeld partial charges......85

Correlation between Mayr's nucleophilicity parameters and logK_{bind} (or log$_{Kobs}$) values......86

References......88

Instruments and Materials

The compounds whose synthesis is not described here were purchased from Fluorochem, TCI or Merck.
Deuterated chloroform was purchased from Fluorochem. All non-deuterated solvents were purchased from Carlo Erba.
Unless otherwise specified, the commercially available reagents and solvents were used as received from the purveyors, with no prior treatment.
Deuterated chloroform was passed through short plugs of Na_2SO_4 (anhydrous) and Al_2O_3 (activated, basic) before use, to remove excess water and traces of acids.
PTFE tape and Parafilm (overlapped) were employed to seal the NMR tubes to prevent solvent evaporation.
Unless otherwise specified, the NMR experiments were performed at T = 25 °C.
NMR spectra were recorded on either a BrukerDPX300 or a BrukerDPX400 spectrometer and were internally referenced to the residual proton solvent signal.

Experimental procedures

Synthetic procedures

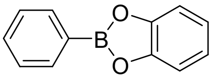

1

Synthesis of phenylboronic acid catechol ester. (1) Catechol was added (451 mg, 4.1 mmol) to a suspension of phenylboronic acid (500 mg, 4.1 mmol) in dichloromethane (14 mL). Then, ethyl acetate was added dropwise until a homogeneous solution was obtained and the resulting mixture was stirred at room temperature overnight. The solution was dried over anhydrous Na_2SO_4, filtered, and concentrated under reduced pressure. Recrystallization from hot hexane afforded **1** as clear crystals (650 mg, 81%). NMR data are in accordance with those reported in literature.[1]

General procedure for the preparation of phenylboronic acid catechol ester adducts. Unless otherwise stated, in an NMR tube, 50.0 µL of a 70.0 mM $CDCl_3$ solution of compound **1** (3.50 µmols) were added to 600 µL of $CDCl_3$. Then, 50 µL of a 70.0 mM solution of compound **Z** (that represent a *N*-based aromatic heterocycle, *N*ArHet, see Scheme S1 below) in $CDCl_3$ were added to the solution. The obtained final concentration was 5.00 mM. Eventually, the ^{1}H NMR spectrum of the adduct was recorded. For spectra at different concentrations of **1** and **Z**, the solutions were prepared and mixed in the appropriate relative amounts. In all cases, the exchange between the adduct and its components at room temperature is fast on the NMR timescale. For each adduct spectrum reported, we used the appropriate pK_{bind} to calculate the extent of the complexation.

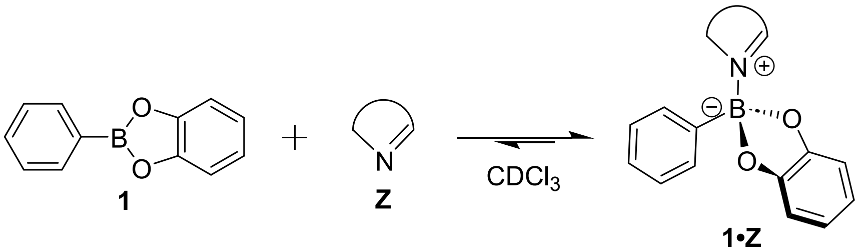

1 Z 1•Z

Scheme S1. Generic scheme for the preparation of phenylboronic acid catechol ester adducts **1•2 - 1•6**

Table S1. Structure and name of adducts **1•2 - 1•6**.

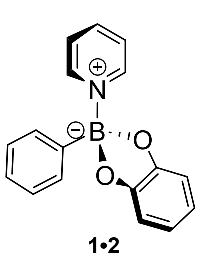 1•2	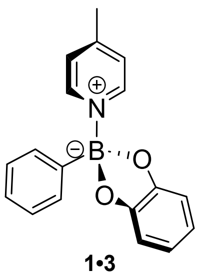 1•3	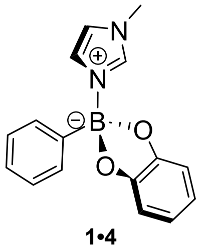 1•4	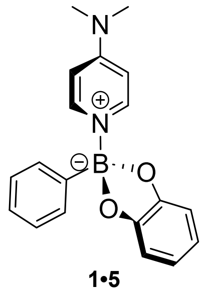 1•5	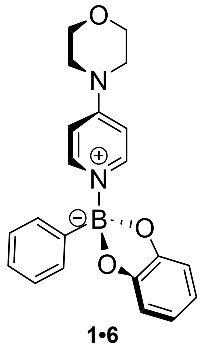 1•6
**Z** = Pyridine (**2**)	**Z** = 1-methylpyridine (**3**)	**Z** = 1-methylimidazole (**4**)	**Z** = 4-dimethylaminopyridine (DMAP, **5**)	**Z** = 4-(pyridin-4-yl)morpholine (**6**)

Spectral data

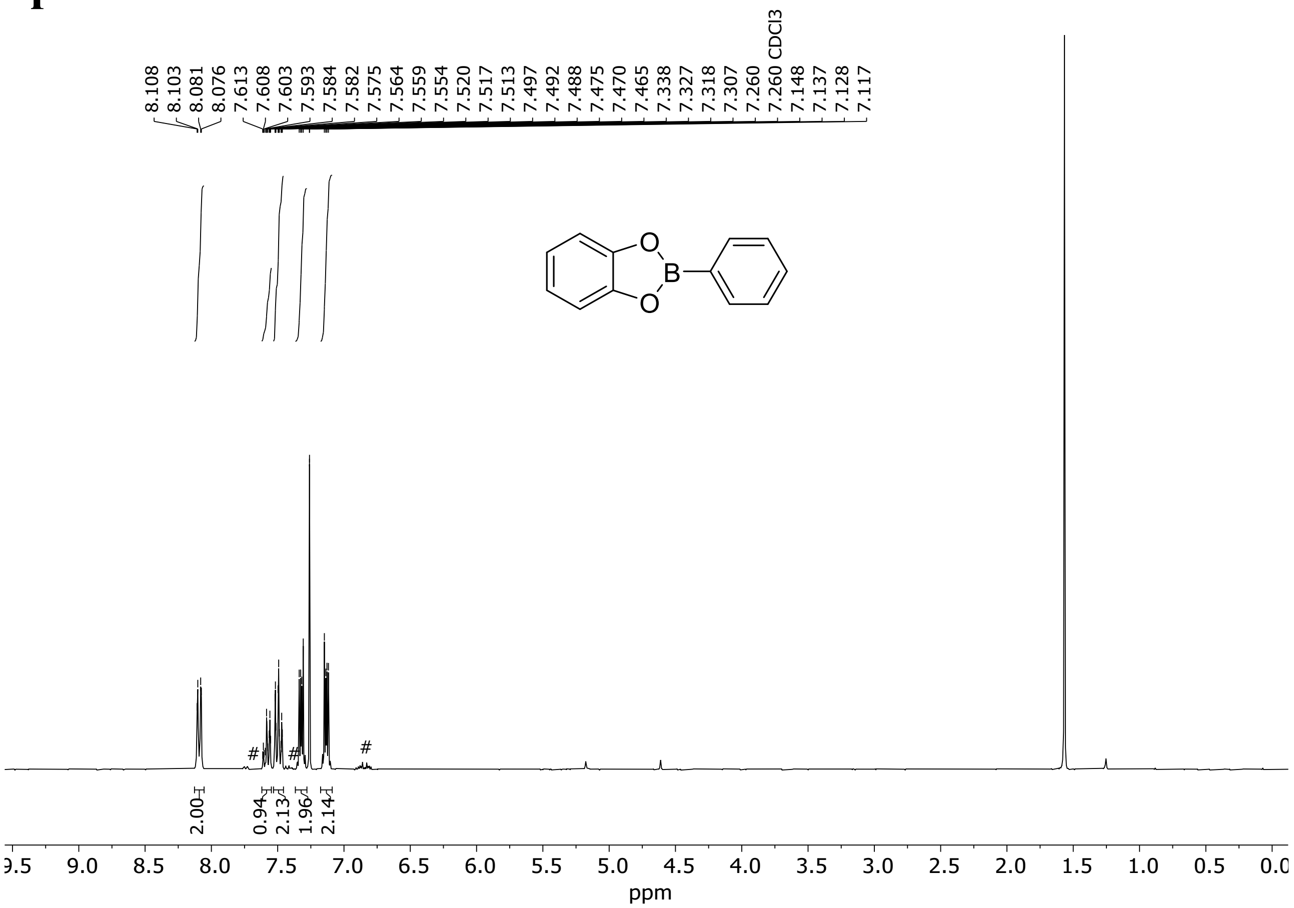

Figure S1. ^{1}H NMR spectrum of **1**. The signals marked with a # sign are due to traces of phenylboronic acid and catechol deriving from the hydrolysis of **1**. Such signals are reduced in intensity upon addition of the nitrogen bases.

^{1}H NMR (300 MHz, $CDCl_3$, 25°C) δ 8.09 (dd, J^1 = 7.5 Hz, J^2 = 1.5 Hz, 2H), 7.58 (tt, J^1 = 2.7 Hz, J^2 = 1.5 Hz, 1H), 7.49 (tt, J^1 = 6.6 Hz, J^2 = 1.5 Hz, 2H), 7.32 (m, 2H), 7.13 (m, 2H) ppm.

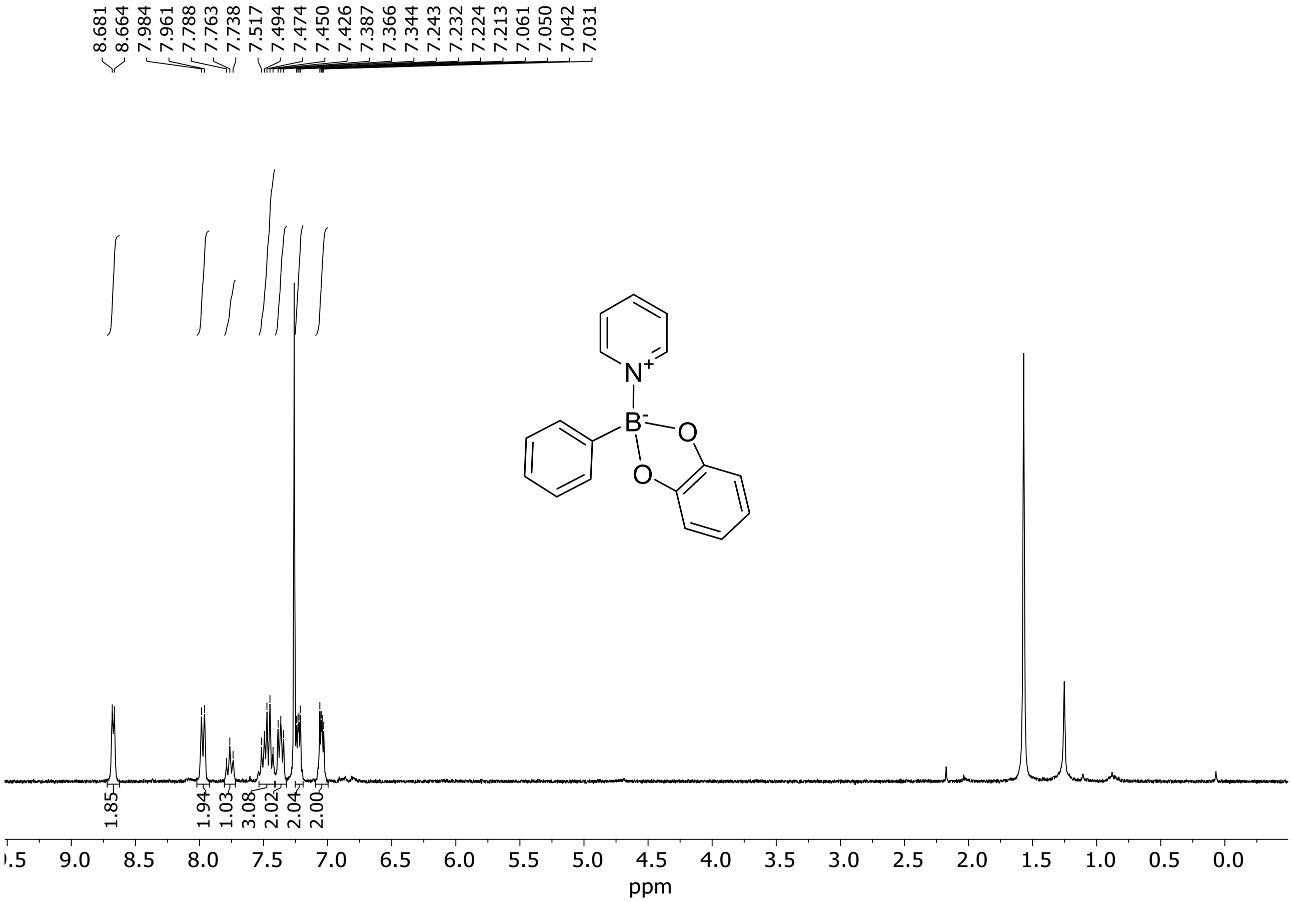

Figure S2. ^{1}H NMR spectrum of a solution containing **1** and **2** (both 5 mM in $CDCl_3$, 25° C. Expected association 21%).

^{1}H NMR (300 MHz, $CDCl_3$, 25°C) δ 8.67 (d, J^1 = 6 Hz, 2H), 7.97 (d, J^1 = 6 Hz, 2H), 7.76 (t, J^1 = 6 Hz, 1H), 7.48 (m, 3H), 7.37 (t, J^1 = 6 Hz, 2H), 7.23 (m, 2H), 7.05 (m, 2H) ppm.

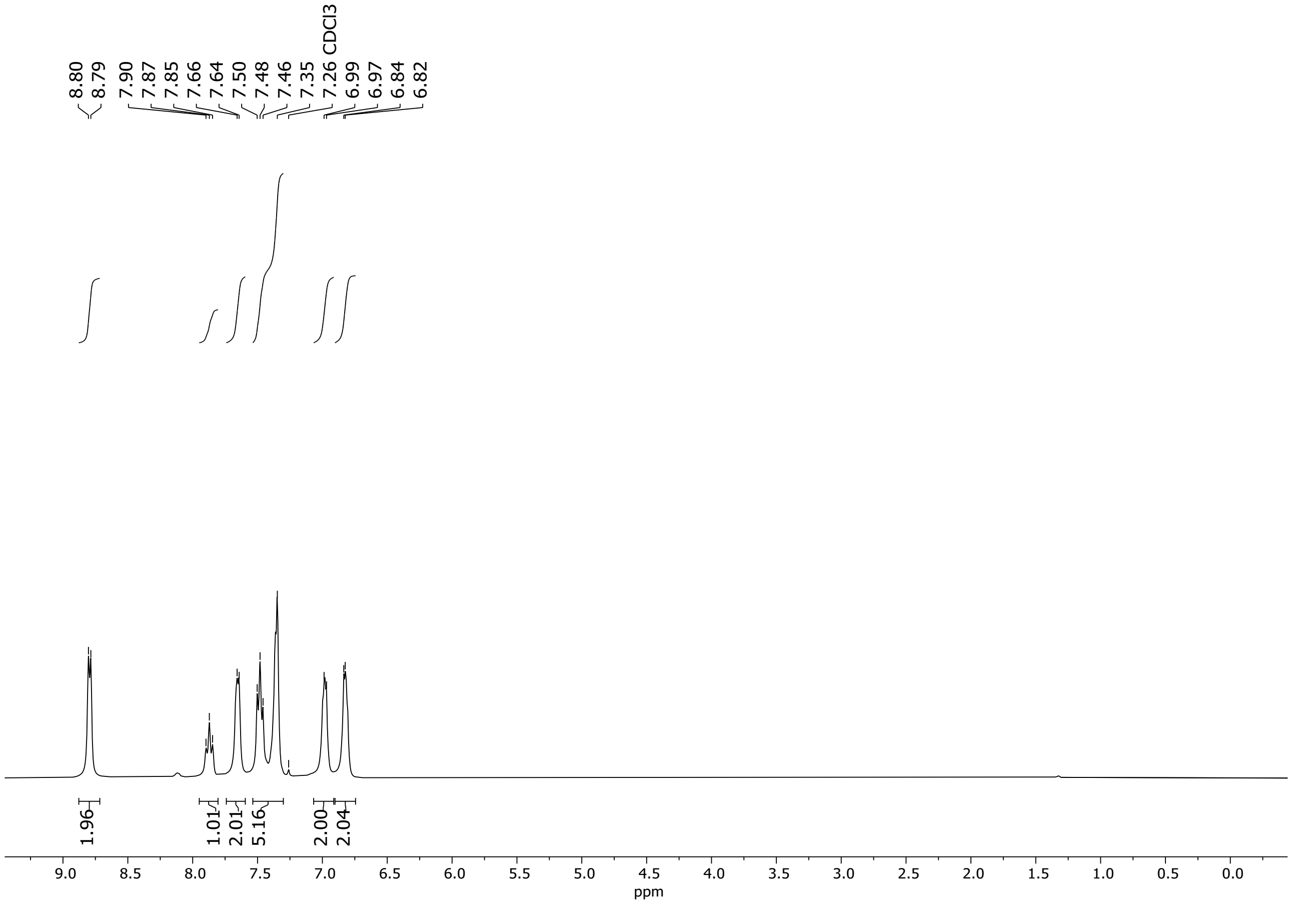

Figure S3. ^{1}H NMR spectrum of a solution containing **1** and **2** (both 0.42 M in $CDCl_3$, 25° C. Expected association 83%).

^{1}H NMR (300 MHz, $CDCl_3$, 25°C) δ 8.80 (d, J^1 = 3 Hz, 2H), 7.87 (t, J^1 = 9 Hz, 1H), 7.65 (d, J^1 = 6 Hz, 3H), 7.41 (m, 5H), 6.98 (m, 2H), 6.82 (m, 2H) ppm.

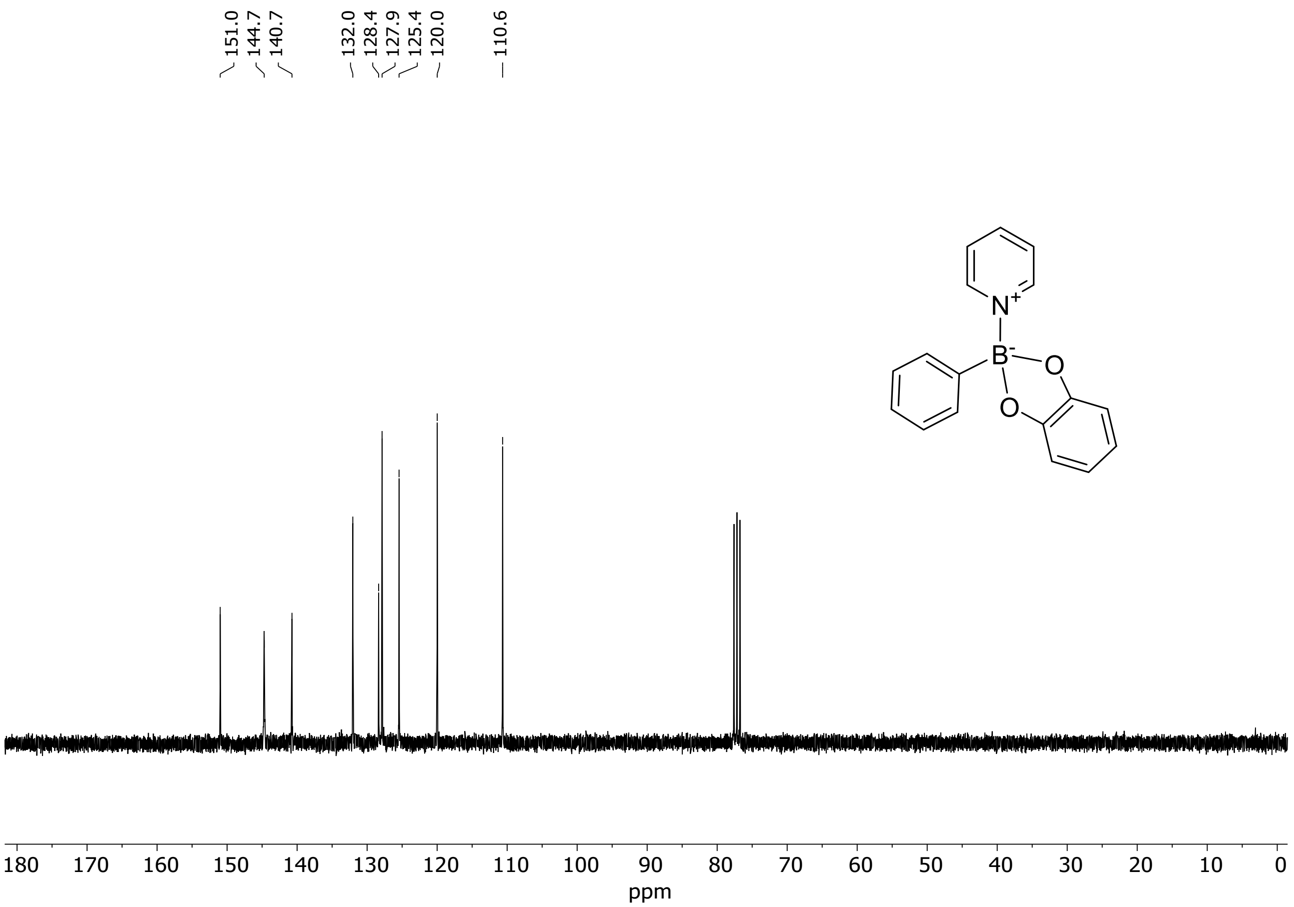

Figure S4. ^{13}C NMR spectrum of a solution containing **1** and **2** (both 0,42 M in $CDCl_3$, 25° C. Expected association 83%).

^{13}C NMR (75 MHz, $CDCl_3$, 25°C) δ 151.0, 144.7, 140.7, 132.0, 128.4, 127.9, 125.4, 120.0, 110.6 ppm.

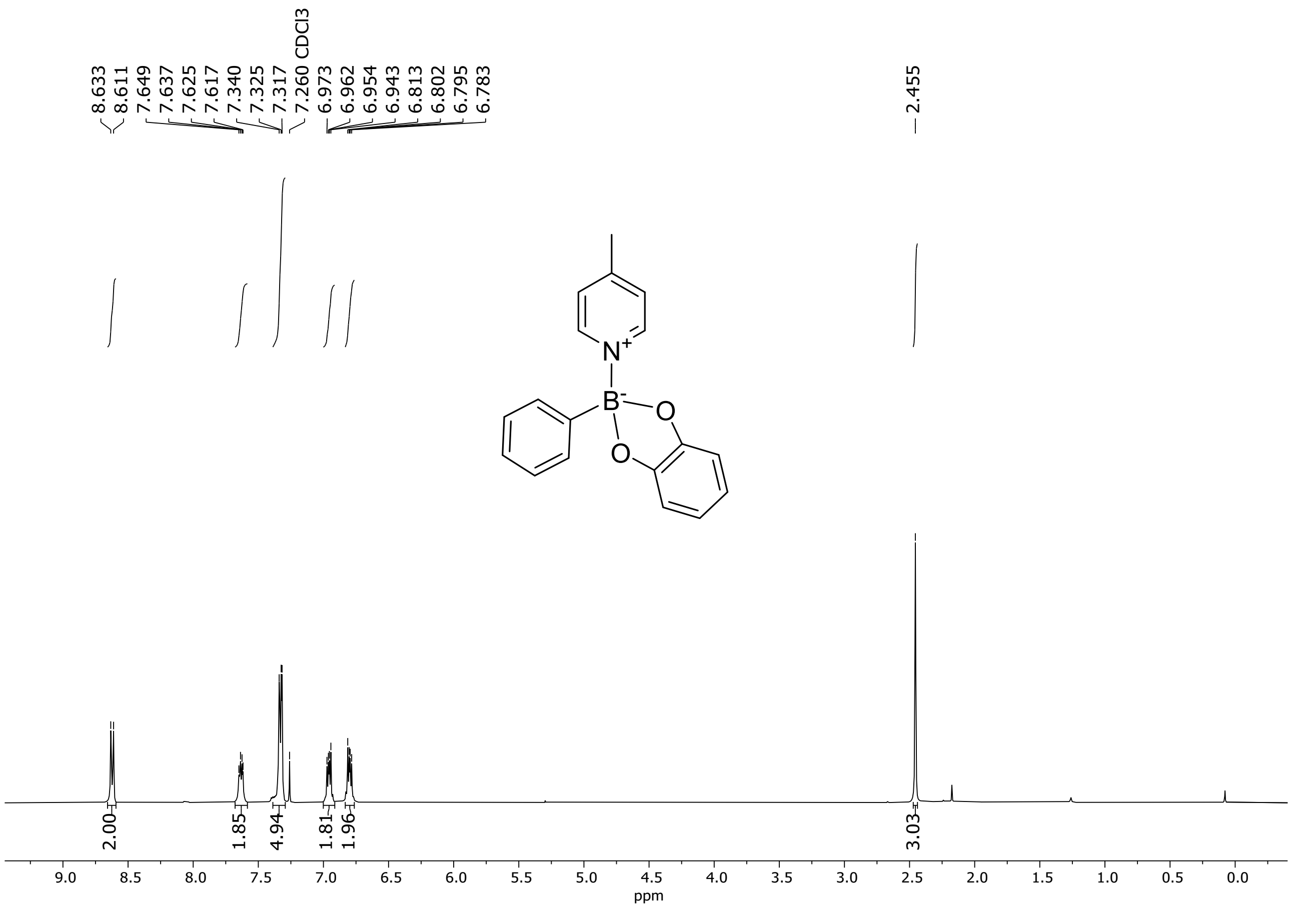

Figure S5. ^{1}H NMR spectrum of a solution containing **1** and **3** (both 0,42 M in $CDCl_3$, 25° C. Expected association 91%).

^{1}H NMR (300 MHz, $CDCl_3$, 25°C) δ 8.62 (d, J^1 = 6 Hz, 2H), 7.63 (m, 2H), 7.32 (m, 5H), 6.96 (m, 2H), 6.80 (m, 2H), 2.46 (s, 3H) ppm.

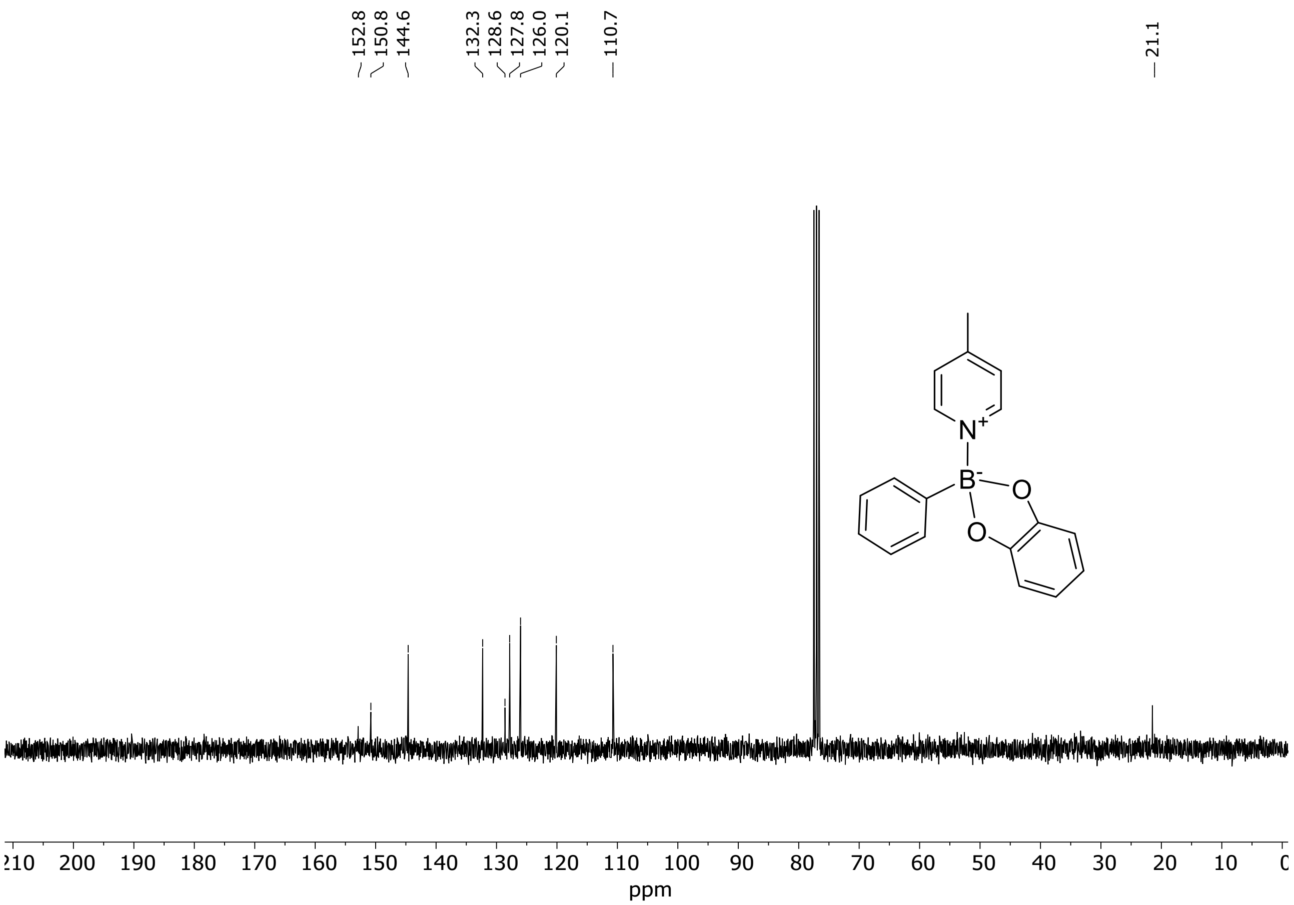

Figure S6. ^{13}C NMR spectrum of a solution containing **1** and **3** (both 0,42 M in $CDCl_3$, 25° C. Expected association 91%).

^{13}C NMR (75 MHz, $CDCl_3$, 25°C) δ 152.8, 150.8, 144.6, 132.3, 128.6, 127.8, 126.0, 120.1, 110.7, 21.1 ppm.

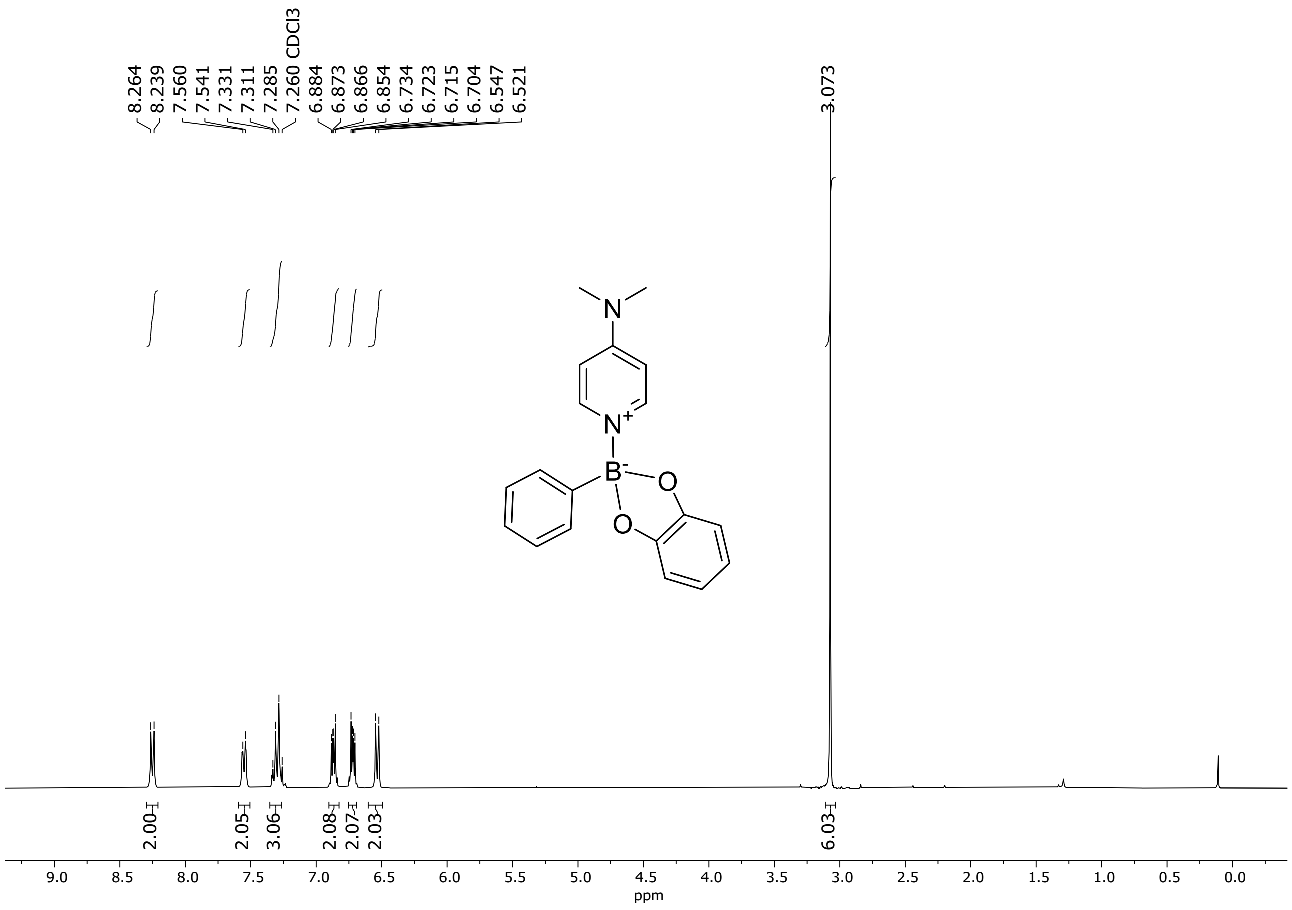

Figure S7. ^1H NMR spectrum of a solution containing **1** and **5** (both 0,42 M in $CDCl_3$, 25° C. Expected association >99%).

^1H NMR (300 MHz, $CDCl_3$, 25°C) δ 8.25 (d, J^1 = 6 Hz, 2H), 7.55 (d, J^1 = 6 Hz, 2H), 7.30 (m, 3H), 6.87 (m, 2H), 6.72 (m, 2H), 6.53 (d, J^1 = 9 Hz, 2H), 3.07 (s, 6H) ppm.

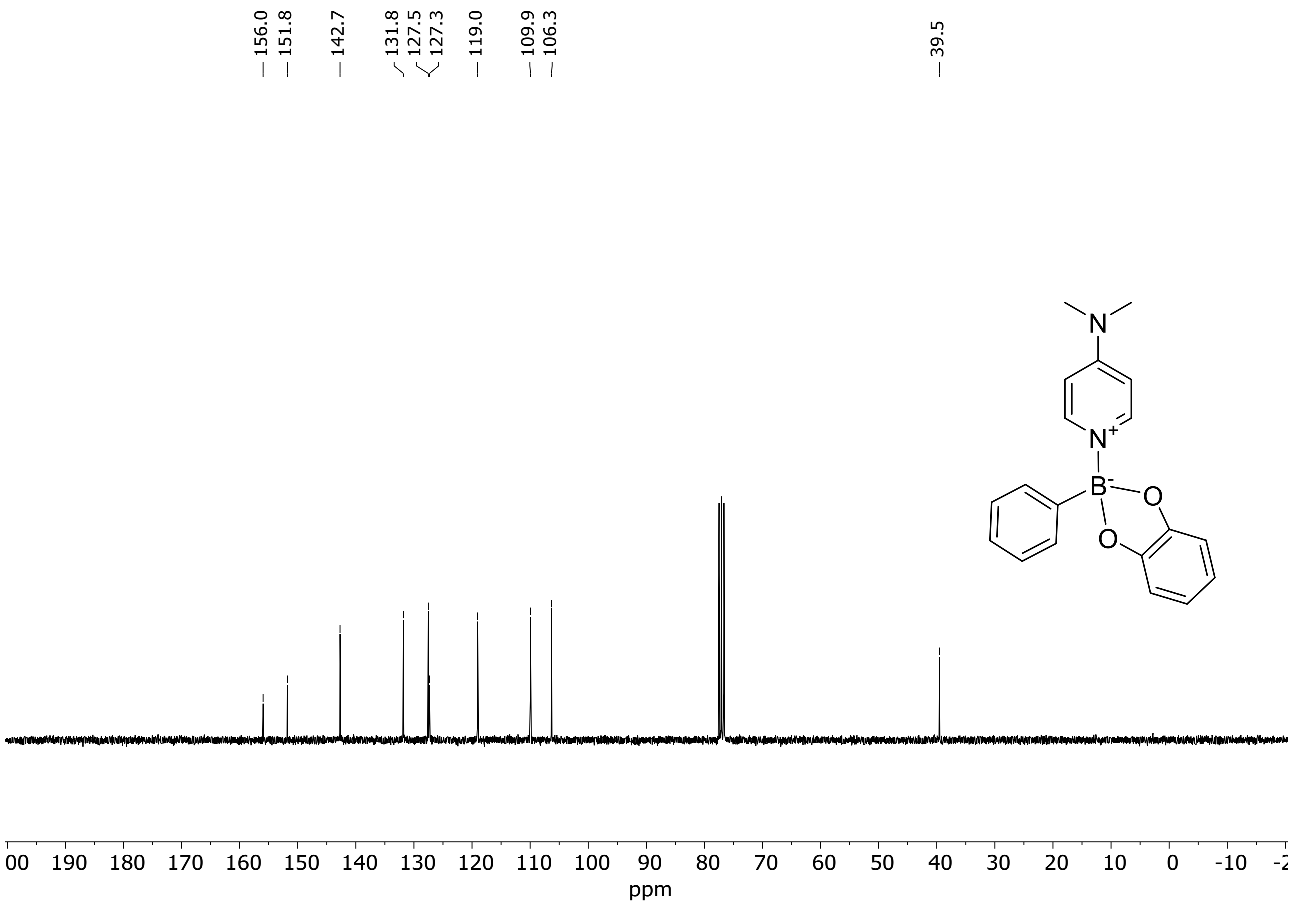

Figure S8. ^{13}C NMR spectrum of a solution containing **1** and **5** (both 0,42 M in $CDCl_3$, 25° C. Expected association >99%).

^{13}C NMR (75 MHz, $CDCl_3$, 25°C) δ 156.0, 151.8, 142.7, 131.8, 127.5, 127.3, 119.0, 109.9, 106.3, 39.5 ppm.

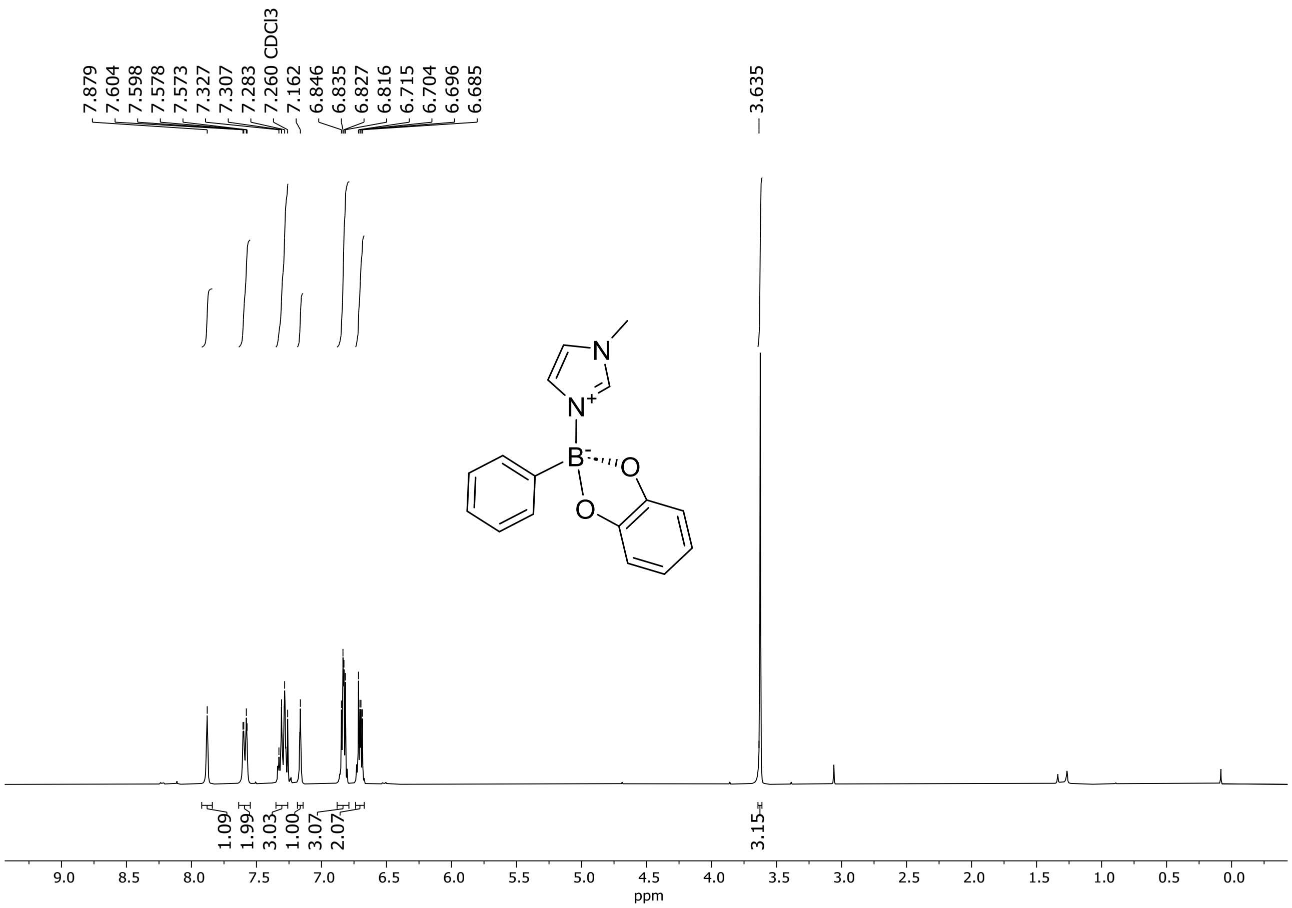

Figure S9. ^1H NMR spectrum of a solution containing **1** and **4**(both 0,42 M in $CDCl_3$, 25° C. Expected association 97%).

^1H NMR (300 MHz, $CDCl_3$, 25°C) δ 7.88 (s, 1H), 7.59 (m, 2H), 7.30 (m, 3H), 7.16 (s, 1H), 6.83 (m, 3H), 6.70 (m, 2H), 3.63 (s, 3H) ppm.

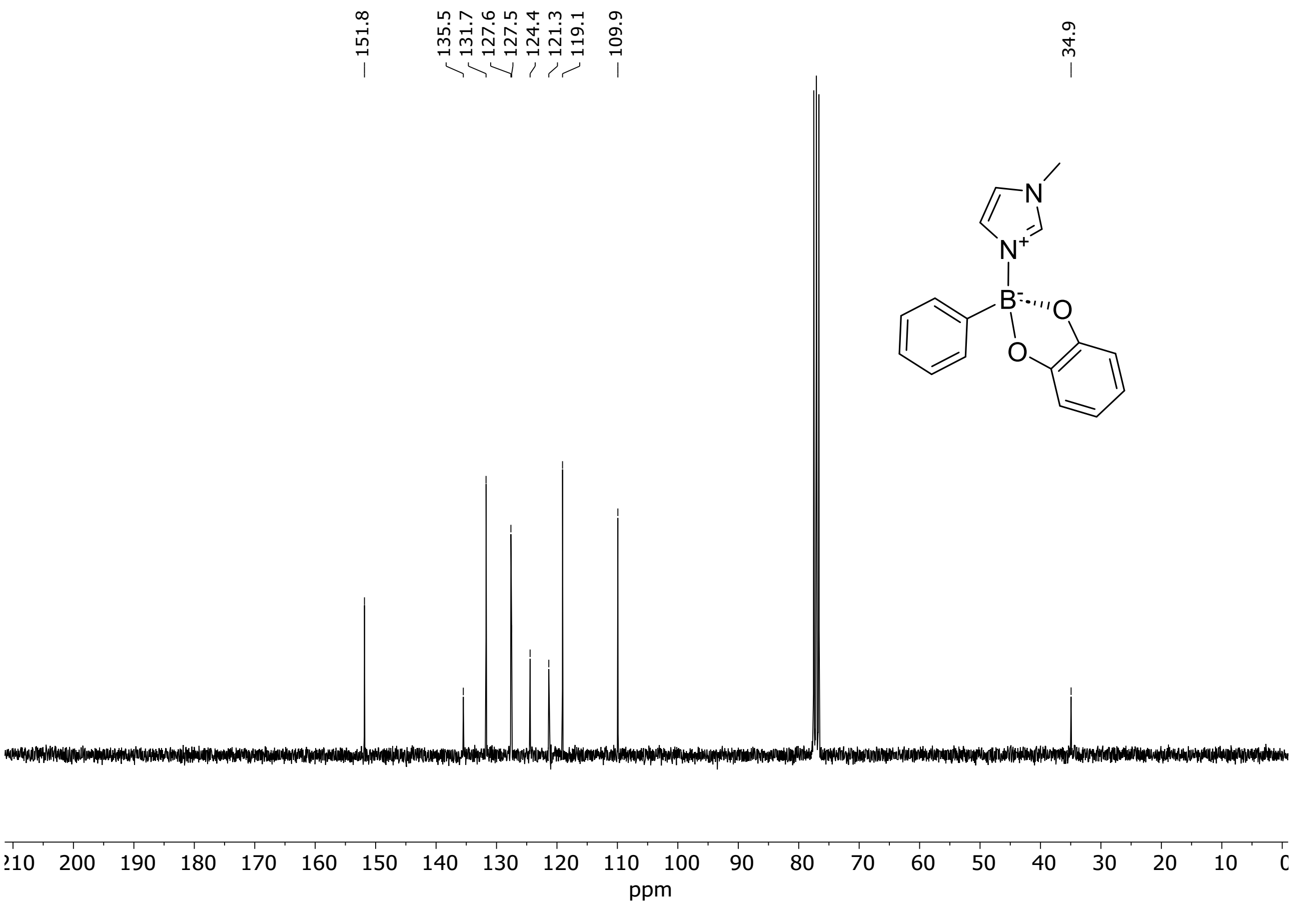

Figure S10. ^{13}C NMR of a solution containing **1** and **4** (both 0,42 M in $CDCl_3$, 25° C. Expected association 97%).

^{13}C NMR (75 MHz, $CDCl_3$, 25°C) δ 151.8, 135.5, 131.7, 127.6, 127.5, 124.4, 121.3, 119.1, 109.9, 34.9 ppm.

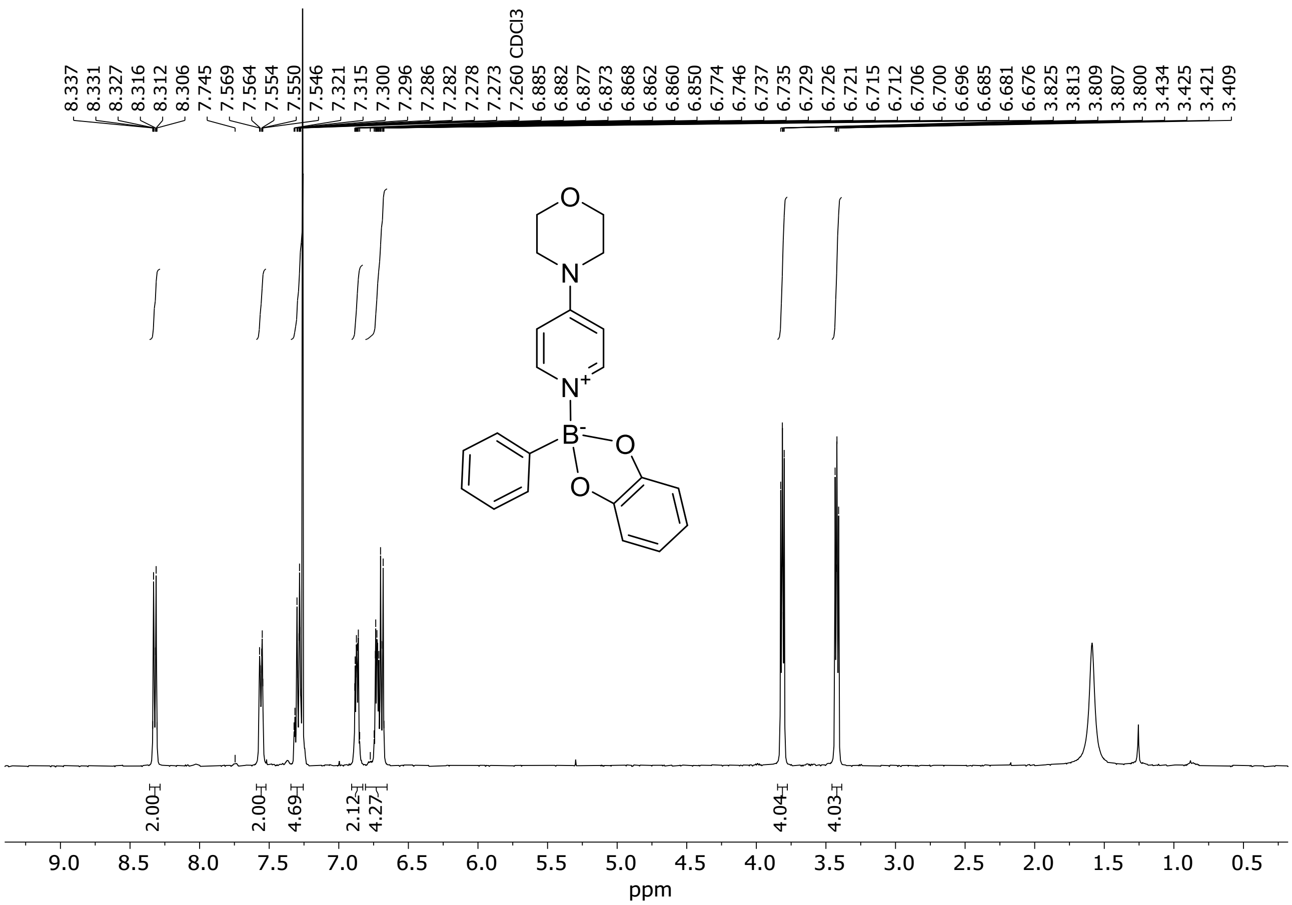

Figure S11. ^{1}H NMR of a solution containing **1** and **6** (both 0.012 M in $CDCl_3$, 25° C. Expected association 90%)

^{1}H NMR (400 MHz, $CDCl_3$) δ 8.36 – 8.28 (m, 2H), 7.59 – 7.52 (m, 2H), 7.34 – 7.26 (m, 5H), 6.87 (ddt, J = 7.4, 5.0, 2.5 Hz, 2H), 6.81 – 6.65 (m, 4H), 3.85 – 3.78 (m, 4H), 3.42 (dd, J = 5.9, 4.2 Hz, 4H).

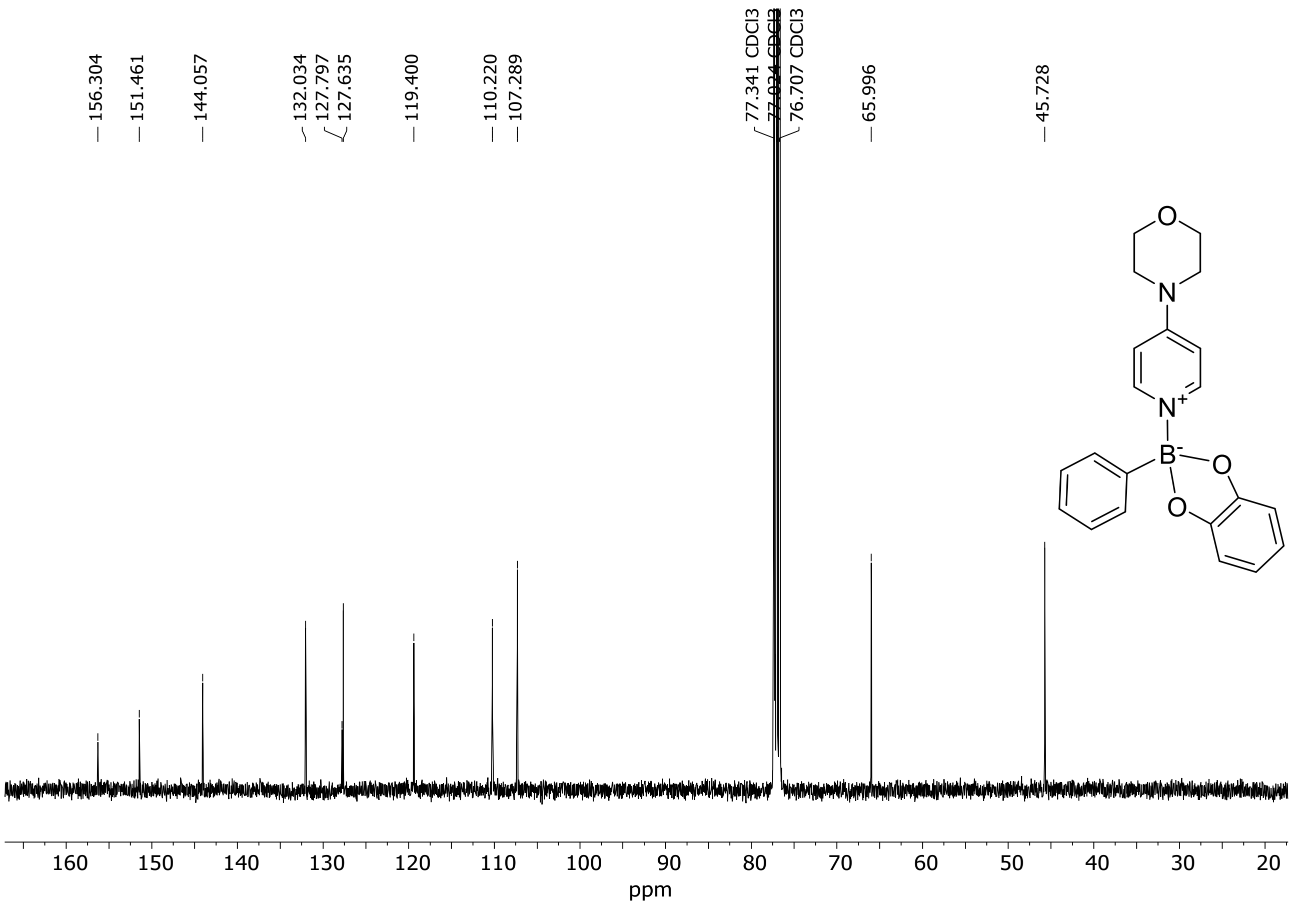

Figure S12. ^{13}C NMR of a solution containing **1** and **6** (both 0.100 M in $CDCl_3$, 25° C. Expected association 90%)

^{13}C NMR (101 MHz, $CDCl_3$) δ 156.30, 151.46, 144.06, 132.03, 127.80, 127.64, 119.40, 110.22, 107.29, 77.34, 77.02, 76.71, 66.00, 45.73.

NMR titration experiments

For each titration, 5.00 mM **1** was titrated with compounds **2 - 11** fitting a 1:1 binding isotherm to the experimental points. The binding curve of the various adducts is generated plotting the chemical shift of the ^{1}H NMR signal of proton H_{ortho} of the phenylboronic moiety versus the molar equivalents of the *N*ArHet added. It is apparent that the more basic the *N*ArHet, the greater binding constant. Indeed, the value of the pK_aH^{+2} of the *N*ArHets is linearly correlated to the logarithm of the binding constant (Figure S33).

SigmaPlot version 15 for Windows was employed for all the fits, using the following code for a 1:1 binding model:

```
f = Po+(DP*K*H)/(1+K*H)


H=x-g

g=(-b-(sqrt((b^2)-4*c)))/2

c=C*x

b=-C-x-1/K

Po = 8.09

C=5e-3


fit f to y
```

With initial parameters:

```
K = 1000

DP = 0
```

And constraints:

```
K > 0
```

The initial chemical shift was always 8.09 ppm for free phenylboronic acid cathecol ester, and all the titrations were performed keeping its total concentration fixed at 5 mM.

For amines **7-11**, we attributed the slight differences in the NMR spectra to the formation of ion pairs between **1**·OH^{-} and the different protonated amines.

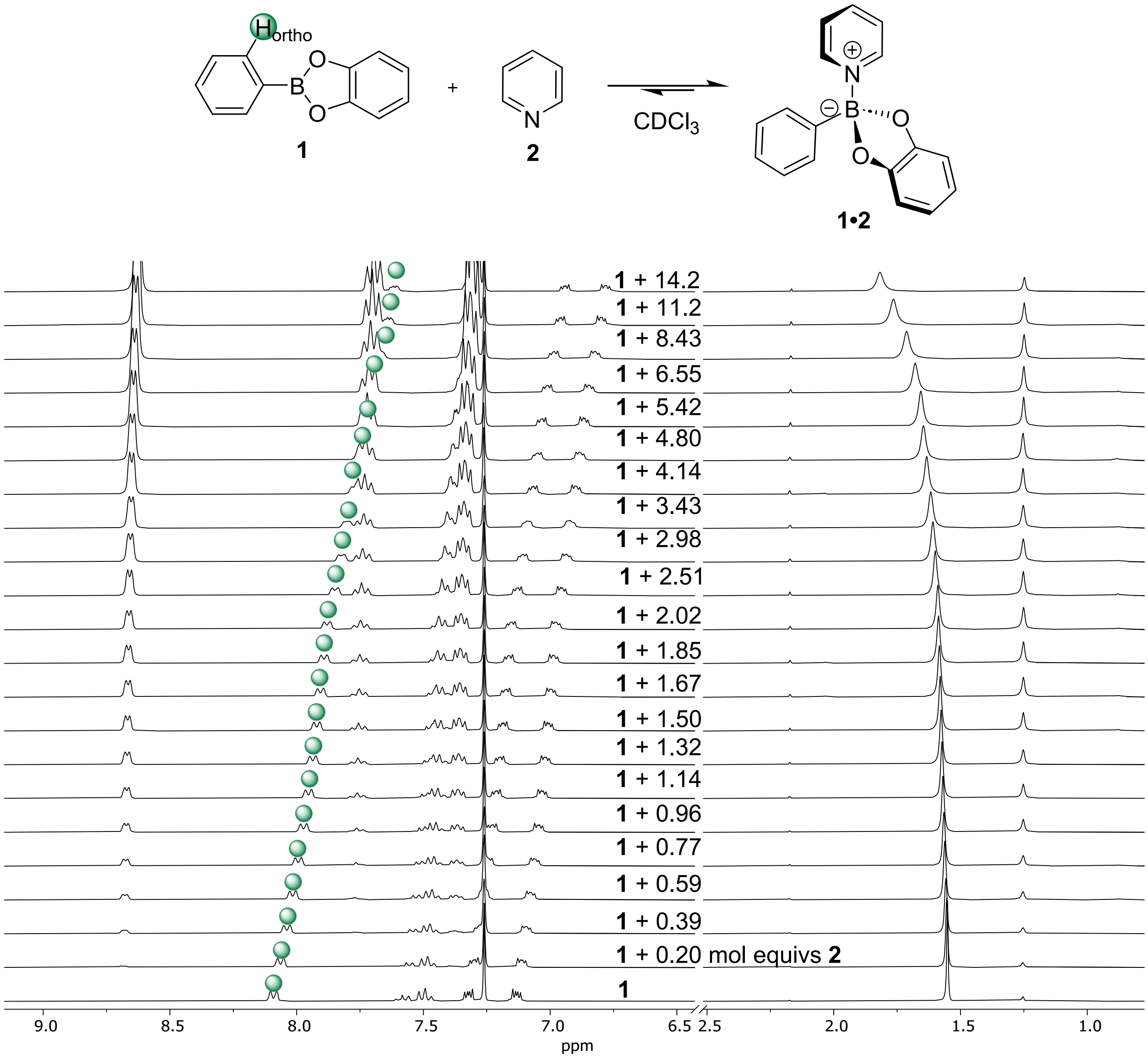

Figure S13. Top: reaction scheme for the ^{1}H NMR titration of **1** with pyridine (**2**). Bottom: spectra. The H_{ortho} proton signal is highlighted with a green sphere on the spectra.

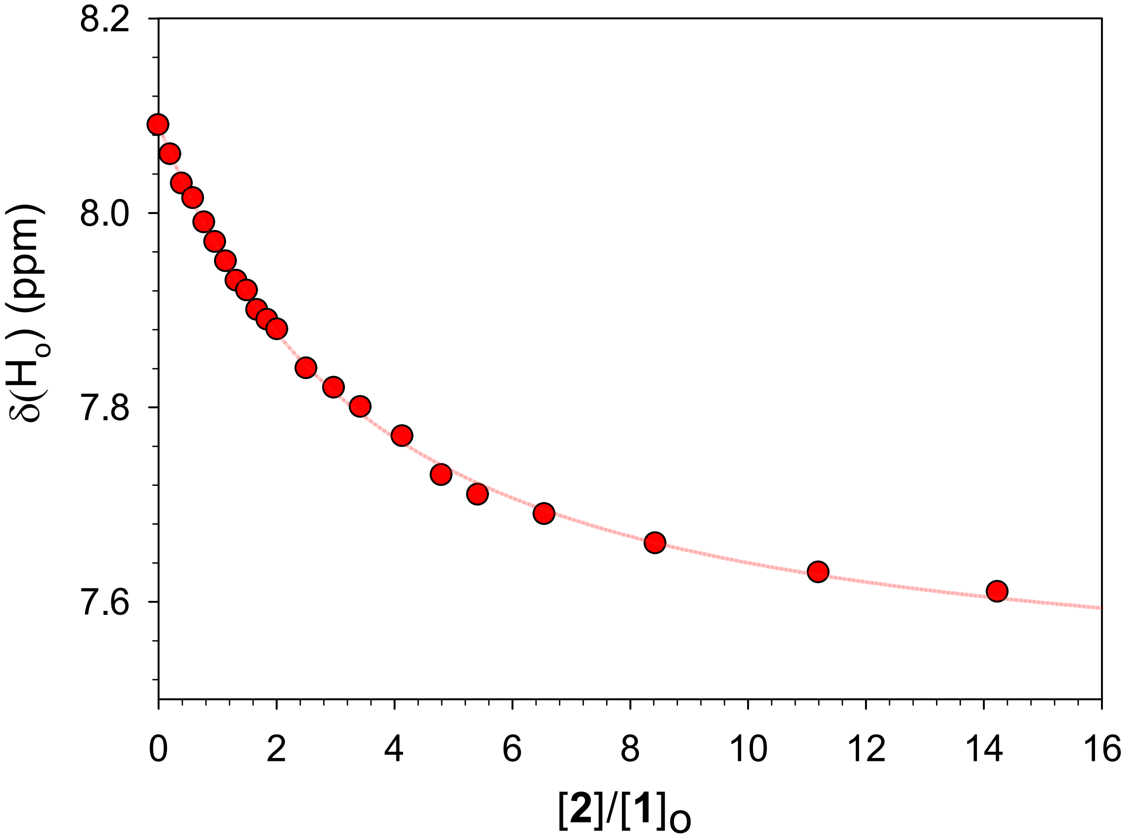

Figure S14. Chemical shift of the H_{ortho} proton on **1** upon addition of pyridine (**2**).

$K_{bind} = 68\ M^{-1}$

Extrapolated chemical shift at saturation δH_{ortho}: 7.50 ppm.

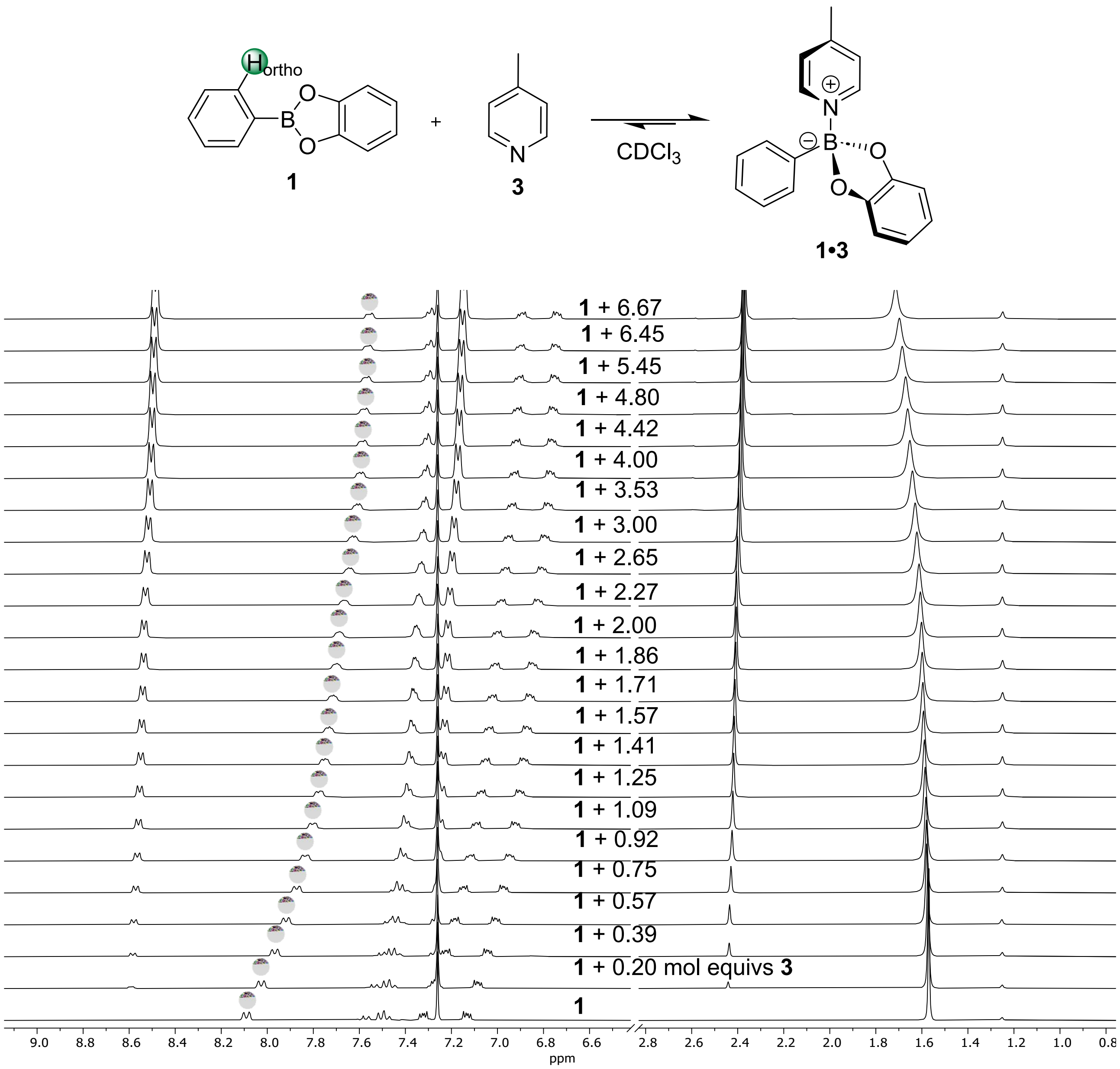

Figure S15. Top: reaction scheme for the ^{1}H NMR titration of **1** with 4-methylpyridine (**3**). Bottom: spectra. The H_{ortho} proton signal is highlighted with a green sphere on the spectra.

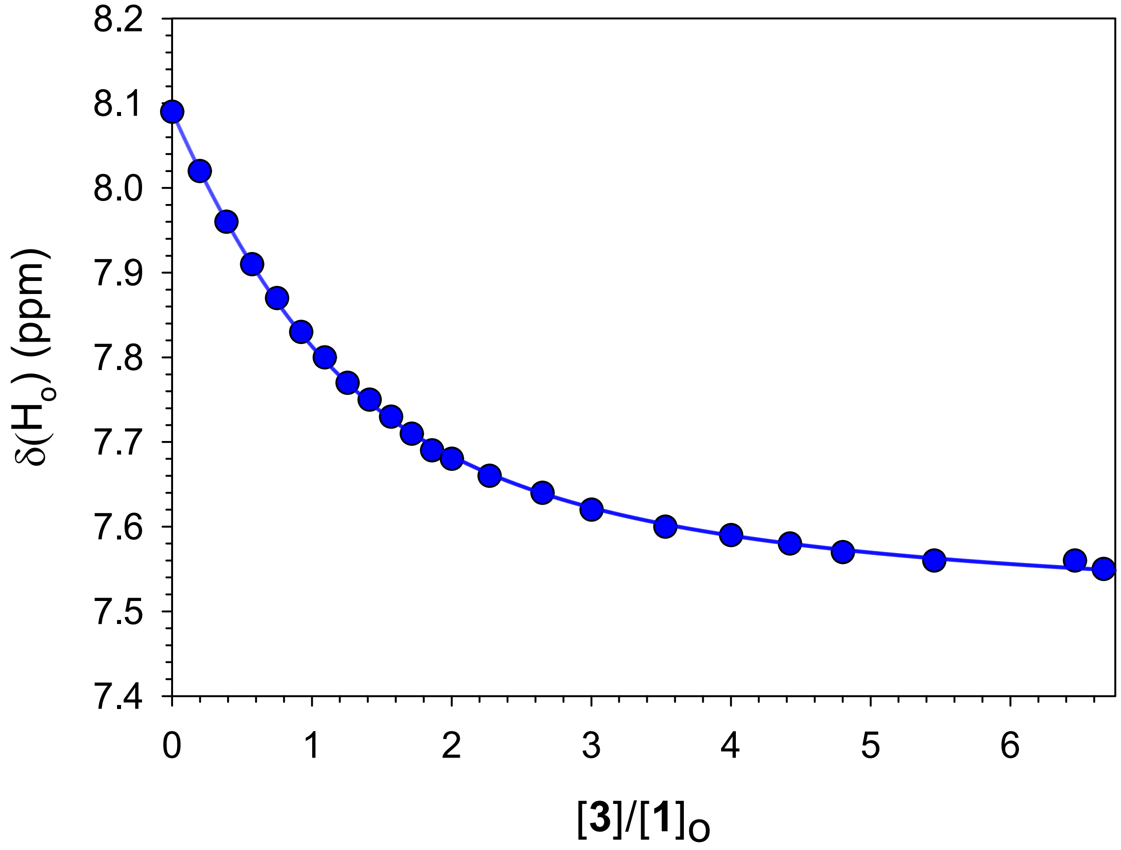

Figure S16. Chemical shift of the H_{ortho} proton on **1** upon addition of 4-methyl pyridine (**3**).

$K_{bind} = 300\ M^{-1}$

Extrapolated chemical shift at saturation δH_{ortho}: 7.50 ppm.

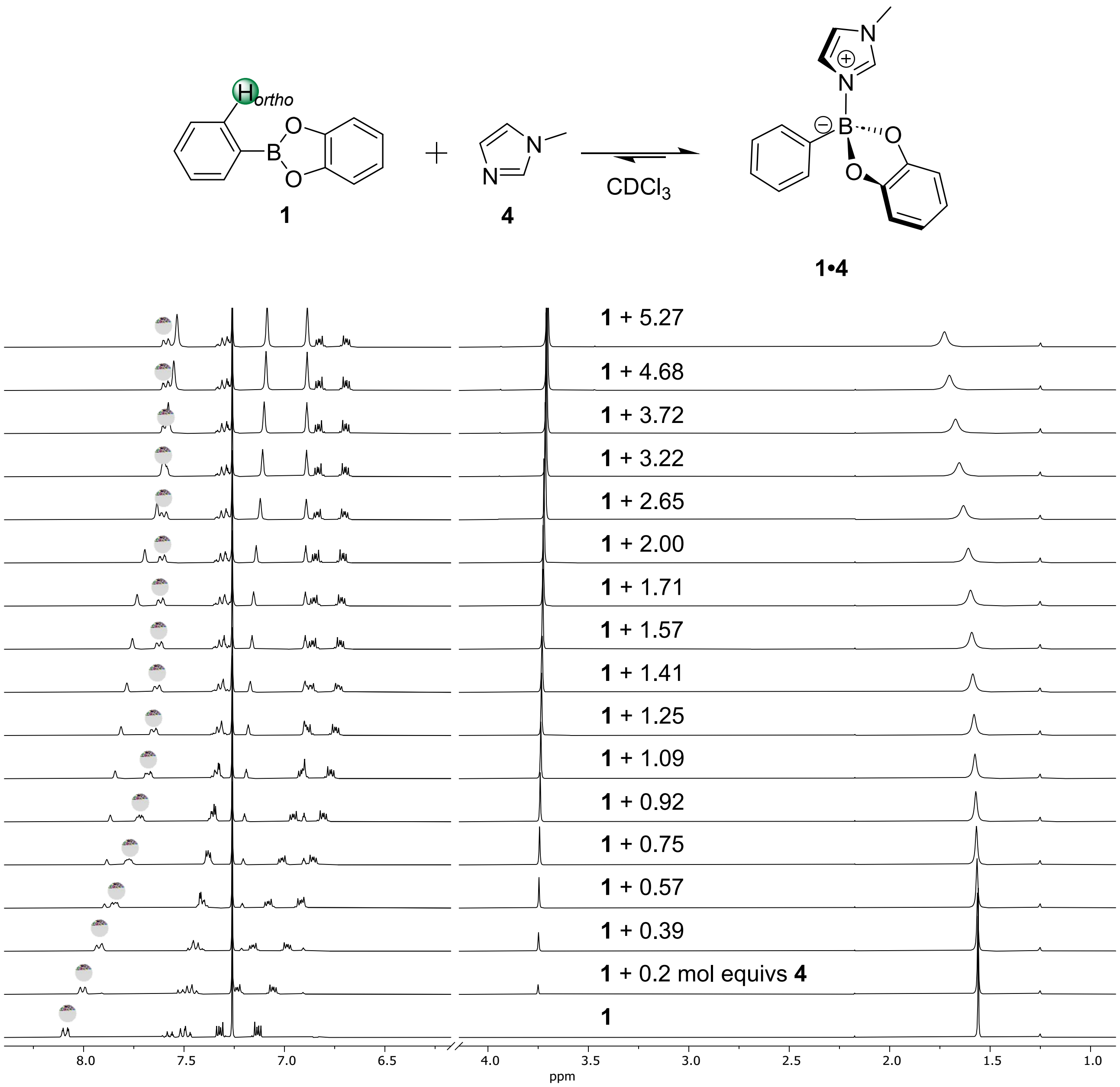

Figure S17. Top: reaction scheme for the ^{1}H NMR titration of **1** with 1-methylimidazole (**4**). Bottom: spectra. Further addition of **4** resulted in no change to the chemical shift of the H_{ortho} proton (signal highlighted by a green sphere on the spectra).

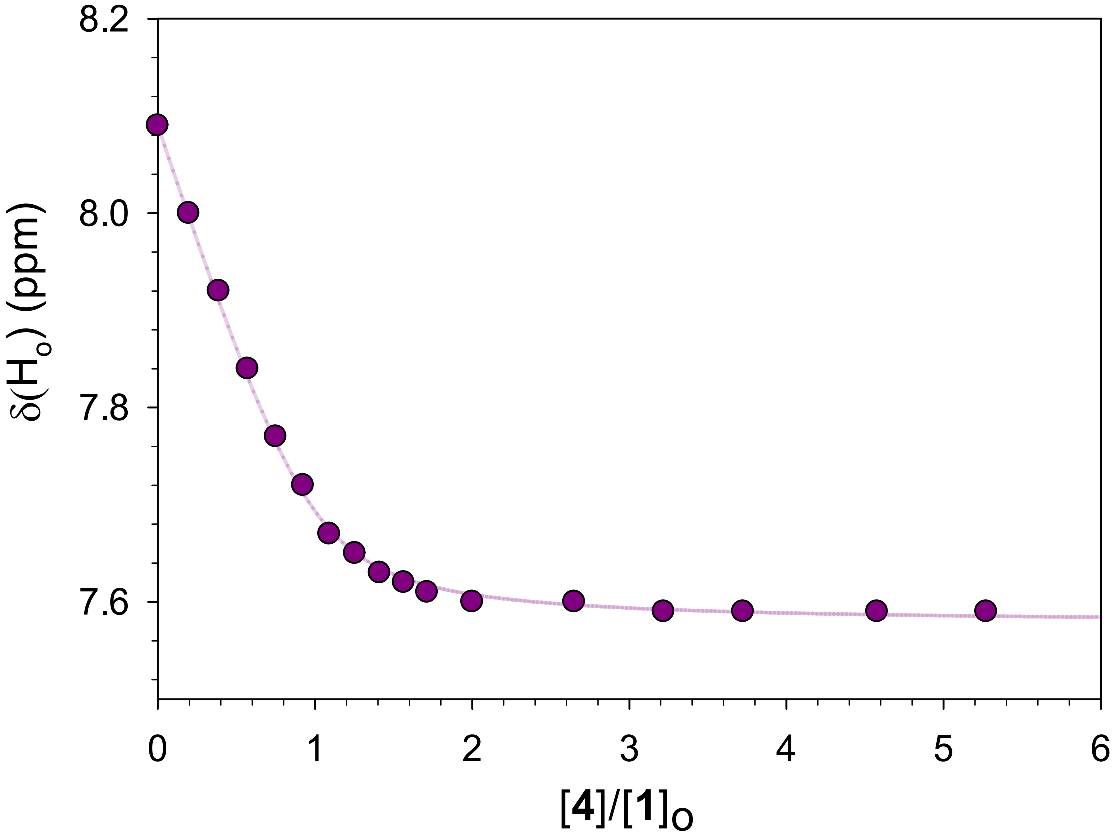

Figure S18. Chemical shift of the H_{ortho} proton on **1** upon addition of 1-methylimidazole (**4**).

$K_{bind} = 3.0 \cdot 10^3 \, M^{-1}$

Extrapolated chemical shift at saturation δH_{ortho}: 7.57 ppm.

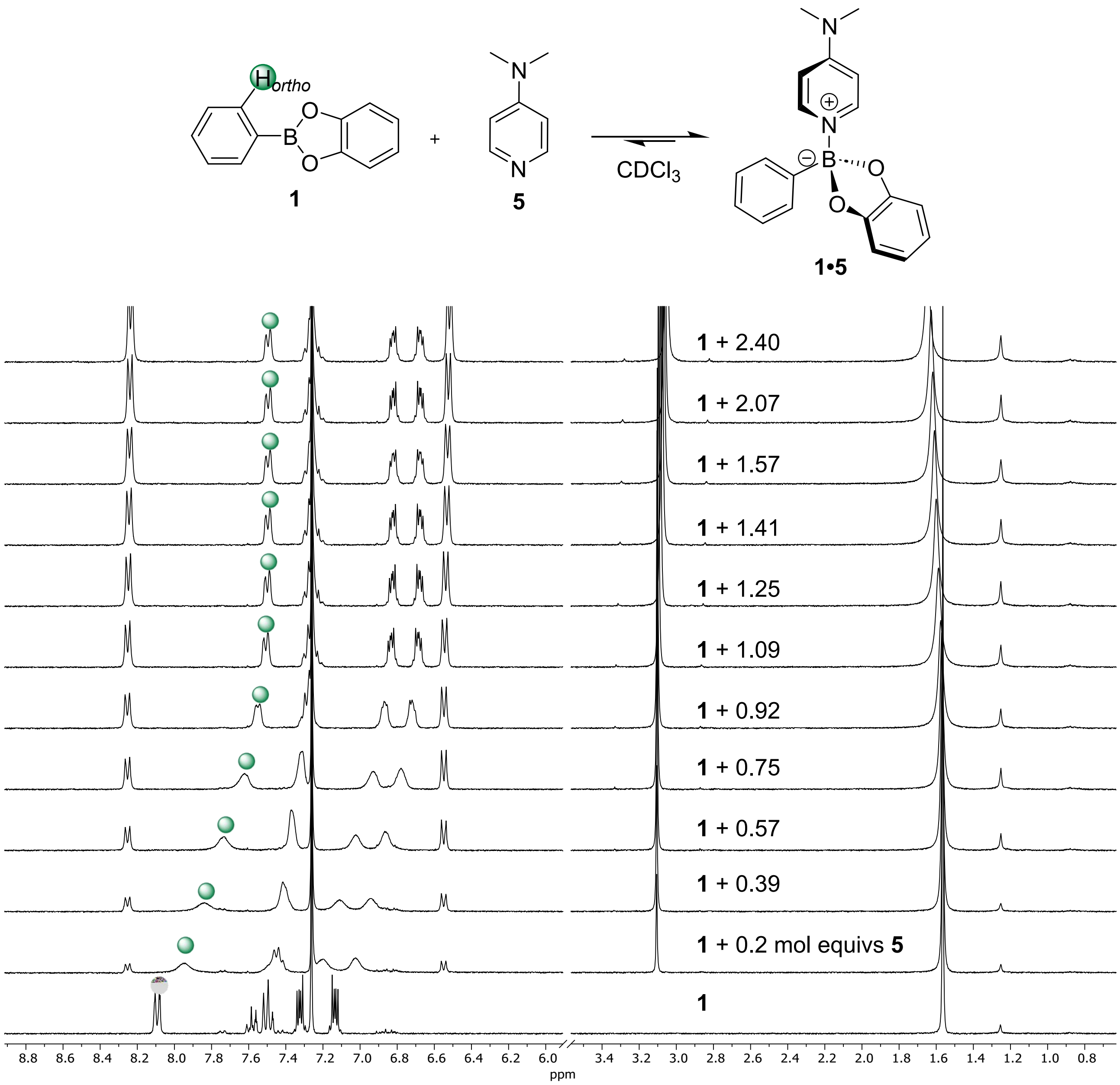

Figure S19. Top: reaction scheme for the ^{1}H NMR titration of **1** with 4-(dimethylamino)pyridine (**5**). Bottom: spectra. Further addition of **5** resulted in no change to the chemical shift of the H_{ortho} proton (signal highlighted by a green sphere on the spectra).

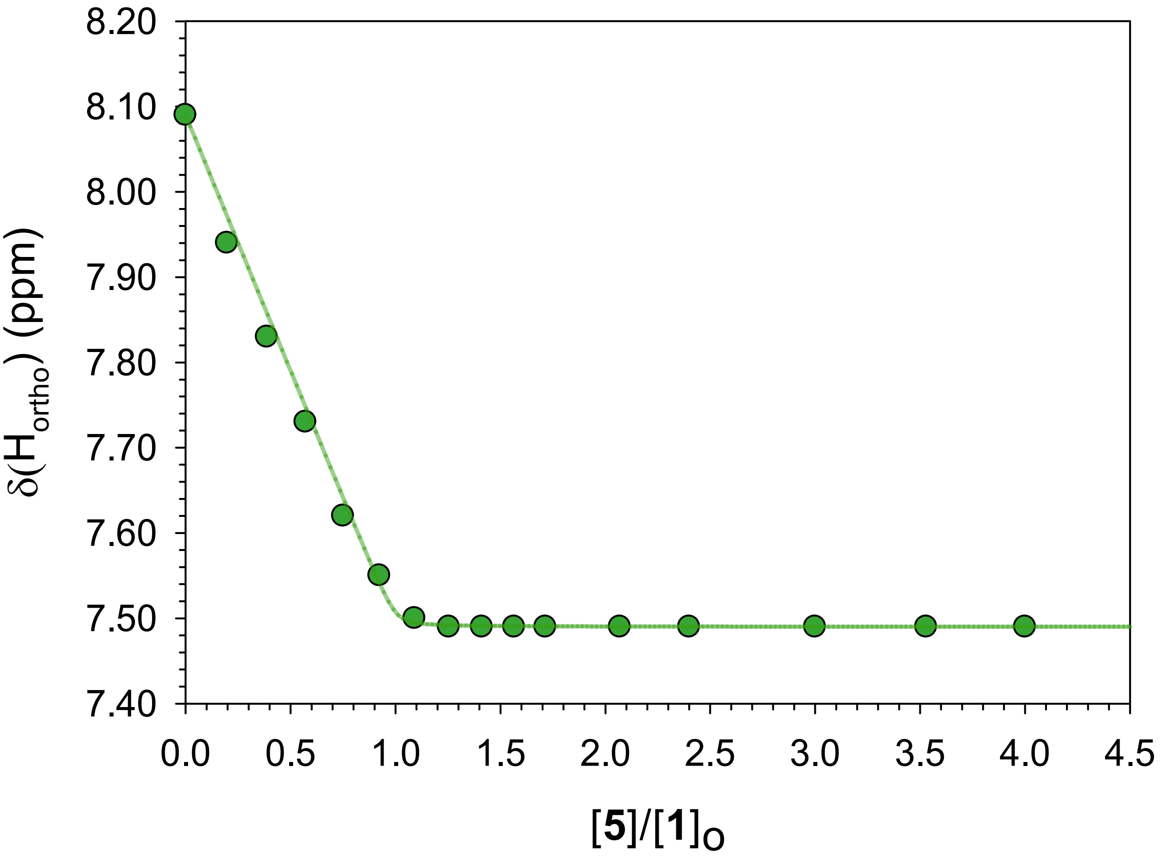

Figure S20. Chemical shift of the H_{ortho} proton on **1** upon addition of 4-dimethylamino pyridine (**5**).

$K_{bind} = 9 \cdot 10^5\ M^{-1}$

Extrapolated chemical shift at saturation δH_{ortho}: 7.50 ppm.

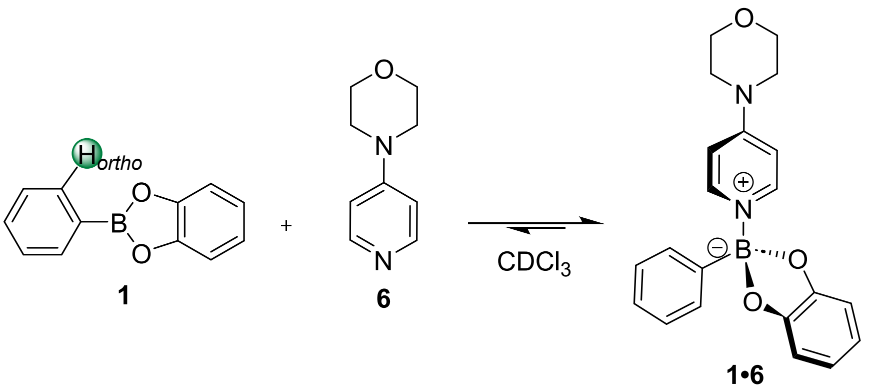

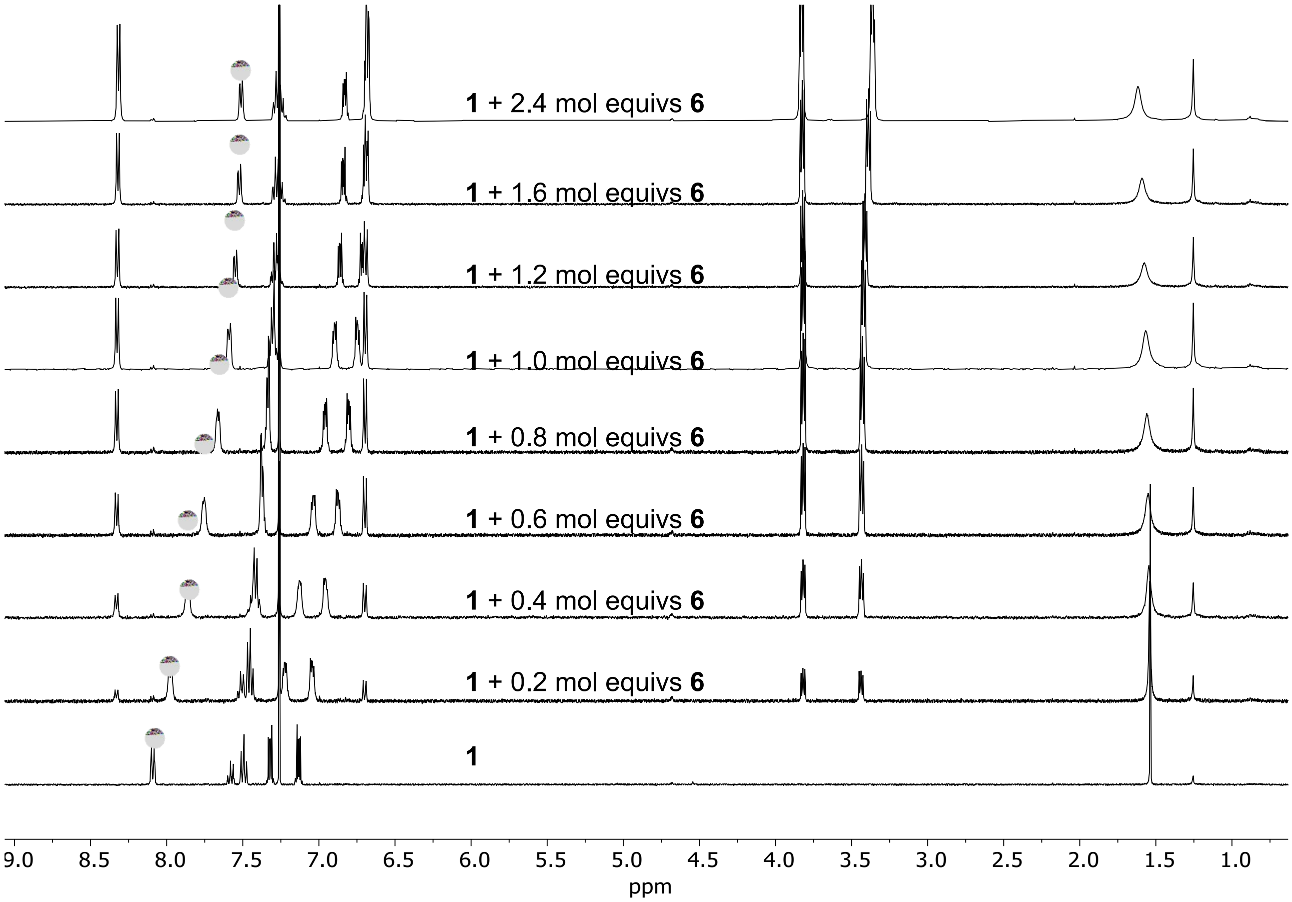

Figure S21. Top: reaction scheme for the ^{1}H NMR titration of **1** with 4-(pyridin-4-yl)morpholine (**6**). Bottom: spectra. The H_{ortho} proton signal is highlighted by a green sphere on the spectra.

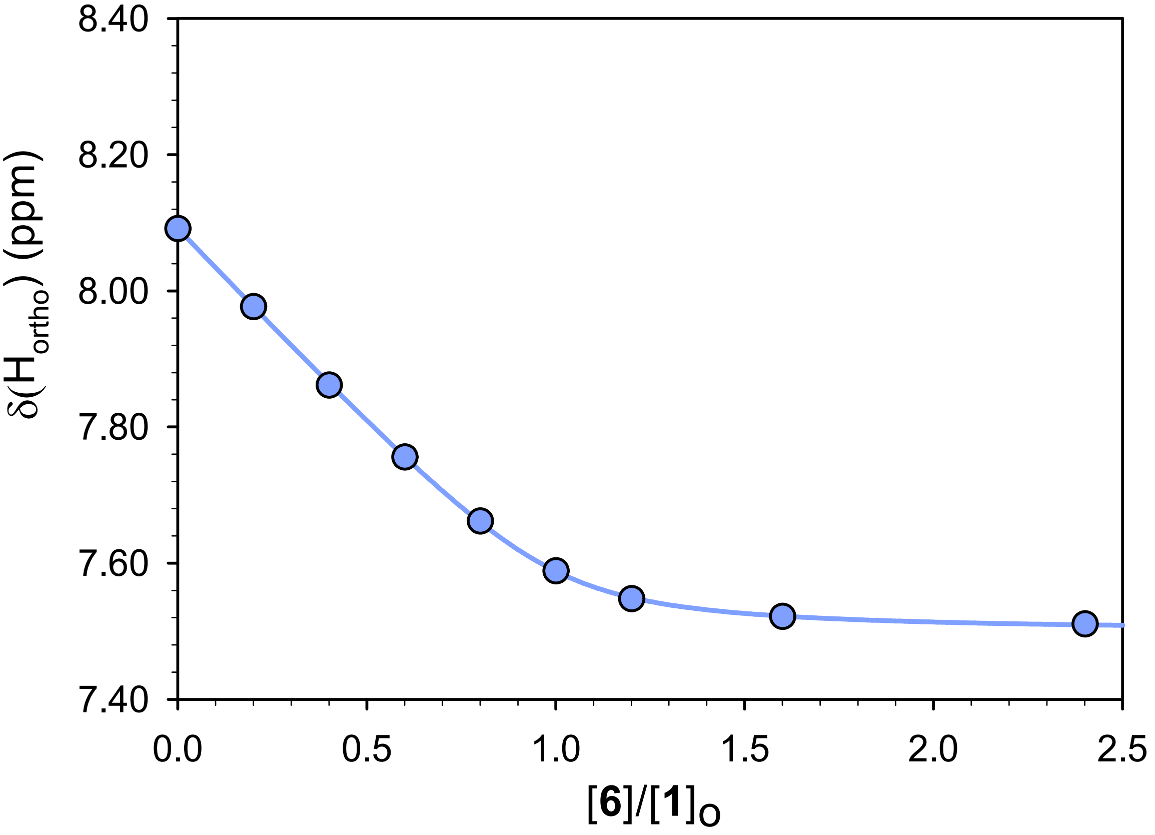

Figure S22. Chemical shift of the H_{ortho} proton on **1** upon addition of 4-(pyridin-4-yl)morpholine (**6**). $K_{bind} = 7.4 \cdot 10^3\ M^{-1}$

Extrapolated chemical shift at saturation δH_{ortho}: 7.50 ppm.

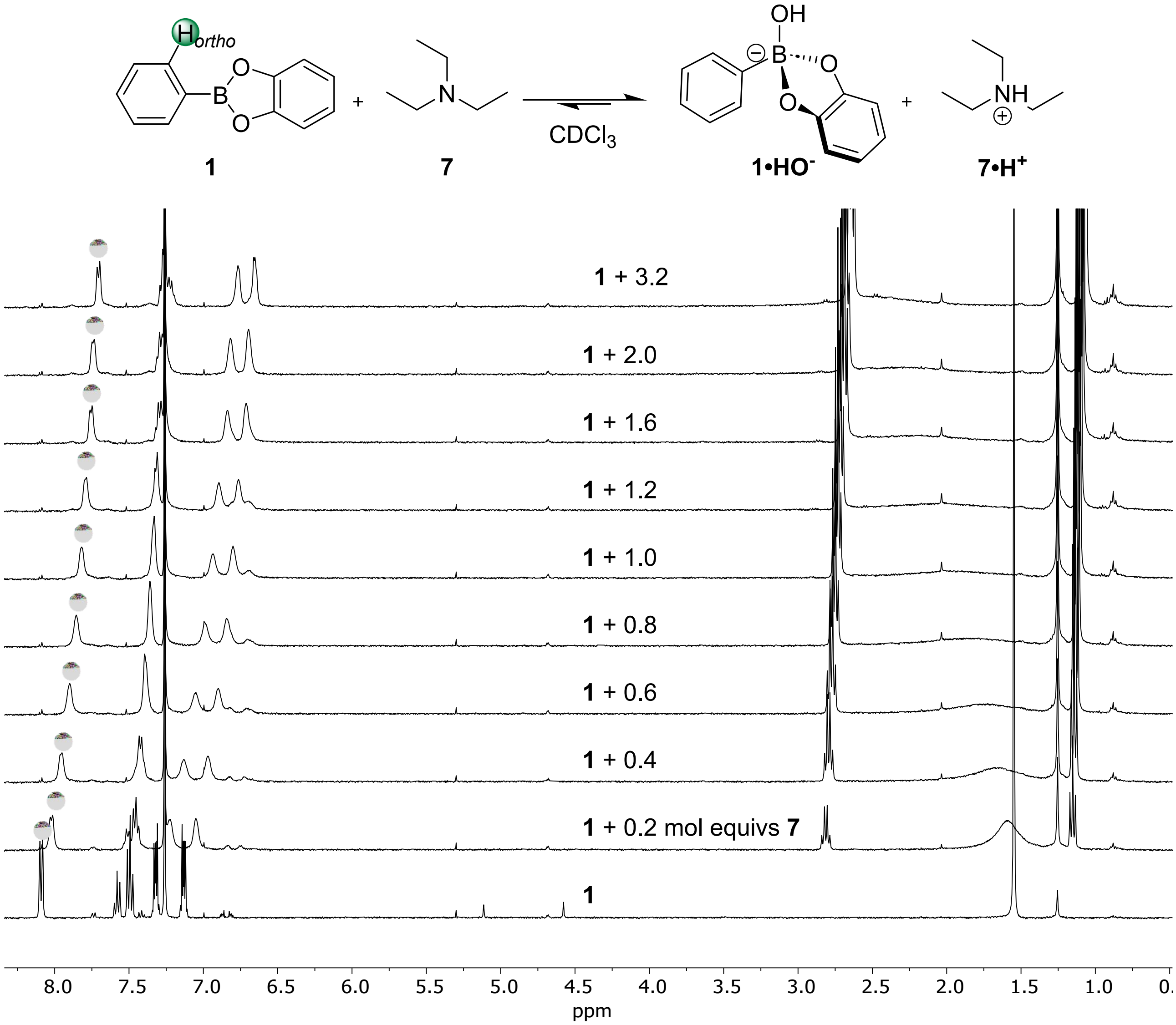

Figure S23. Top: reaction scheme for the ^{1}H NMR titration of **1** with triethylamine (**7**). Bottom: spectra. The H_{ortho} proton is highlighted by a green sphere on the spectra.

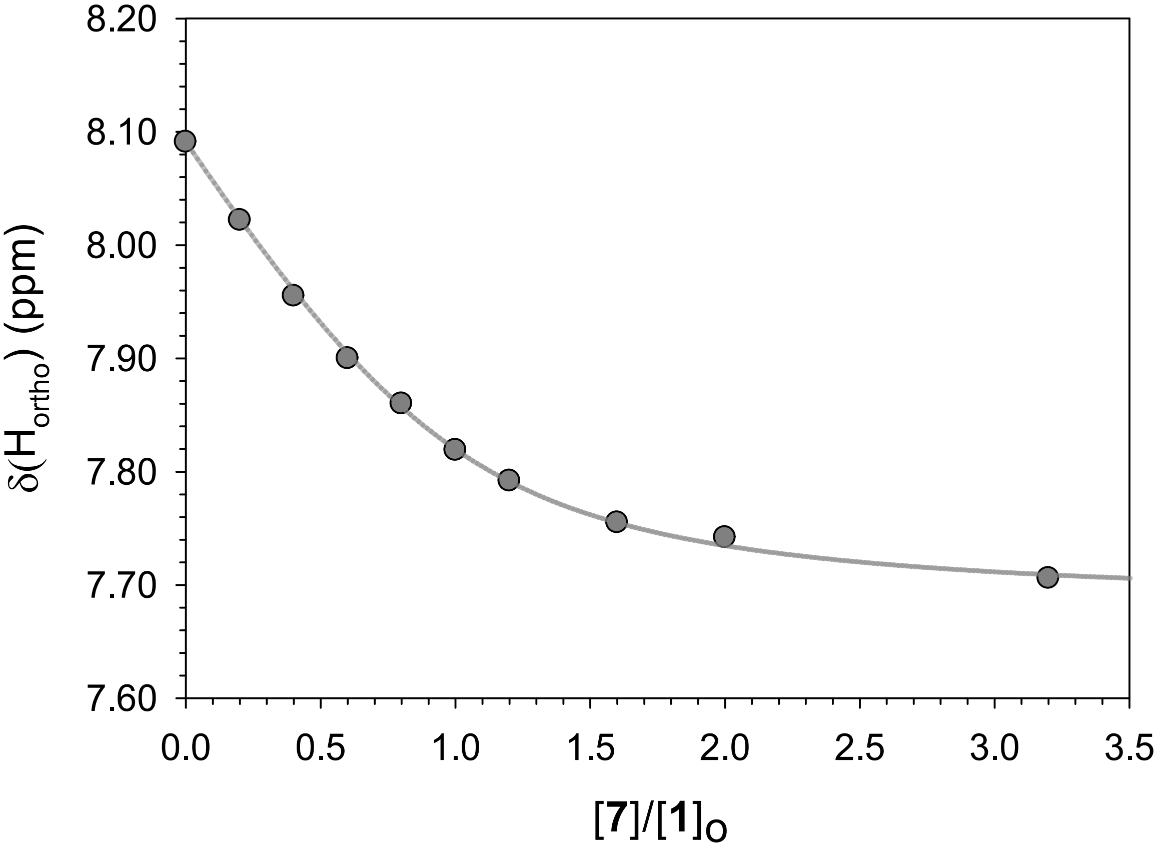

Figure S24. Chemical shift of the H_{ortho} proton on **1** upon addition of triethylamine (**7**).

$K_{obs} = 1100\ M^{-1}$

Extrapolated chemical shift at saturation δH_{ortho}: 7.69 ppm.

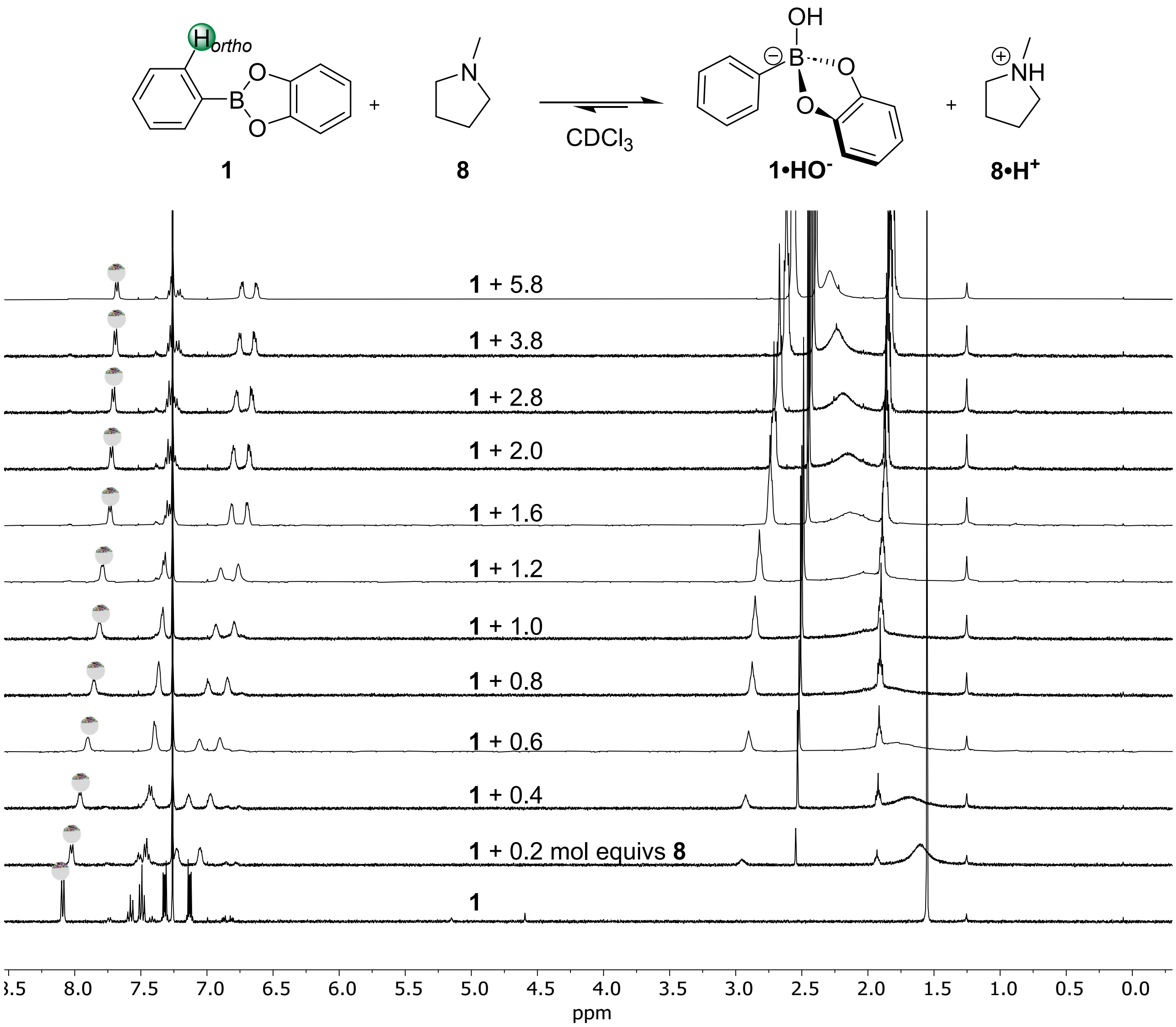

Figure S25. Top: reaction scheme for the ^{1}H NMR titration of **1** with *N*-methylpyrrolidine (**8**). Bottom: spectra. The H_{ortho} proton signal is highlighted by a green sphere on the spectra.

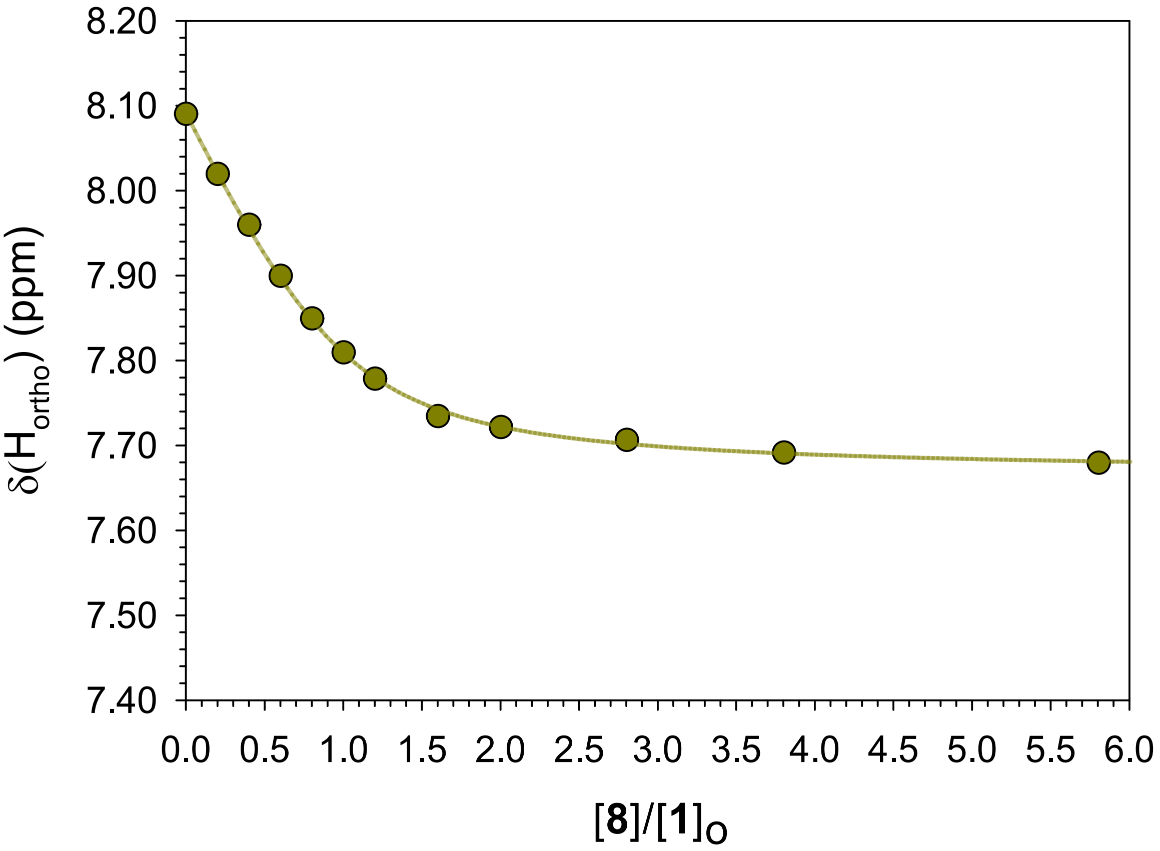

Figure S26. Chemical shift of the H_{ortho} proton on **1** upon addition of N-methylpyrrolidine (**8**).

$K_{obs} = 1200\ M^{-1}$

Extrapolated chemical shift at saturation δH_{ortho}: 7.68 ppm.

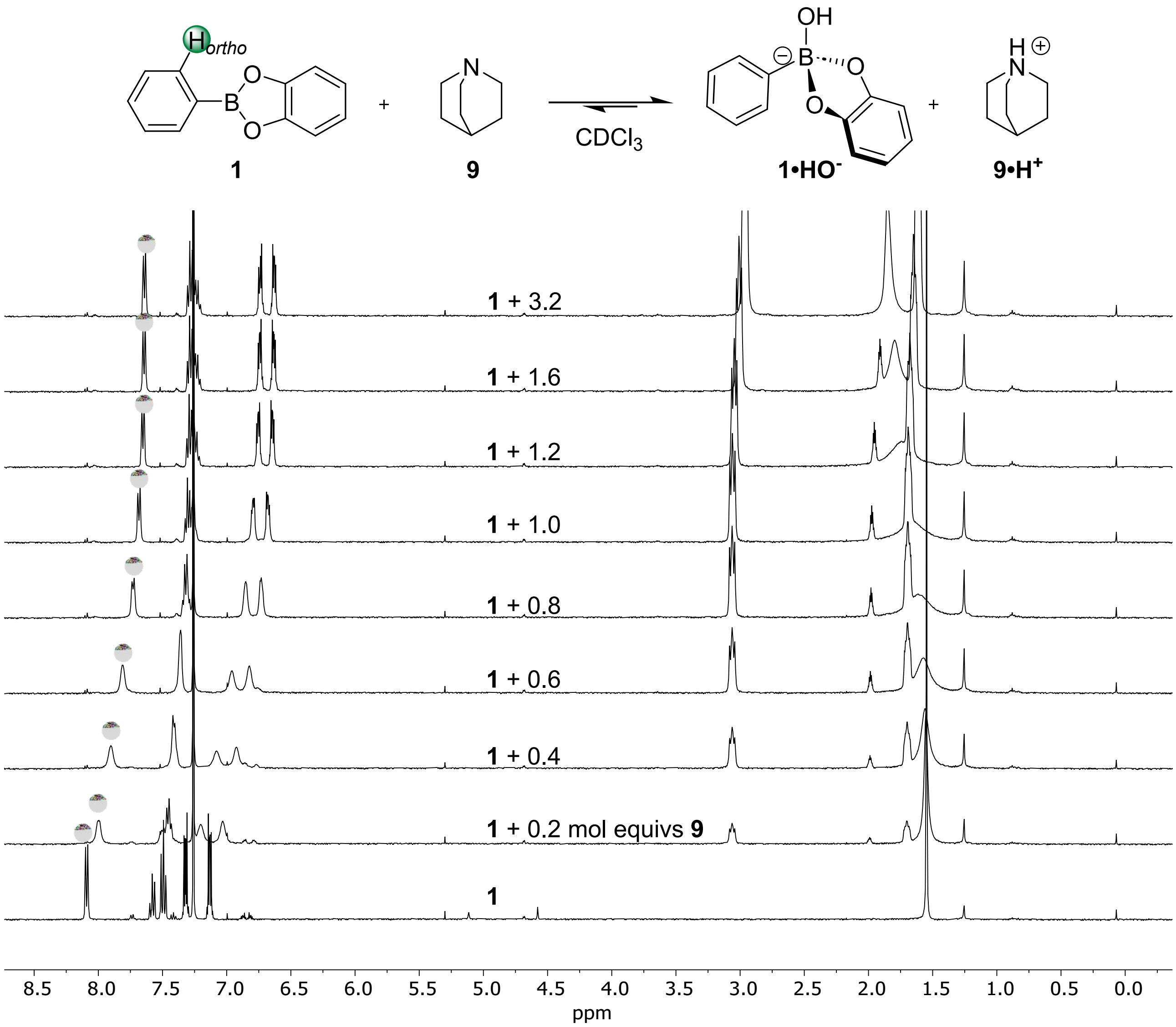

Figure S27. Top: reaction scheme for the ^{1}H NMR titration of **1** with quinuclidine (**9**). Bottom: spectra. Further addition of **9** resulted in no change to the chemical shift of the H_{ortho} proton (signal highlighted by a green sphere on the spectra).

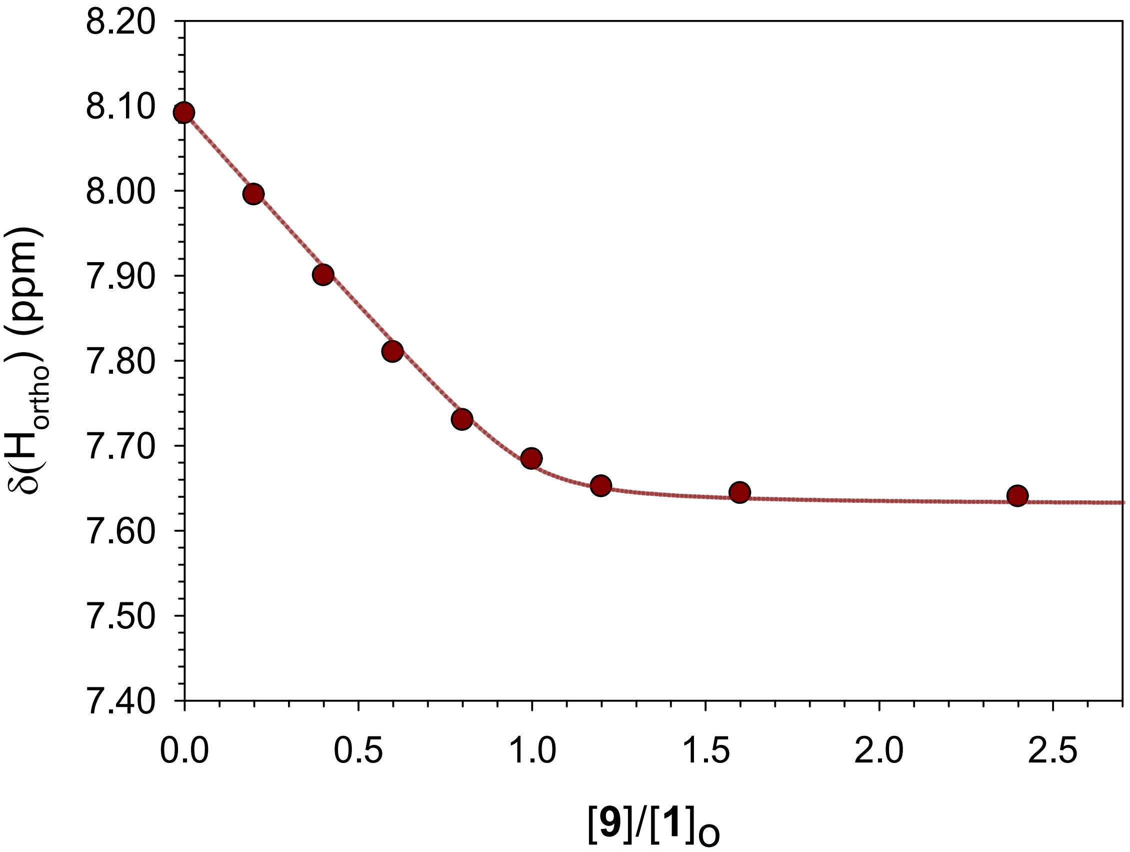

Figure S28. Chemical shift of the H_{ortho} proton on **1** upon addition of quinuclidine (**9**).

$K_{obs} = 1.8 \cdot 10^4\ M^{-1}$

Extrapolated chemical shift at saturation δH_{ortho}: 7.64 ppm.

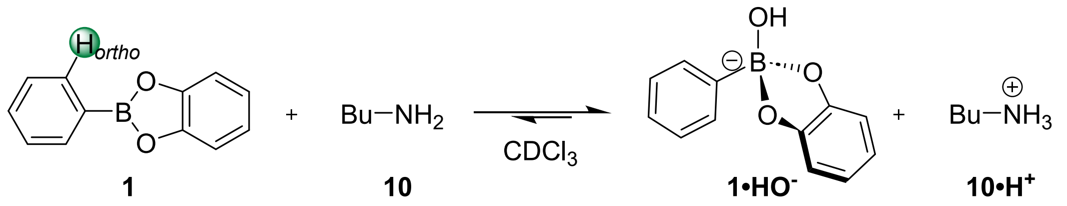

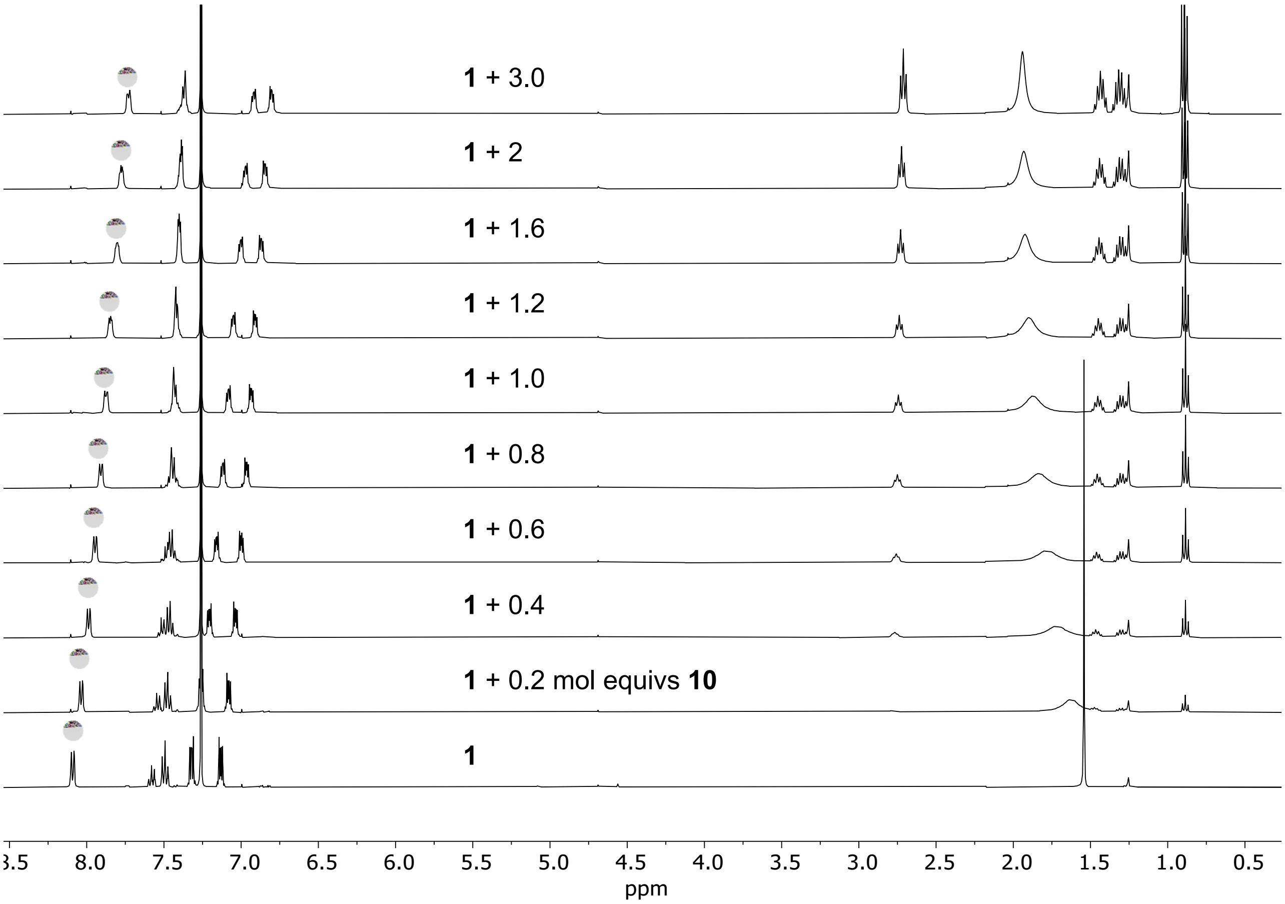

Figure S29. Top: reaction scheme for the ^{1}H NMR titration of **1** with butylamine (**10**). Bottom: spectra. The H_{ortho} proton signal is highlighted by a green sphere on the spectra.

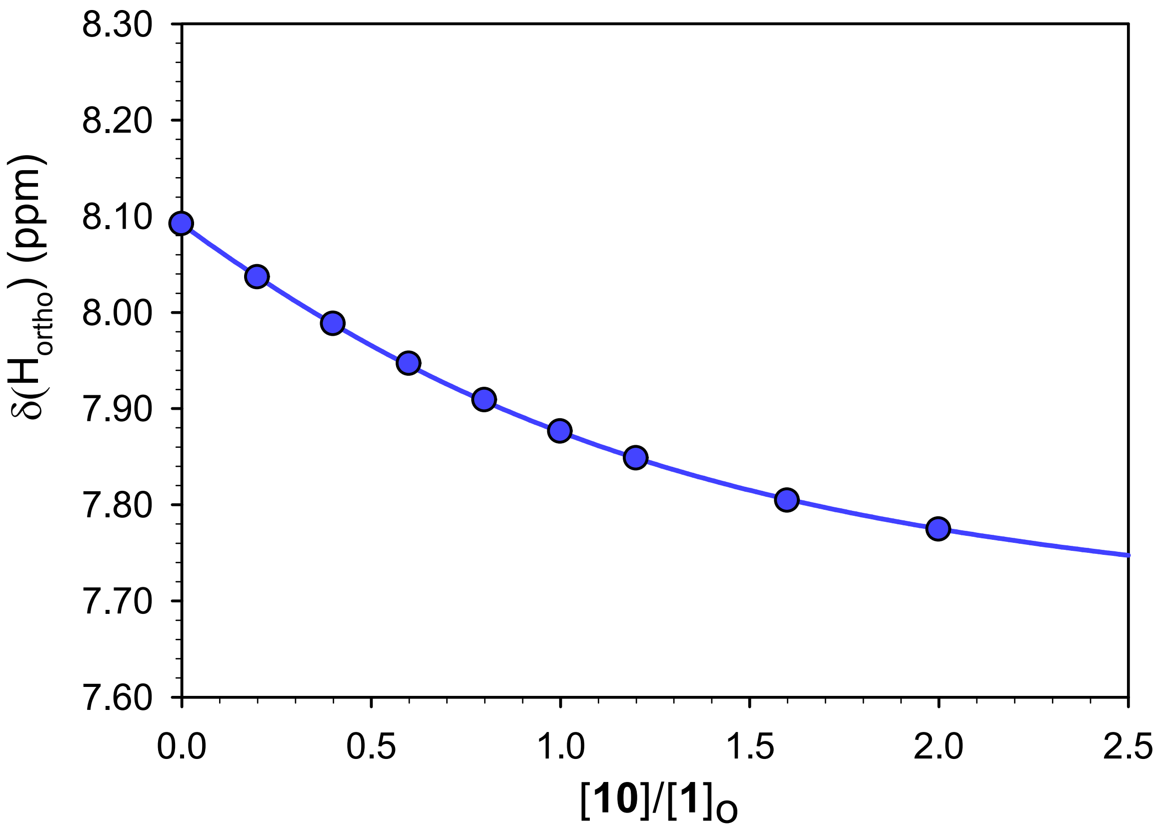

Figure S30. Chemical shift of the H_{ortho} proton on **1** upon addition of butylamine (**10**).

$$K_{obs} = 330\ M^{-1}$$

Extrapolated chemical shift at saturation δH_{ortho}: 7.63 ppm.

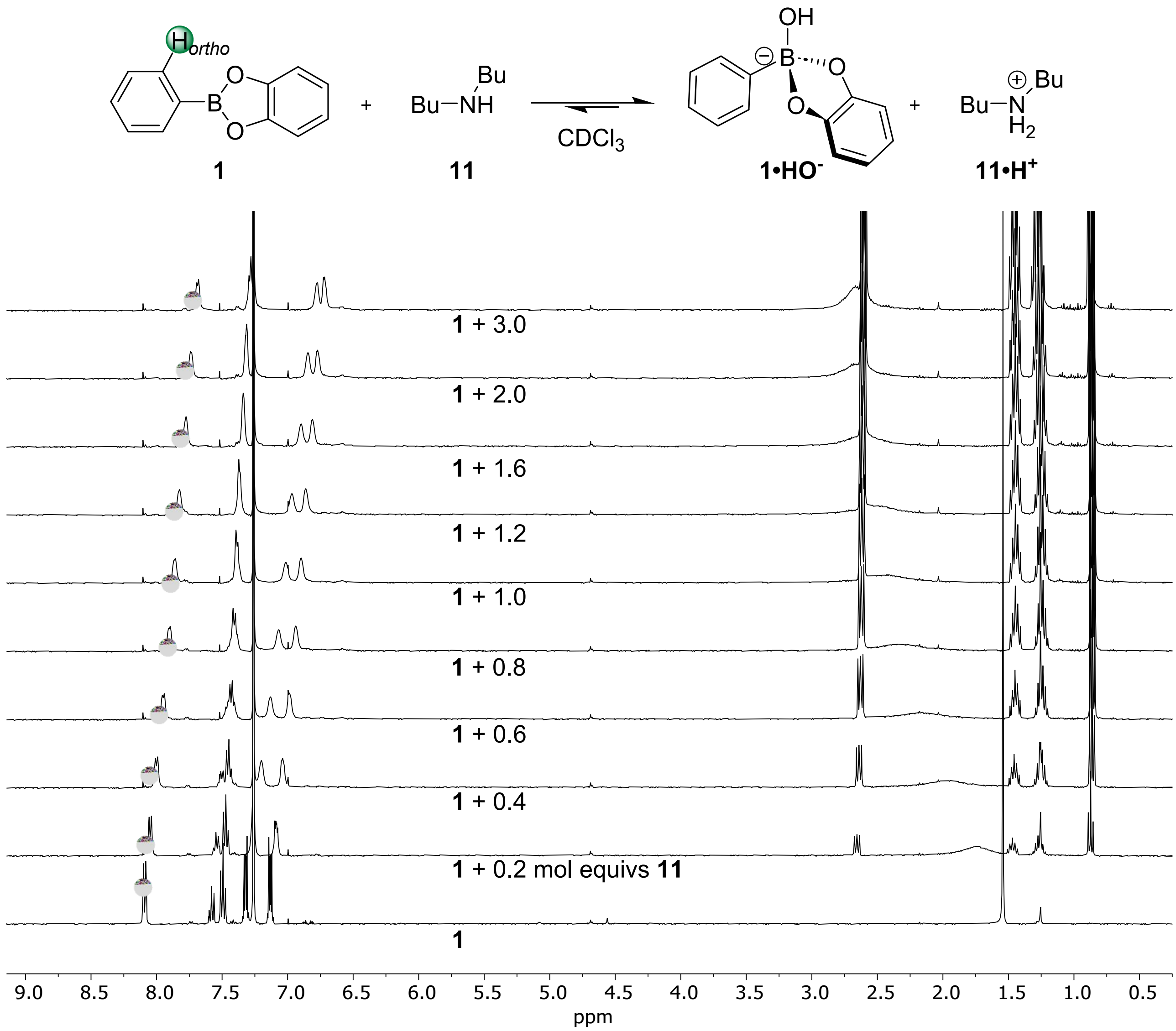

Figure S31. Top: reaction scheme for the ^{1}H NMR titration of **1** with dibutylamine (**11**). Bottom: spectra. The H_{ortho} proton signal is highlighted by a green sphere on the spectra.

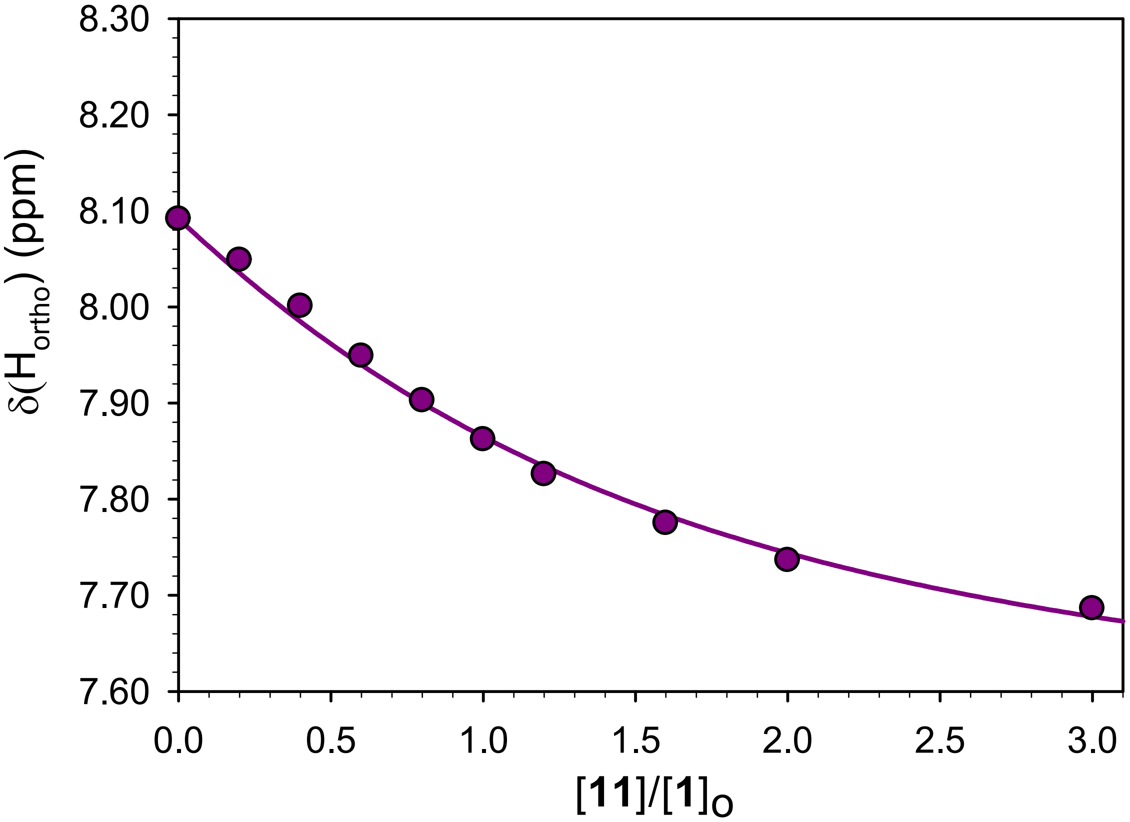

Figure S32. Chemical shift of the H_{ortho} proton on **1** upon addition of dibutylamine (**11**).

$$K_{obs} = 200\ M^{-1}$$

Extrapolated chemical shift at saturation δH_{ortho}: 7.51 ppm.

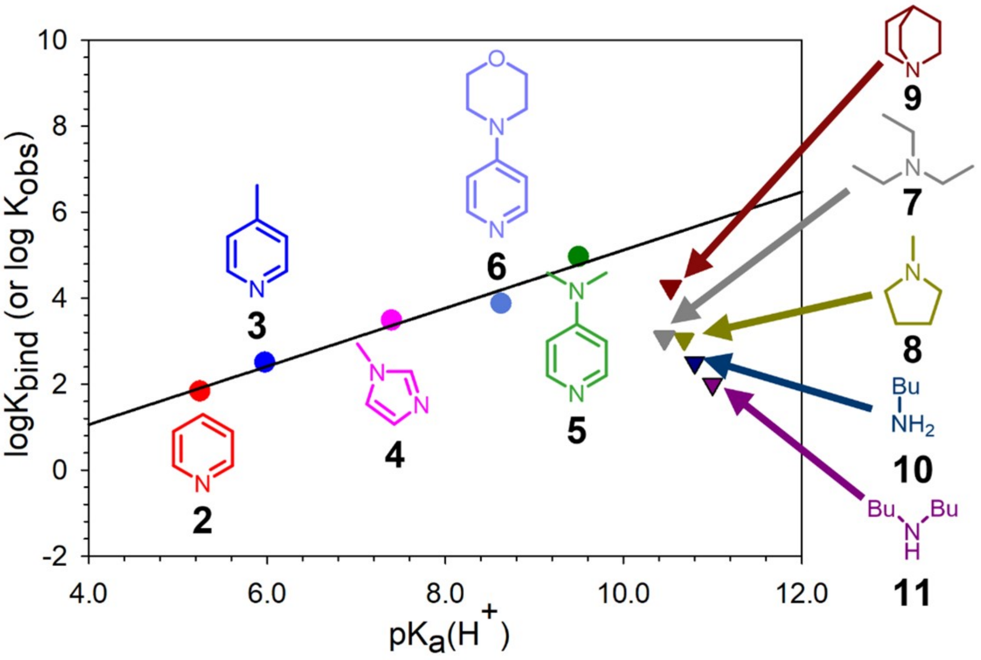

Figure S33. Linear correlation between the pK_aH^+ of the *N*ArHets **2-6** and the log K_{bind} for their adduct with **1**. The points relative to amines **7-11** are shown (log K_{obs} is considered), but their data is not used for the best-fit model.

$$\log K_{bind} = 0.68 \cdot pK_aH^+ - 1.65 \quad [R^2 = 0.97]$$

The values of pK_aH^+ for the aliphatic amines are taken from the literature. All values are measured in water, since quality data for the whole series of amines was not readily available in any other solvent. However, regardless of the solvent, for any given *N*ArHet the pK_aH^+ is a measurement of its basicity. The linear correlation between pK_aH^+ and $\log K_{bind}$ is therefore interpreted as a positive correlation between the Brønsted (affinity for the proton) and Lewis (affinity **1**) acidities for *N*ArHets **2-6.** Furthermore, the pK_aH^+ measured in water for compounds **2-11** is a lower bound for its value in inert, nonbasic solvents such as chloroform.[3]

Table S2. $pK_a(H^+)$ values for the bases employed in this work.

Base	*$pK_a(H^+)$*	*Reference*
Pyridine (**2**)	5.23	Ref. 4
4-methylpyridine (**3**)	5.99	Ref. 5
1-methylimidazole (**4**)	7.4	Ref. 6
Dimethyl(pyridin-4-yl)amine (**5**)	9.5	Ref. 7
4-(4-pyridyl)morpholine (**6**)	8.63	Ref. 8
Triethylamine (**7**)	10.68	Ref. 9
1-methylpyrrolidine (**8**)	10.46	Ref. 10
Quinuclidine (**9**)	10.53	Ref. 11
Butylamine (**10**)	10.77	Ref. 12
Dibutylamine (**11**)	11.1	Ref. 13

Effect of the bulkiness around the B atom.

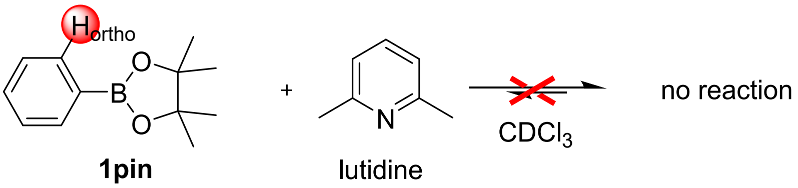

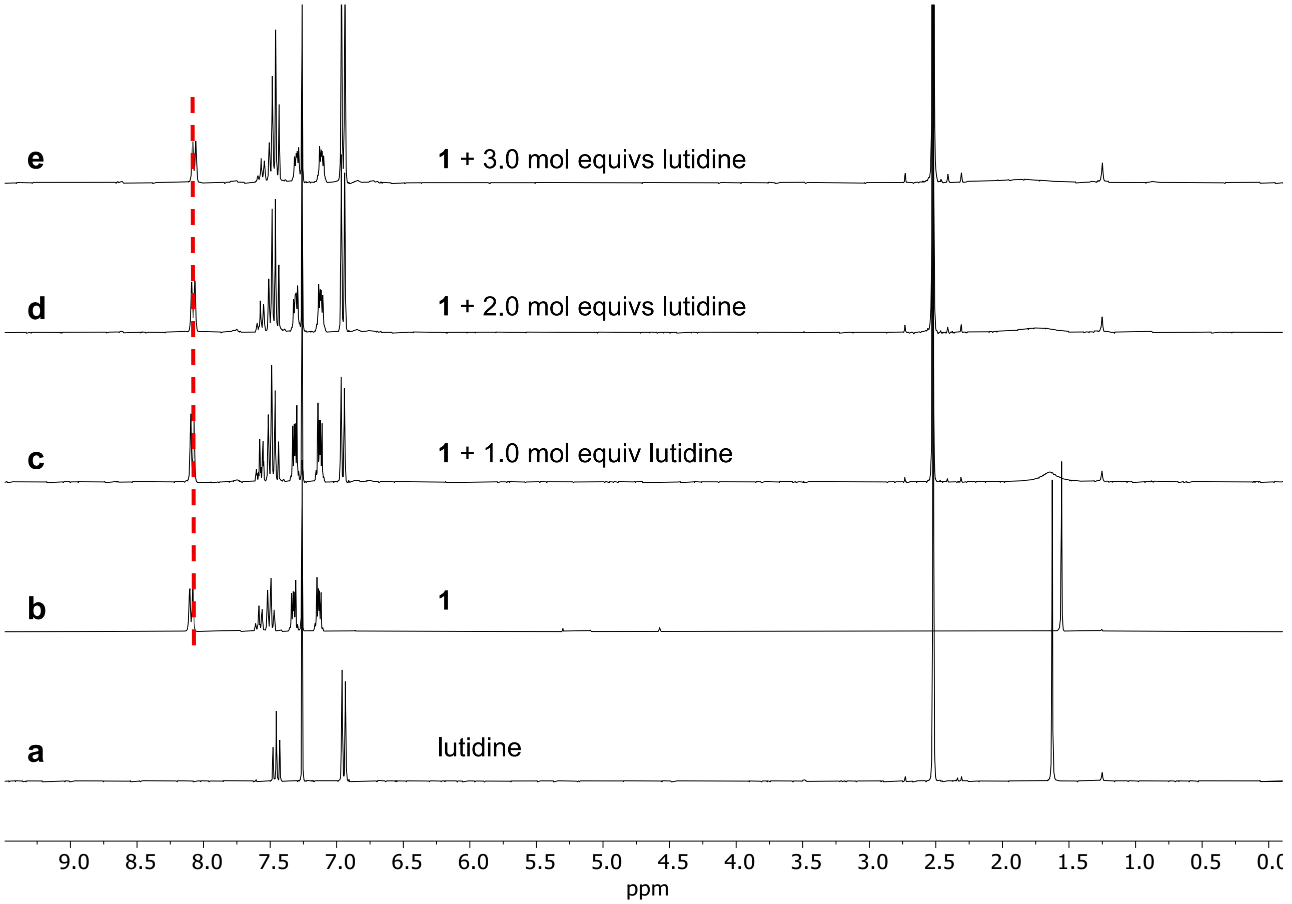

Figure S34. Addition of lutidine to a 15 mM solution of **1.** Trace a:15 mM lutidine; trace b 15 mM **1**; trace c: 15 mM **1** + 15 mM lutidine (1.0 mol equivs.); trace d: 15 mM **1** + 30 mM lutidine (2.0 mol equivs); trace d: 15 mM **1** + 30 mM lutidine (3 mol equivs). Spectra recorded at 25°C in $CDCl_3$. Full spectra. No significant shift of the H_{ortho} proton is observed, indicating very weak binding in spite the higher basicity of lutidine with respect to **3** (pK_aH^+(**3**) = 5.99[5], pK_aH^+(lutidine) =6.77[14])

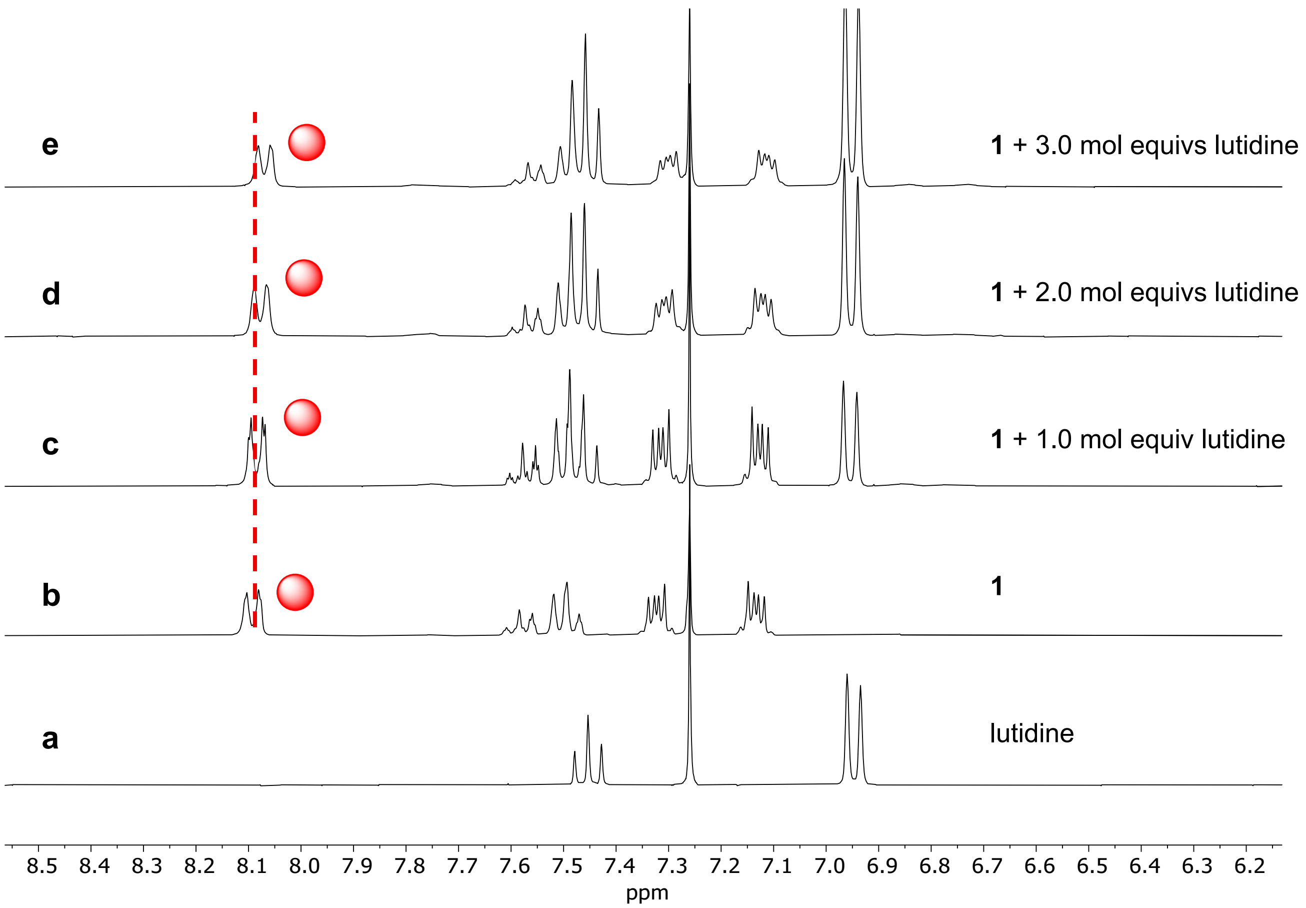

Figure S35. Addition of lutidine to a 15 mM solution of **1.** Trace a:15 mM lutidine; trace b 15 mM **1**; trace c: 15 mM **1** + 15 mM lutidine (1.0 mol equivs.); trace d: 15 mM **1** + 30 mM lutidine (2.0 mol equivs); trace d: 15 mM **1** + 30 mM lutidine (3 mol equivs). Spectra recorded at 25°C in $CDCl_3$. Zoom on the aromatic region. No significant shift of the H_{ortho} proton is observed, indicating very weak binding in spite the higher basicity of lutidine with respect to **3** (pK_aH^+(**3**) = 5.99[5], pK_aH^+(lutidine) =6.77[14]).

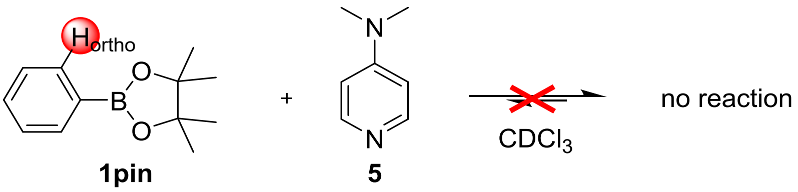

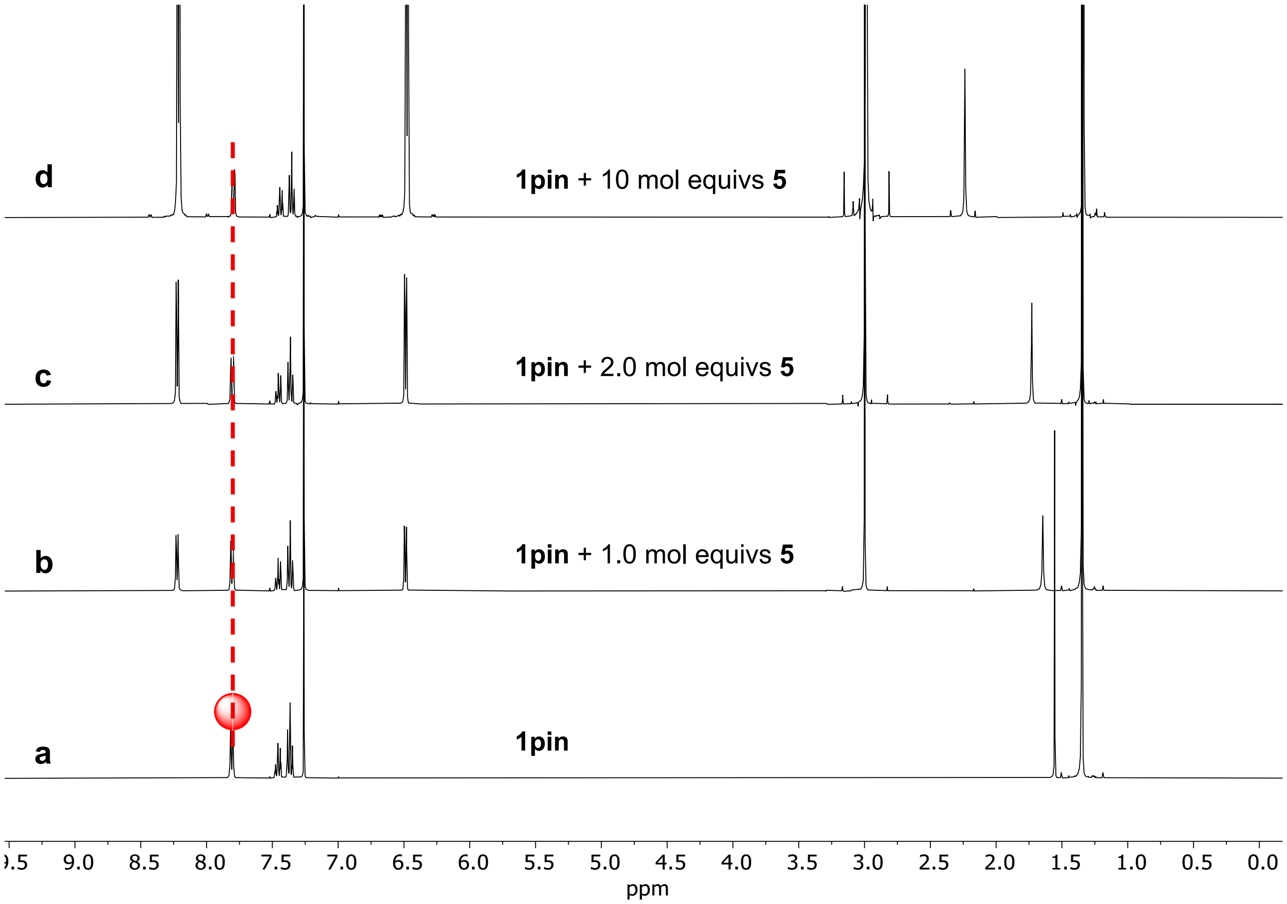

Figure S36. Addition of compound **5**, the strongest binder for **1** employed in this work, to a 10 mM solution of **1pin.** Trace a: 10 mM **1pin**; trace b: 10 mM **1pin** + 10 mM **5** (1.0 mol equivs.); trace c: 10 mM **1pin** + 20 mM **5** (2.0 mol equivs); trace c: 10 mM **1**pin + 100 mM **5** (10 mol equivs). Spectra recorded at 25°C in $CDCl_3$. Full spectra. Even adding a large excess of **5**, only a very slight shift of the H_{ortho} signal is observed indicating that if any binding occurs, it is very weak.

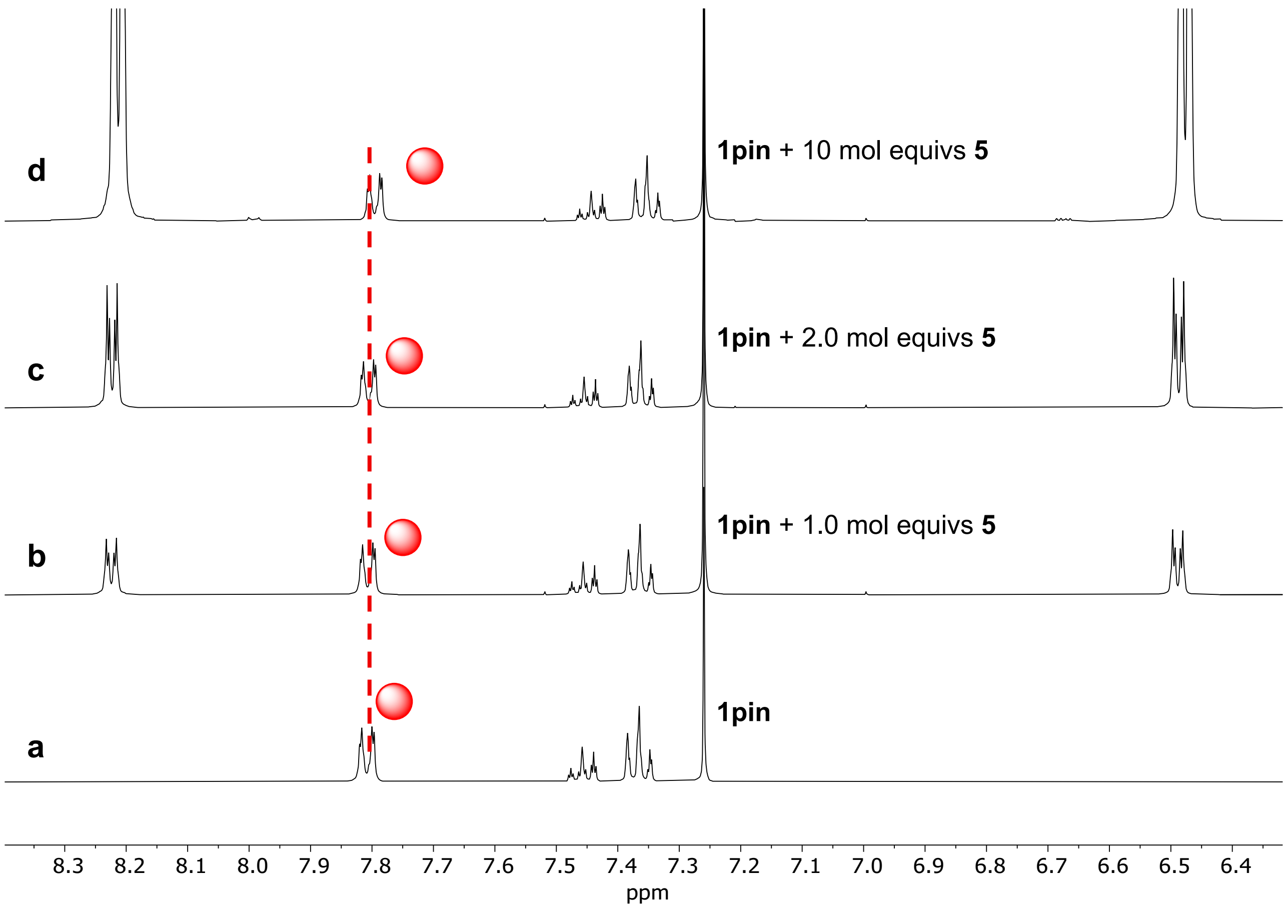

Figure S37. Addition of compound **5**, the strongest binder for **1** employed in this work, to a 10 mM solution of **1pin.** Trace a: 10 mM **1pin**; trace b: 10 mM **1pin** + 10 mM **5** (1.0 mol equivs.); trace c: 10 mM **1pin** + 20 mM **5** (2.0 mol equivs); trace c: 10 mM **1pin** + 100 mM **5** (10 mol equivs). Spectra recorded at 25°C in $CDCl_3$. Zoom on the aromatic region. Even adding a large excess of **5**, only a very slight shift of the H_{ortho} signal is observed indicating that if any binding occurs, it is very weak

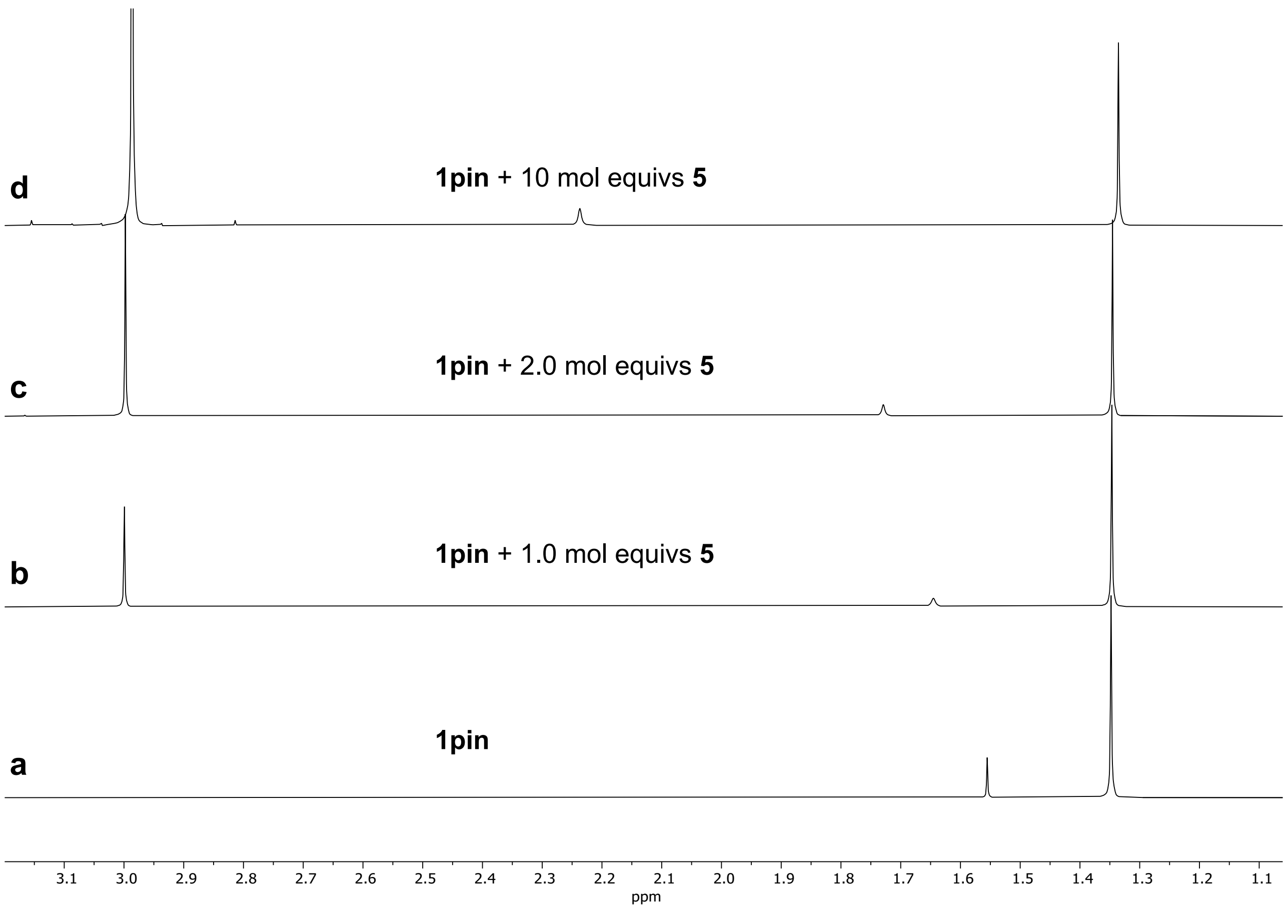

Figure S38. Addition of compound **5**, the strongest binder for **1** employed in this work, to a 10 mM solution of **1pin.** Trace a: 10 mM **1pin**; trace b: 10 mM **1pin** + 10 mM **5** (1.0 mol equivs.); trace c: 10 mM **1pin** + 20 mM **5** (2.0 mol equivs); trace c: 10 mM **1pin** + 100 mM **5** (10 mol equivs). Spectra recorded at 25°C in $CDCl_3$. Spectra recorded at 25°C. Zoom on the methyl region.

Selected example of speciation for the DL obtained from the combination of 1 with bases 2 and 3.

The percentage of bound pyridines and **1** in the DL obtained combining **1** with weak bases **2** and **3** (spectra reported in Figure 3 of the Main Text) was estimated as follows.

First, we found a relationship between the percentage of bound **1** and chemical shift of the H_{ortho} ($\delta(H_o)$ related to proton on the phenylboronic catechol ester moiety). We observed that, for the H_{ortho} proton, the saturation chemical shift extrapolated from the titration experiments did not depend on the choice of base used for the experiment and always turned out to be around 7.50 ppm for complexes **1•2**, **1•3**, **1•4** (outlier at 7.57 ppm), and **1•5**. On the other hand, proton H_{ortho} in free **1** resonates at 8.090 ppm.

We could therefore obtain the following equation:

$$\text{Eq. S1} \quad \%(bound\ 1) = 1371 - 169.5\delta(H_{ortho})$$

Then, we used the K_{bind} values to calculate the expected concentrations of complex when **1** (12.5 mM in $CDCl_3$) is placed in the presence of equimolar amounts of **2** or **3**, or both bases at the same time and we compared such concentrations with the ones calculated from Eq. S1 (see Table S3). We were pleased to note that the values obtained with the two methods were very similar, being within 2 percent points from each other.

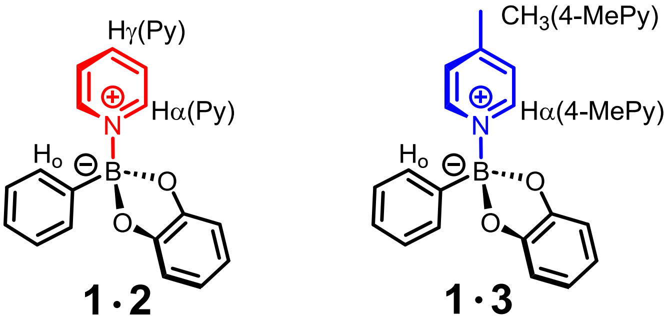

Figure S39. Structures of the complexes **1•2** and **1•3** with tags identifying the protons whose signal is followed in the NMR analyses.

Table S3. Estimation of the amount of bound **1** calculated from the chemical shift of the H_o proton or the K_{bind}. In each experiment, all the species are 12.5 mM in $CDCl_3$.

species in solution	$\delta(H_o)$	bound **1**[a]	bound **1**[b]
**1**+**2**	7.871 ppm	37%	35%
**1**+**3**	7.746 ppm	58%	60%
**1**+**2**+**3**	7.677 ppm	70%	71%[c]

[a] Calculated from Eq S1. [b] Calculated from K_{bind}. [c] ChemEQL was used to speed up the calculation.

By measuring the chemical shift of diagnostic signals of **2** and **3** in the presence and absence of **1** at known concentrations and correlating such data with the amount of free **2** and **3** obtained from the binding constants K_{bind}, we were able to obtain Equations S2-S5 which could be used to quantify the amount of bound **2** or **3** from the chemical shift of diagnostic signals (the gamma and alpha hydrogens for **2**, and the methyl and the alpha hydrogen for **3**). Choosing two signals for each pyridine provided redundancy. In all cases, the values obtained by the three methods were very close (see Table S4). We used equations S2- S5 to estimate the amount of free base(s) for the association experiments (**1** and **2** or **3**, all 12.5 mM in $CDCl_3$), as well as for the competition experiment (**1**, **2** and **3**, all 12.5 mM in $CDCl_3$).

Eq. S2 $$\%(bound\ 3) = 1517 - 645.2\delta(CH_3)$$

Eq. S3 $$\%(bound\ 3) = -3625 + 428.6\delta(H_\alpha)$$

Eq. S4 $$\%(bound\ 2) = -1853 + 241.3\delta(H_\gamma)$$

Eq. S5 $$\%(bound\ 2) = -3064 + 355.7\delta(H_\alpha)$$

Table S4. Estimation of the amount of bound **2** and **3** calculated from chemical shifts or from the K_{bind}. In each experiment, all the species are 12.5 mM in $CDCl_3$.

	$\delta(CH_3(\mathbf{3}))$	$\delta(H_\alpha(\mathbf{3}))$	$\delta(H_\gamma(\mathbf{2}))$	$\delta(H_\alpha(\mathbf{2}))$	bound **2**[a]	bound **2**[b]	bound **2**[c]	bound **3**[d]	bound **3**[e]	bound **3**[c]
**1+2**	--	--	7.823 ppm	8.714 ppm	35%	36%	37%	--	--	--
**1+3**	2.445 ppm	8.600 ppm	--	--	--	--	--	61%	61%	60%
**1+2+3**	2.429 ppm	8.576 ppm	7.759 ppm	8.669 ppm	19%	20%	19%	50%	51%	52%

[a]Calculated from Eq S2. [b]Calculated from Eq S3. [c]Calculated from K_{bind}. ChemEQL was used to speed up the calculation. [d]Calculated from Eq S4. [e]Calculated from Eq S5.

In the presence of both bases, we estimate that 20% of **2** is found in the bound form (complex **1•2**) while 80% is unbound. On the other hand, **3** is almost equally distributed between the bound (complex **1•3**) and the free form.

The amount of bound **1** in these conditions can be found by either summing the amount of bound **2** and **3**, or by Equation 1. In both cases, we estimate the bound forms of **1** to account for about 70% of the material (compare row 3 of Table S3 with row 3 of Table S4).

In all the cases, the figures obtained from K_{bind} values were in good accordance with the ones obtained from evaluating equations S2-S5 with the experimental chemical shifts.

We derived a simple method for estimating the composition of dynamic libraries generated from the combination of **1** and pyridines **2** and **3** with simple NMR experiments. This method could easily be extended to more complex cases with more than two competing bases, provided that at least one diagnostic signal can be individuated for each species.

Unfortunately, for stronger bases such as 4-dimethylaminopyridine (**5**) and triethylamine (**7**), equations analogous to those found for **2** and **3** yield unreliable figures, probably due to the partial protonation of such bases by the adventitious water both in the calibration and DL samples. Still, in all cases the amount of bound **1** can be estimated with Equation S1 which yields reproducible values in accordance with the computer-assisted calculations (more details in the discussion of the NMR competitive experiments, Figure S45 onwards).

Here below (Table S5) is reported the z file generated by ChemEQL for the competition experiment. All K values come from the above-reported titration experiments.

The software ChemEQL is freely available at https://github.com/eawag-surface-waters-research/ChemEQL.[15]

Table S5. Z file from ChemEQL. The bold titles and the notes in parenthesis are from the Authors and not part of the original file.

**Species**	**Stoich. Matrix**	**Log K**	**Conc. [mol/l]**	**Log conc.**
4MePy (**3**)	0 0 1	0.00E+00	6.01E-03 (48%)	-2.22E+00
PhBCat (**1**)	1 0 0	0.00E+00	3.58E-03 (29%)	-2.45E+00
PhBCat4MePy (**1•3**)	1 0 1	2.48E+00	6.49E-03 (52%)	-2.19E+00
PhBCatPy (**1•2**)	1 1 0	1.83E+00	2.43E-03 (19%)	-2.61E+00
Py (**2**)	0 1 0	0.00E+00	1.01E-02 (81%)	-2.00E+00
**Components**	**Mode**	**Initial Conc.**	**In or out of system**	
PhBCat (**1**)	total	1.25E-02	----	
Py (**2**)	total	1.25E-02	----	
4MePy (**3**)	total	1.25E-02	----	

Equation S1 is also used for mixtures where **4** is present. Due to the difference in saturation chemical shift, this leads to errors in the order of less than 10% which is acceptable, considering the simplicity of this approach.

Repeatability tests on the time-controlled, 12-CO_2H-enabled dissociation of 1•5

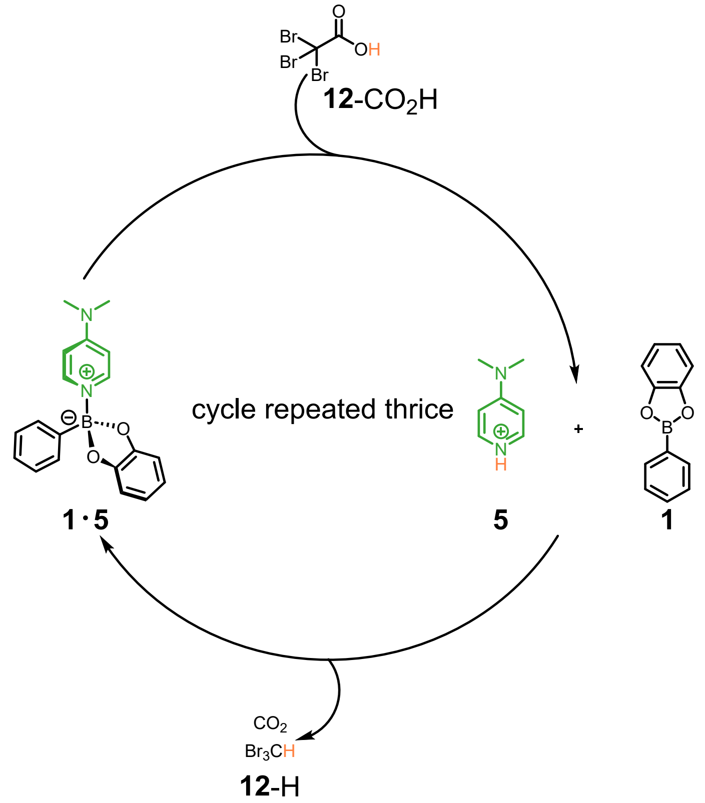

Figure S40. Reaction scheme for the **12**-CO_2H-enabled dissociation of complex **1•5** and its following, spontaneous reassociation upon decarboxylation of **12**-CO_2H.

1 and **5** (10 mM each in $CDCl_3$) were mixed in an NMR tube to furnish complex **1•5**. Upon addition of **12-**CO_2H the *in*-situ formed complex **1•5** underwent dissociation. Then, following the decarboxylation of **12-**CO_2H, the system gradually returned to its initial state. The cycle was repeated a total of three times by adding 1.0 mol equivalents of **12**-CO_2H at the end of the reaction.

Figures S41-S43 report the NMR spectra recorded over the course of the repeated addition experiment. Table S6 serves as a legend for such spectra.

The chemical shift of the H_o proton on **1** was followed in time. Employing Eq. S1, one can relate such chemical shift to the percentage of associated complex in solution. Such quantities are plotted against time in Figure S44.

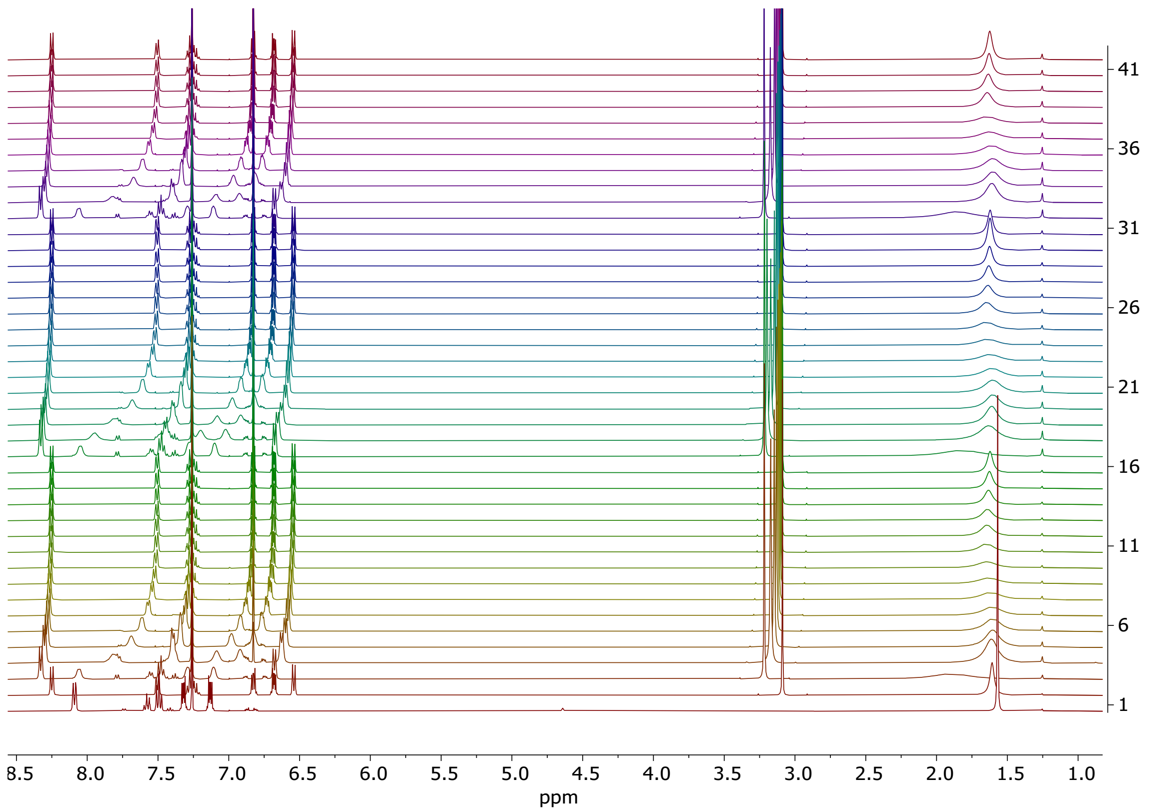

Figure S41. **6**-enabled dissociation of adduct **1•5** and its following, spontaneous reassociation upon decarboxylation of **12**-CO_2H. Full spectra. Legend reported in Table S6. Reaction followed at T = 21 °C.

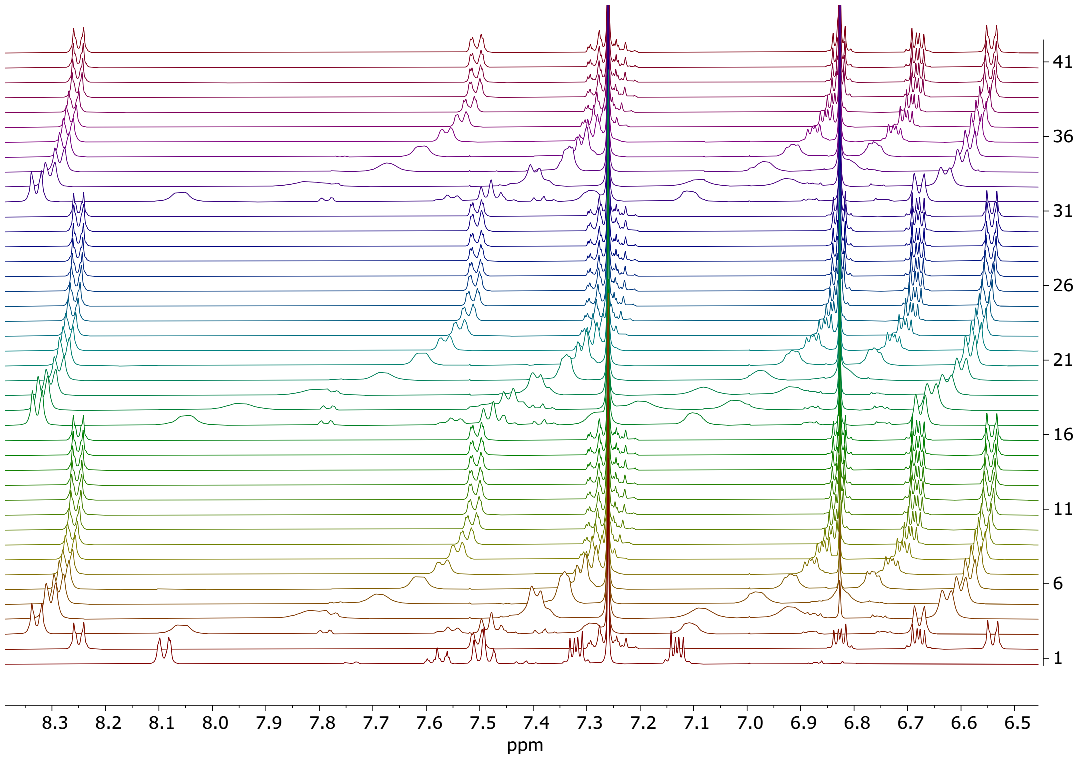

Figure S42. **6**-enabled dissociation of adduct **1•5** and its following, spontaneous reassociation upon decarboxylation of **12**-CO_2H. Zoom on the aromatics' region. Legend reported in Table S6. Reaction followed at T = 21 °C.

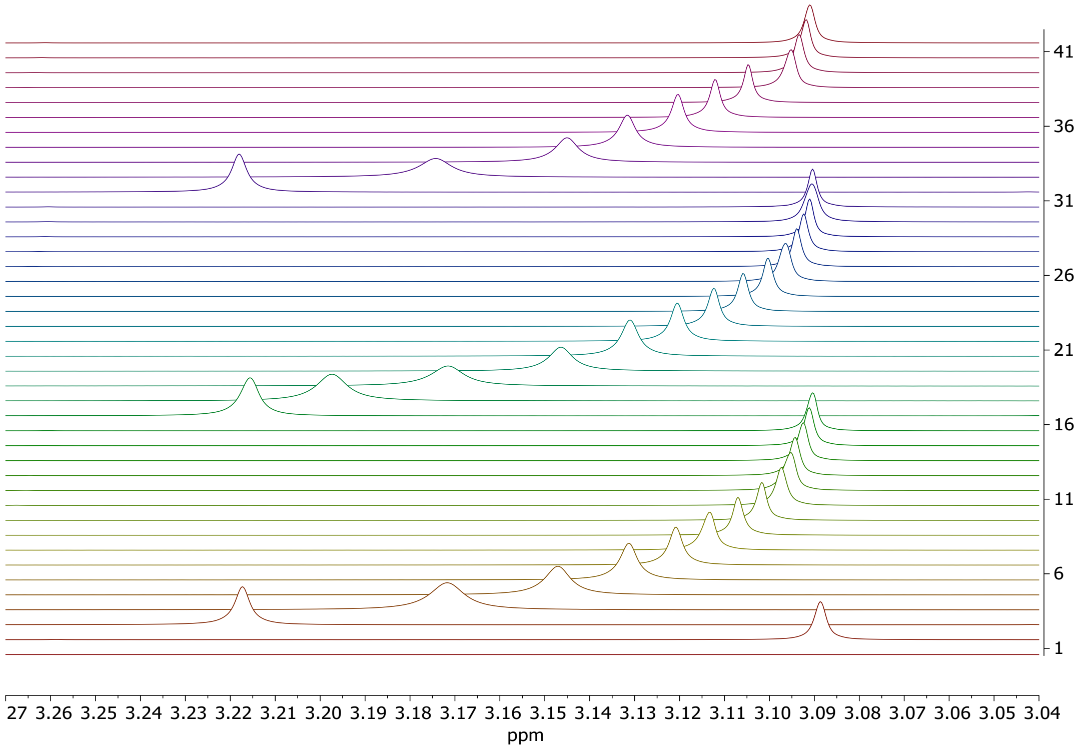

Figure S43. **6**-enabled dissociation of adduct **1•5** and its following, spontaneous reassociation upon decarboxylation of **12**-CO_2H. Zoom on the methyls' region. Legend reported in Table S6. Reaction followed at T = 21 °C.

Table S6. Legend for the spectra shown in Figures S41-S43.

Spectrum number	hh	mm	Notes
**1**			**1** (1.0 mol equivs.)
**2**			**1** and **5** (10 mM each)
**3**	0	0	Spectrum recorded after the first addition of **12**-CO_2H (1.0 mol equivs.)
**4**	6	4	
**5**	12	3	
**6**	18	3	
**7**	24	3	
**8**	30	4	
**9**	36	4	
**10**	42	4	
**11**	48	10	
**12**	51	28	
**13**	54	2	
**14**	60	4	
**15**	66	4	
**16**	73	30	
**17**	74	40	Spectrum recorded after the second addition of **12**-CO_2H (1.0 mol equivs.)
**18**	76	42	
**19**	80	42	
**20**	86	42	
**21**	92	42	
**22**	98	42	
**23**	104	40	
**24**	110	40	
**25**	116	40	
**26**	122	40	
**27**	128	40	
**28**	134	40	
**29**	140	40	
**30**	146	54	
**31**	147	24	
**32**	147	36	Spectrum recorded after the third addition of **12**-CO_2H (1.0 mol equivs)
**33**	153	33	
**34**	160	37	
**35**	165	41	
**36**	171	37	
**37**	177	34	
**38**	183	35	
**39**	196	5	
**40**	201	33	
**41**	207	36	
**42**	213	33	

**43**	219	33	

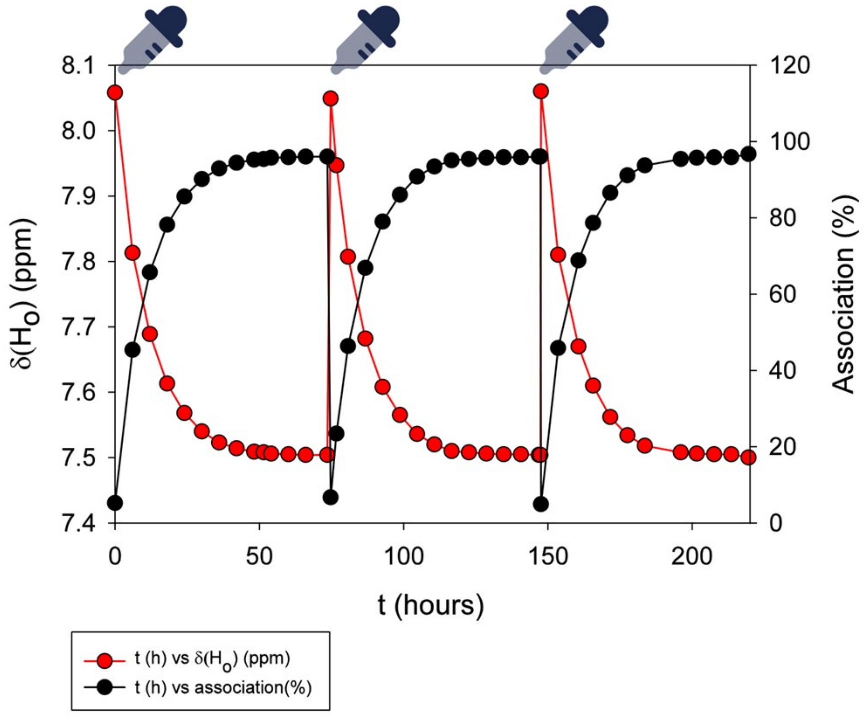

Figure S44. **12**-CO_2H-enabled dissociation of complex **1•5** and its following, spontaneous reassociation upon decarboxylation of **12**-CO_2H. Plot of the chemical shift of the H_o proton on **1**, as well as the percentage of associated complex over time calculated with Eq. S1. The droppers on the top represent the addition of ACA **12**-CO_2H to the system (1.0 mol equivalents with respect to **1** and **5**). Reaction followed at T = 21 °C.

NMR competitive experiments

Here follow the spectra of the competitive binding experiments. For each DL, we calculated the speciation with ChemEQL. The results are reported in the form of tables. For DLs containing only *N*ArHets, Eq. S1 (estimation of bound **1** from the chemical shift of the H_{ortho} proton or its complement to 100% to estimate free **1**) is used as a proxy to quickly validate the reliability of the calculation.

For DLs containing only aliphatic amines, the estimation provided by Eq. S1 does not agree with the results from the computer-calculated speciation since the saturation chemical shift of the H_{ortho} proton of **1** in the titrations of **1** with aliphatic amines turns out to be base-dependent (see the titration curves of **1** with amines **7-11**). Regardless, it remains true that the more upfield-shifted the H_{ortho} proton, the larger the amount of bound **1** in the system.

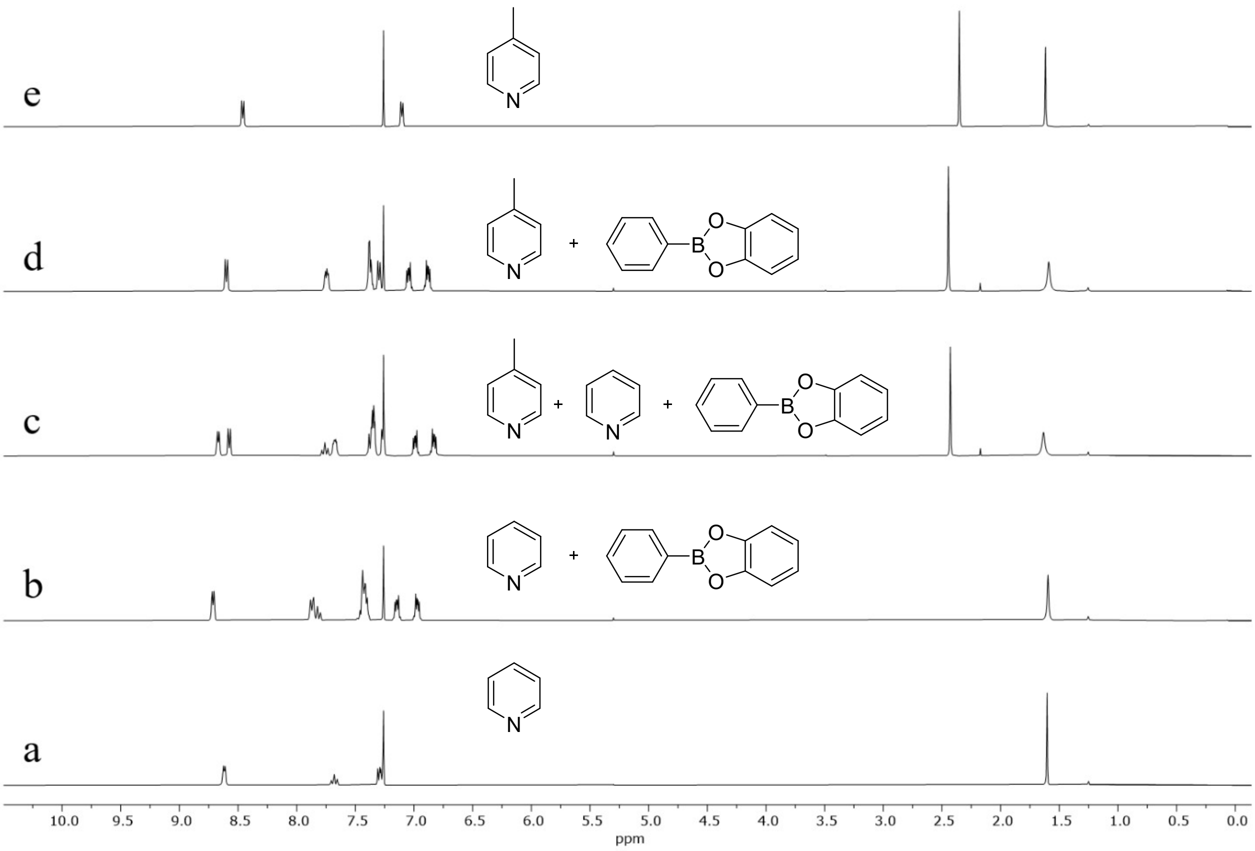

Figure S45. Exchange experiment in which pyridine **2** and 4-methyl pyridine **3** compete for phenylboronic acid catechol ester **1** ($CDCl_3$, 25°C). All the equilibria are fast on ^{1}H NMR time scale with adduct **1•3** prevailing on adduct **1•2** under competitive conditions (trace c). Trace a 12.5 mM **2**, trace b 12.5 mM **1** and **2**, trace c 12.5 mM **1**, **2** and **3**, trace d 12.5 mM **1** and **3**, trace e 12.5 mM **3**.

Table S7. Z file from ChemEQL for the DL shown in trace b of Figure S45 (12.5 mM **1** and **2** in $CDCl_3$, 25°C). The bold titles and the notes in parenthesis are from the Authors and not part of the original file. Figures followed by an asterisk are calculated from Eq. S1

**Species**	**Stoich. Matrix**	**Log K**	**Conc. [mol/l]**	**Log conc.**
PhBCat (**1**)	1 0	0.00E+00	8.08E-03 (65%, 63%*)	-2.09
PhBCatPy (**1•2**)	1 1	1.83E+00	4.42E-03 (35%, 37%*)	-2.36
Py (**2**)	0 1	0.00E+00	8.08E-03 (65%)	-2.09
**Components**	**Mode**	**Initial Conc.**	**In or out of system**	
PhBCat (**1**)	total	1.25E-02	----	
Py (**2**)	total	1.25E-02	----	

Table S8. Z file from ChemEQL for the DL shown in trace d of Figure S45 (12.5 mM **1** and **3** in $CDCl_3$, 25°C). The bold titles and the notes in parenthesis are from the Authors and not part of the original file. Figures followed by an asterisk are calculated from Eq. S1

**Species**	**Stoich. Matrix**	**Log K**	**Conc. [mol/l]**	**Log conc.**
4MePy (**3**)	0 1	0.00E+00	4.99E-03 (40%)	-2.30
PhBCat (**1**)	1 0	0.00E+00	4.99E-03 (40%, 42%*)	-2.30
PhBCat4MePy (**1•3**)	1 1	2.48E+00	7.51E-03 (60%, 58%*)	-2.12
**Components**	**Mode**	**Initial Conc.**	**In or out of system**	
PhBCat (**1**)	total	1.25E-02	----	
4MePy (**3**)	total	1.25E-02	----	

Table S9. Z file from ChemEQL for the DL shown in trace c of Figure S45 (12.5 mM **1**, **2** and **3** in $CDCl_3$, 25°C). The bold titles and the notes in parenthesis are from the Authors and not part of the original file. Figures followed by an asterisk are calculated from Eq. S1

**Species**	**Stoich. Matrix**	**Log K**	**Conc. [mol/l]**	**Log conc.**
4MePy (**3**)	0 0 1	0.00E+00	6.01E-03 (48%)	-2.22
PhBCat (**1**)	1 0 0	0.00E+00	3.58E-03 (29%, 30%*)	-2.45
PhBCat4MePy (**1•3**)	1 0 1	2.48E+00	6.49E-03 (52%)	-2.19
PhBCatPy (**1•2**)	1 1 0	1.83E+00	2.43E-03 (19%)	-2.61
Py (**2**)	0 1 0	0.00E+00	1.01E-02 (81%)	-2.00
**Components**	**Mode**	**Initial Conc.**	**In or out of system**	
PhBCat (**1**)	total	1.25E-02	----	
Py (**2**)	total	1.25E-02	----	
4MePy (**3**)	total	1.25E-02	----	

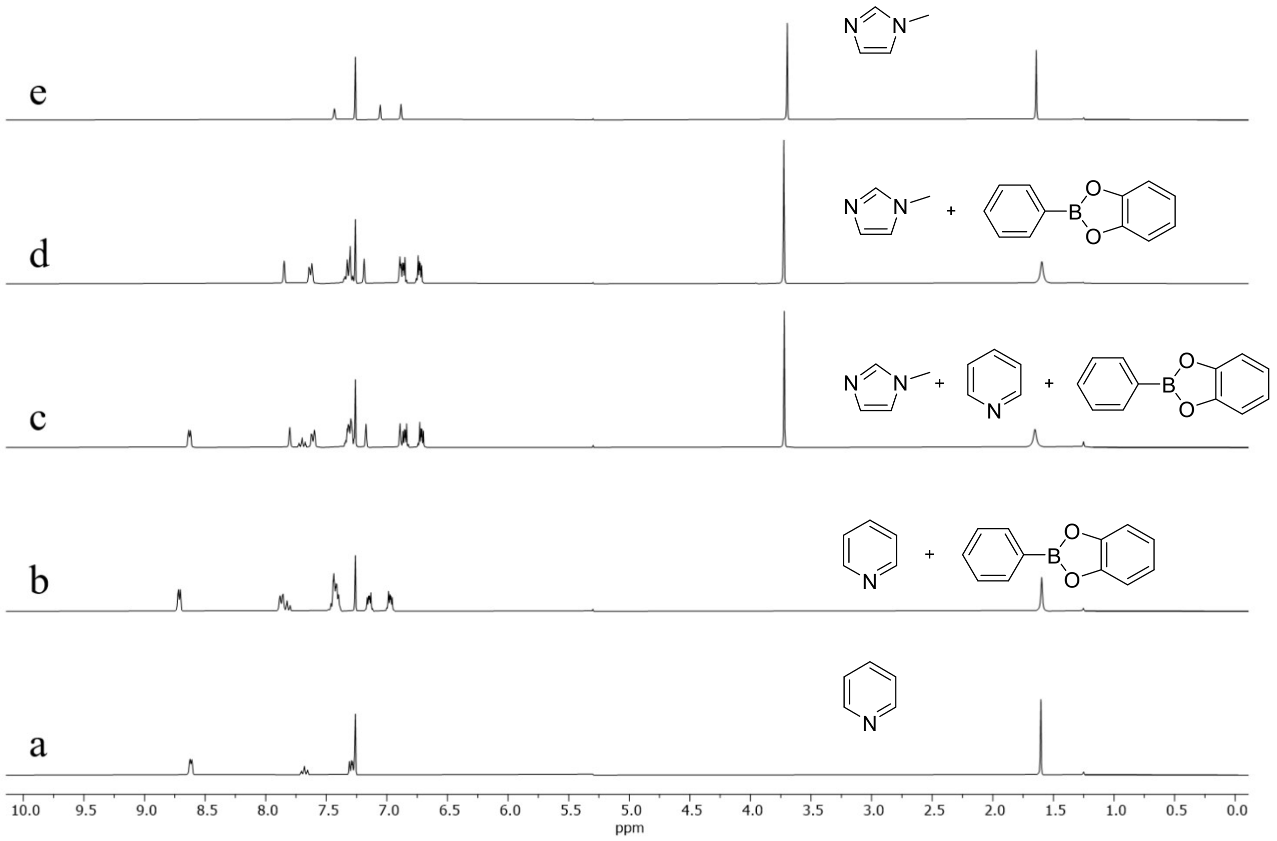

Figure S46. Exchange experiment in which pyridine **2** and 1-methylimidazole **4** compete for phenylboronic acid catechol ester **1** ($CDCl_3$, 25°C). All the equilibria are fast on ^{1}H NMR time scale with adduct **1•4** prevailing on adduct **1•2** under competitive conditions (trace c). Trace a 12.5 mM **2**, trace b 12.5 mM **1** and **2**, trace c 12.5 mM **1**, **2** and **4**, trace d 12.5 mM **1** and **4**, trace e 12.5 mM **4**.

Trace b: See Table S7.

Table S10. Z file from ChemEQL for the DL shown in trace d of Figure S46 (12.5 mM **1** and **4** in $CDCl_3$, 25°C). The bold titles and the notes in parenthesis are from the Authors and not part of the original file. Figures followed by an asterisk are calculated from Eq. S1.

**Species**	**Stoich. Matrix**	**Log K**	**Conc. [mol/l]**	**Log conc.**
4-MeImidazole (**4**)	0 1	0.00E+00	1.88E-03 (15%)	-2.73
PhBCat (**1**)	1 0	0.00E+00	1.88E-03 (15%, 22%*)	-2.73
PhBCat4-MeImidazole (**1•4**)	1 1	3.48E+00	1.06E-02 (85%, 78%*)	-1.97
**Components**	**Mode**	**Initial Conc.**	**In or out of system**	
PhBCat (**1**)	total	1.25E-02	----	
4-MeImidazole (**4**)	total	1.25E-02	----	

Table S11. Z file from ChemEQL for the DL shown in trace c of Figure S46 (12.5 mM **1**, **2** and **4** in $CDCl_3$, 25°C). The bold titles and the notes in parenthesis are from the Authors and not part of the original file. Figures followed by an asterisk are calculated from Eq. S1.

**Species**	**Stoich. Matrix**	**Log K**	**Conc. [mol/l]**	**Log conc.**
4-MeImidazole (**4**)	0 1 0	0.00E+00	2.43E-03 (19%)	-2.61
PhBCat (**1**)	1 0 0	0.00E+00	1.37E-03 (11%, 19%*)	-2.86
PhBCat4-MeImidazole (**1•4**)	1 1 0	3.48E+00	1.01E-02 (81%)	-2.00
PhBCatPy (**1•2**)	1 0 1	1.83E+00	1.06E-03 (9%)	-2.98
Py (**2**)	0 0 1	0.00E+00	1.14E-02 (91%)	-1.94
**Components**	**Mode**	**Initial Conc.**	**In or out of system**	
PhBCat (**1**)	total	1.25E-02	----	
4-MeImidazole (**4**)	total	1.25E-02	----	
Py (**2**)	total	1.25E-02	----	

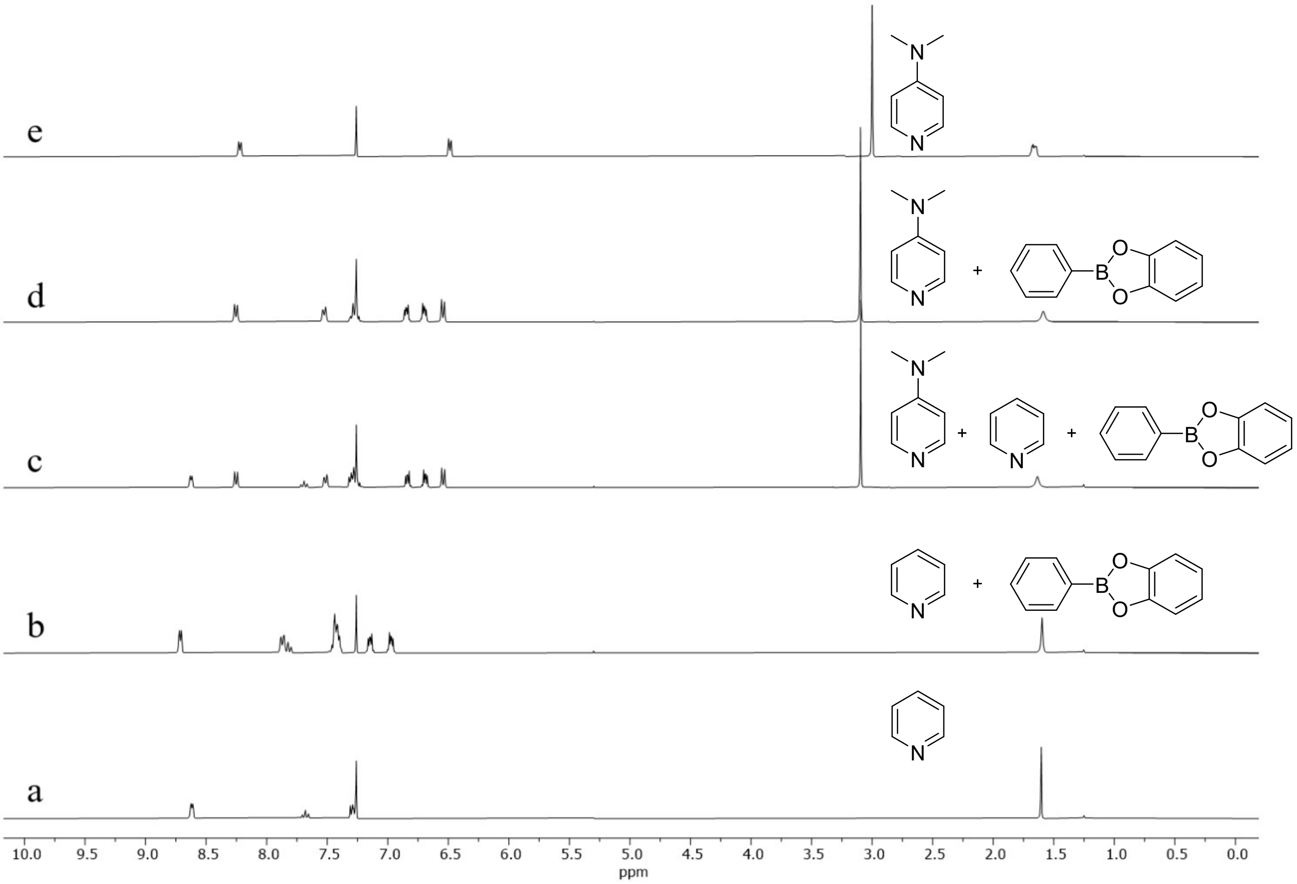

Figure S47. Exchange experiment in which pyridine **2** and 4-(dimethylamino)pyridine **5** compete for phenylboronic acid catechol ester **1** ($CDCl_3$, 25°C). All the equilibria are fast on ^{1}H NMR time scale with adduct **1•5** prevailing on adduct **1•2** under competitive conditions (trace c). Trace a 12.5 mM **2**, trace b 12.5 mM **1** and **2**, trace c 12.5 mM **1**, **2** and **5**, trace d 12.5 mM **1** and **5**, trace e 12.5 mM **5**.

Trace b: See Table S7.

Table S12. Z file from ChemEQL for the DL shown in trace c of Figure S47 (12.5 mM **1, 2** and **5** in $CDCl_3$, 25°C). The bold titles and the notes in parenthesis are from the Authors and not part of the original file. Figures followed by an asterisk are calculated from Eq. S1.

**Species**	**Stoich. Matrix**	**Log K**	**Conc. [mol/l]**	**Log conc.**
DMAP (**5**)	0 1 0	0.00E+00	4.91E-04 (4%)	-3.31
PhBCat (**1**)	1 0 0	0.00E+00	2.68E-04 (2%, 2%*)	-3.57
PhBCatDMAP (**1•5**)	1 1 0	4.96E+00	1.20E-02 (96%)	-1.92
PhBCatPy (**1•2**)	1 0 1	1.83E+00	2.23E-04 (2%)	-3.65
Py (**2**)	0 0 1	0.00E+00	1.23E-02 (98%)	-1.91
**Components**	**Mode**	**Initial Conc.**	**In or out of system**	
PhBCat (**1**)	total	1.25E-02	----	
DMAP (**5**)	total	1.25E-02	----	
Py (**2**)	total	1.25E-02	----	

Table S13. Z file from ChemEQL for the DL shown in trace d of Figure S47 (12.5 mM **1** and **5** in $CDCl_3$, 25°C). The bold titles and the notes in parenthesis are from the Authors and not part of the original file. Figures followed by an asterisk are calculated from Eq. S1.

**Species**	**Stoich. Matrix**	**Log K**	**Conc. [mol/l]**	**Log conc.**
DMAP (**5**)	0 1	0.00E+00	3.65E-04 (3%)	-3.44
PhBCat (**1**)	1 0	0.00E+00	3.65E-04 (3%, 4%*)	-3.44
PhBCatDMAP (**1•5**)	1 1	4.96E+00	1.21E-02 (97%, 96%*)	-1.92
**Components**	**Mode**	**Initial Conc.**	**In or out of system**	
PhBCat (**1**)	total	1.25E-02	----	
DMAP (**5**)	total	1.25E-02	----	

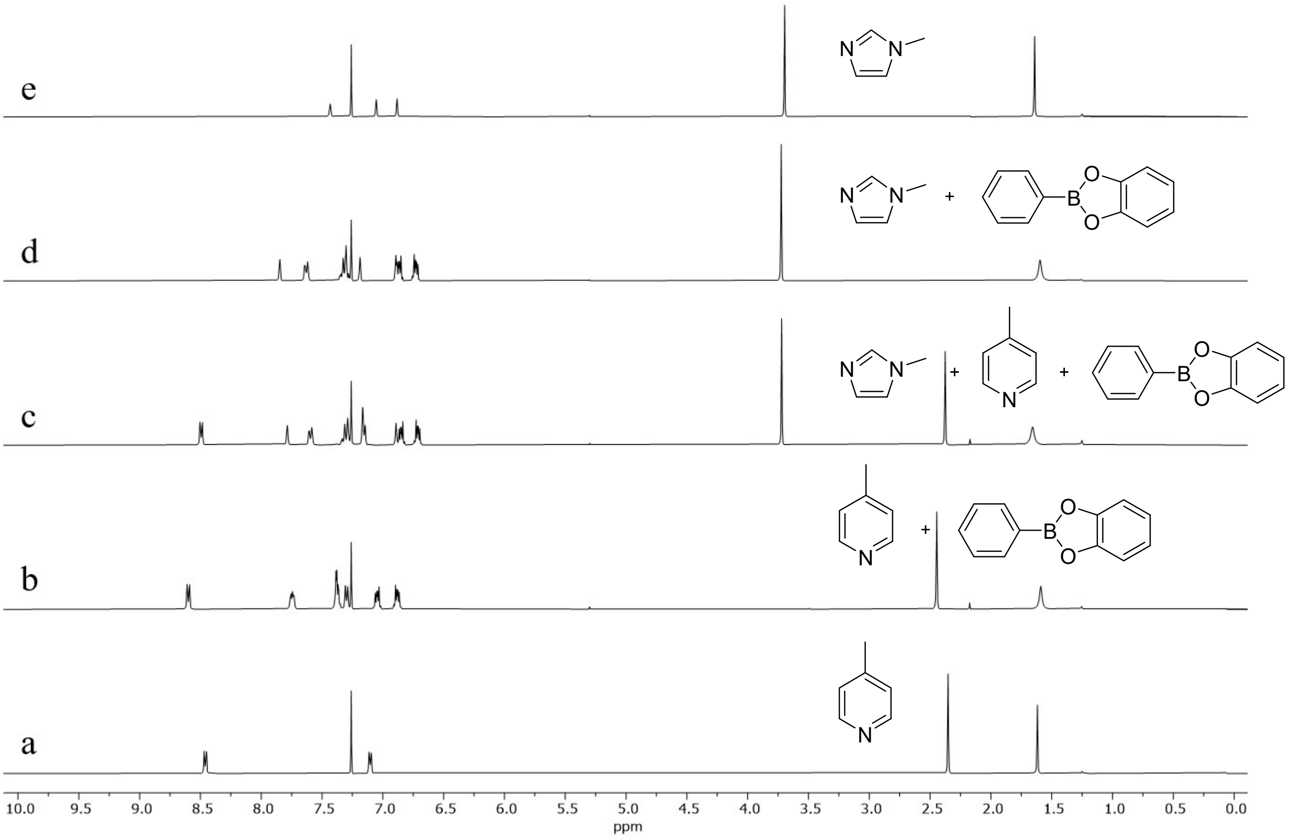

Figure S48. Exchange experiment in which 4-methyl pyridine **3** and 1-methylimidazole **4** compete for phenylboronic acid catechol ester **1** ($CDCl_3$, 25°C). All the equilibria are fast on ^{1}H NMR time scale with adduct **1•4** prevailing on adduct **1•3** under competitive conditions (trace c). Trace a 12.5 mM **3**, trace b 12.5 mM **1** and **3**, trace c 12.5 mM **1**, **3** and **4**, trace d 12.5 mM **1** and **4**, trace e 12.5 mM **4**.

Trace b: See Table S8.

Table S14. Z file from ChemEQL for the DL shown in trace c of Figure S48 (12.5 mM **1, 3** and **4** in $CDCl_3$, 25°C). The bold titles and the notes in parenthesis are from the Authors and not part of the original file. Figures followed by an asterisk are calculated from Eq. S1.

**Species**	**Stoich. Matrix**	**Log K**	**Conc. [mol/l]**	**Log conc.**
4-MeImidazole (**4**)	0 1 0	0.00E+00	3.46E-03 (28%)	-2.46
4MePy (**3**)	0 0 1	0.00E+00	9.91E-03 (79%)	-2.00
PhBCat (**1**)	1 0 0	0.00E+00	8.66E-04 (7%, 16%*)	-3.06
PhBCat4-MeImidazole (**1•4**)	1 1 0	3.48E+00	9.04E-03 (72%)	-2.04
PhBCat4MePy (**1•3**)	1 0 1	2.48E+00	2.59E-03 (21%)	-2.59
**Components**	**Mode**	**Initial Conc.**	**In or out of system**	
PhBCat (**1**)	total	1.25E-02	----	
4-MeImidazole (**4**)	total	1.25E-02	----	
4MePy (**3**)	total	1.25E-02	----	

Trace d: See Table S10

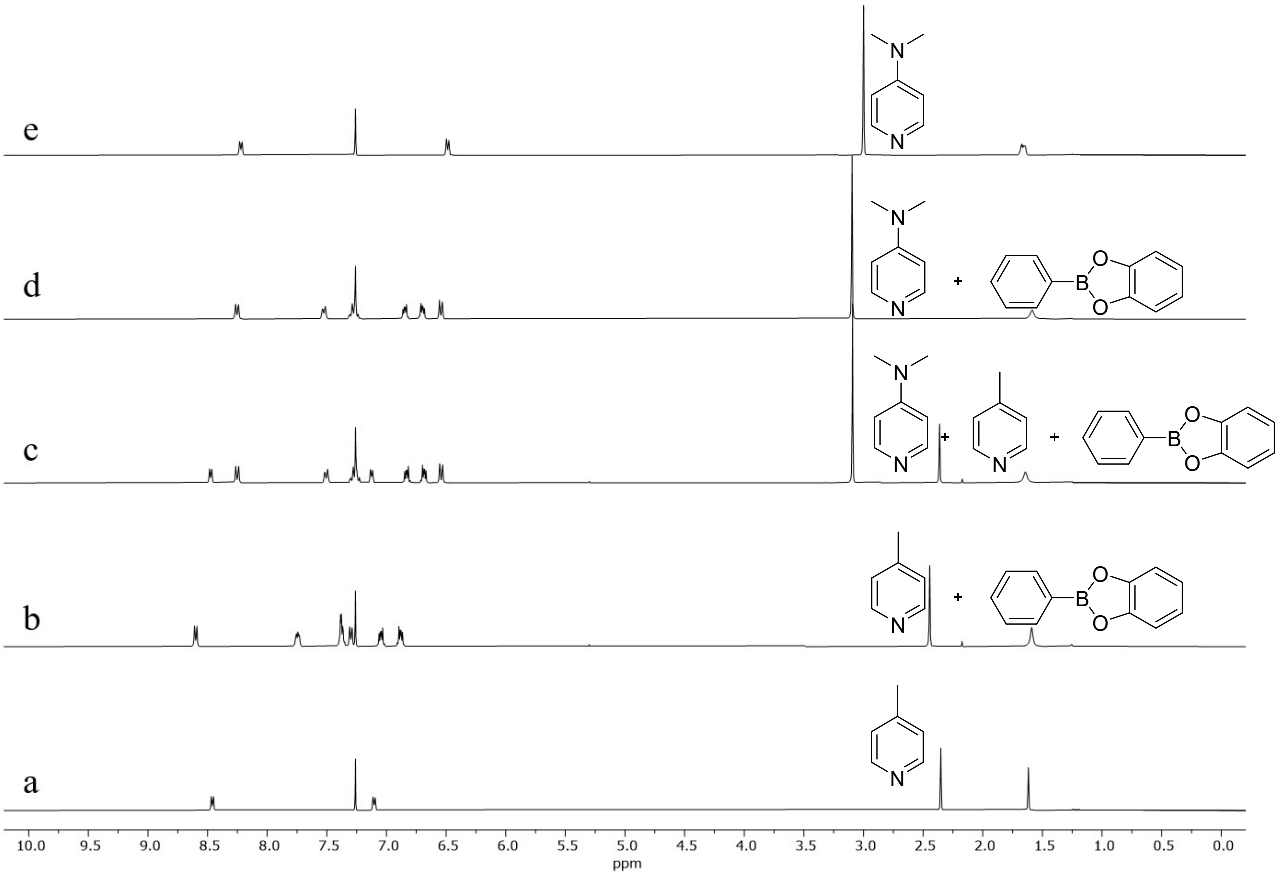

Figure S49. Exchange experiment in which 4-methyl pyridine **3** and 4-(dimethylamino)pyridine **5** compete for phenylboronic acid catechol ester **1** ($CDCl_3$, 25°C). All the equilibria are fast on ^{1}H NMR time scale with adduct **1•5** prevailing on adduct **1•3** under competitive conditions (trace c). Trace a 12.5 mM **3**, trace b 12.5 mM **1** and **3**, trace c 12.5 mM **1**, **3** and **5**, trace d 12.5 mM **1** and **5**, trace e 12.5 mM **5**.

Trace b: See Table S8.

Table S15. Z file from ChemEQL for the DL shown in trace c of Figure S49 (12.5 mM **1, 3** and **5** in $CDCl_3$, 25°C). The bold titles and the notes in parenthesis are from the Authors and not part of the original file. Figures followed by an asterisk are calculated from Eq. S1.

**Species**	**Stoich. Matrix**	**Log K**	**Conc. [mol/l]**	**Log conc.**
4MePy (**3**)	0 0 1	0.00E+00	1.19E-02 (90%)	-1.93
DMAP (**5**)	0 1 0	0.00E+00	7.69E-04 (6%)	-3.11
PhBCat (**1**)	1 0 0	0.00E+00	1.67E-04 (1%, <1%*)	-3.78
PhBCat4MePy (**1•3**)	1 0 1	2.48E+00	6.01E-04 (5%)	-3.22
PhBCatDMAP (**1•5**)	1 1 0	4.96E+00	1.17E-02 (94%)	-1.93
**Components**	**Mode**	**Initial Conc.**	**In or out of system**	
PhBCat (**1**)	total	1.25E-02	----	
DMAP (**5**)	total	1.25E-02	----	
4MePy (**3**)	total	1.25E-02	----	

Trace d: See Table S13.

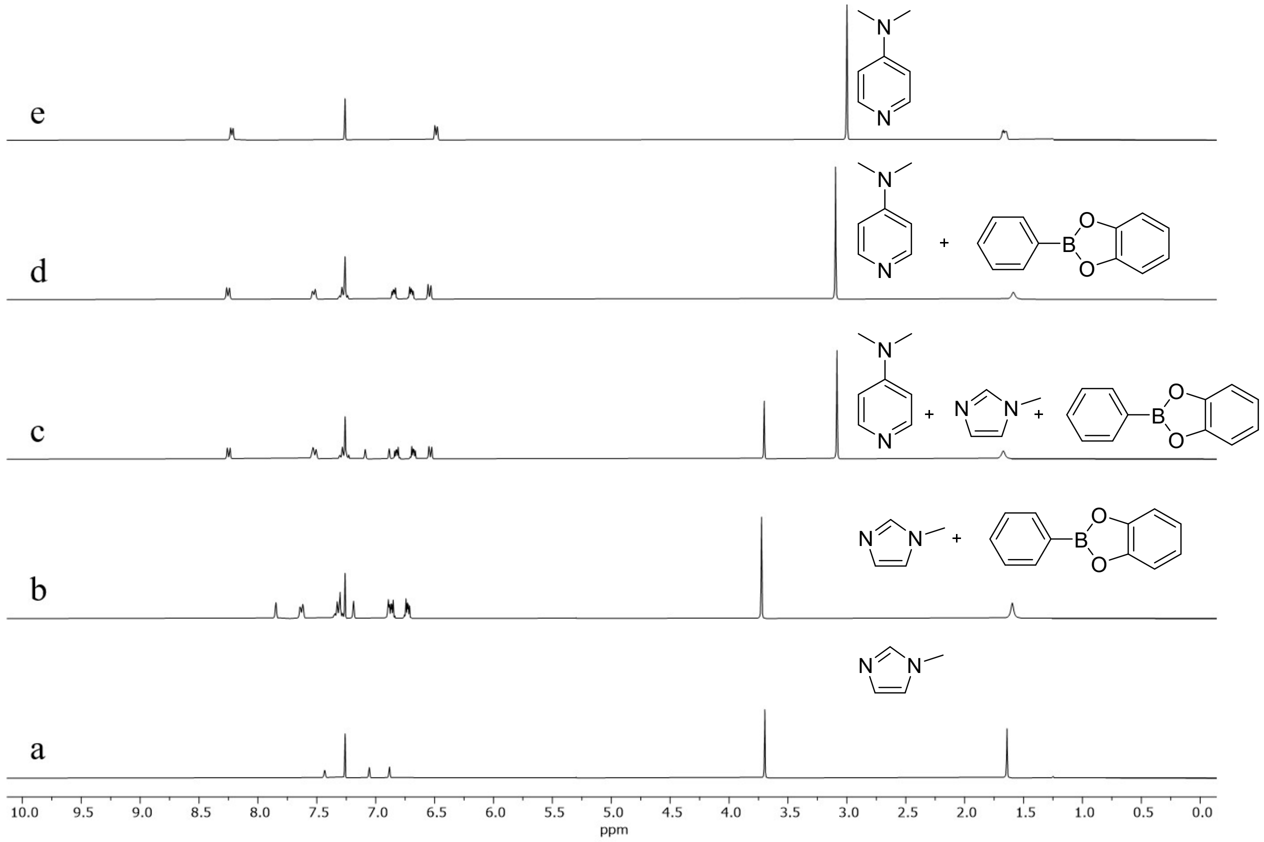

Figure S50. Exchange experiment in which 1-methylimidazole **4** and 4-(dimethylamino)pyridine **5** compete for phenylboronic acid catechol ester **1** ($CDCl_3$, 25°C). All the equilibria are fast on ^1H NMR time scale with adduct **1•5** prevailing on adduct **1•4** under competitive conditions (trace c). Trace a 12.5 mM **4**, trace b 12.5 mM **1** and **4**, trace c 12.5 mM **1**, **4** and **5**, trace d 12.5 mM **1** and **5**, trace e 12.5 mM **5**.

Trace b: See Table S10

Table S16. Z file from ChemEQL for the DL shown in trace c of Figure S50 (12.5 mM **1**, **4** and **5** in $CDCl_3$, 25°C). The bold titles and the notes in parenthesis are from the Authors and not part of the original file. Figures followed by an asterisk are calculated from Eq. S1.

**Species**	**Stoich. Matrix**	**Log K**	**Conc. [mol/l]**	**Log conc.**
4-MeImidazole (**4**)	0 1 0	0.00E+00	1.06E-02 (85%)	-1.97
DMAP (**5**)	0 0 1	0.00E+00	1.95E-03 (16%)	-2.71
PhBCat (**1**)	1 0 0	0.00E+00	5.92E-05 (<1%, <1%*)	-4.23
PhBCat4-MeImidazole (**1•4**)	1 1 0	3.48E+00	1.90E-03 (15%)	-2.72
PhBCatDMAP (**1•5**)	1 0 1	4.96E+00	1.06E-02 (85%)	-1.98
**Components**	**Mode**	**Initial Conc.**	**In or out of system**	
PhBCat (**1**)	total	1.25E-02	----	
4-MeImidazole (**3**)	total	1.25E-02	----	
DMAP (**5**)	total	1.25E-02	----	

Trace d: See Table S13.

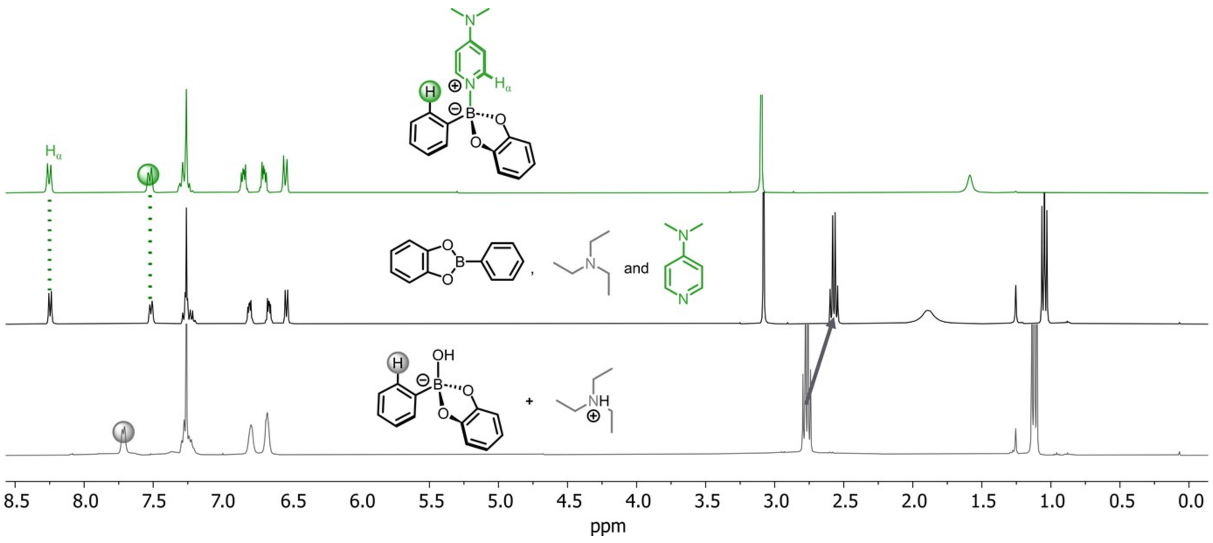

Figure S51. Bottom trace: **1** and **7**, both 12.5 mM; middle trace: **1**, **7,** and **5** (all 12.5 mM); top: **1** and **5** (both 12.5 mM). All the spectra are recorded at 25 ºC in $CDCl_3$. It is evident that, when both **7** and **5** are present in solution, protons H_{ortho} and Hα (belonging to **1** and **5** respectively) have very similar chemical shifts as they do when only **1** and **5** are present in solution (no addition of triethylamine, compare middle and top trace). Moreover, the addition of **5** causes all the signals related to **1** to become resolved (compare bottom and middle trace) and that the signals related to triethylamine are shifted upfield, indicating partial deprotonation.

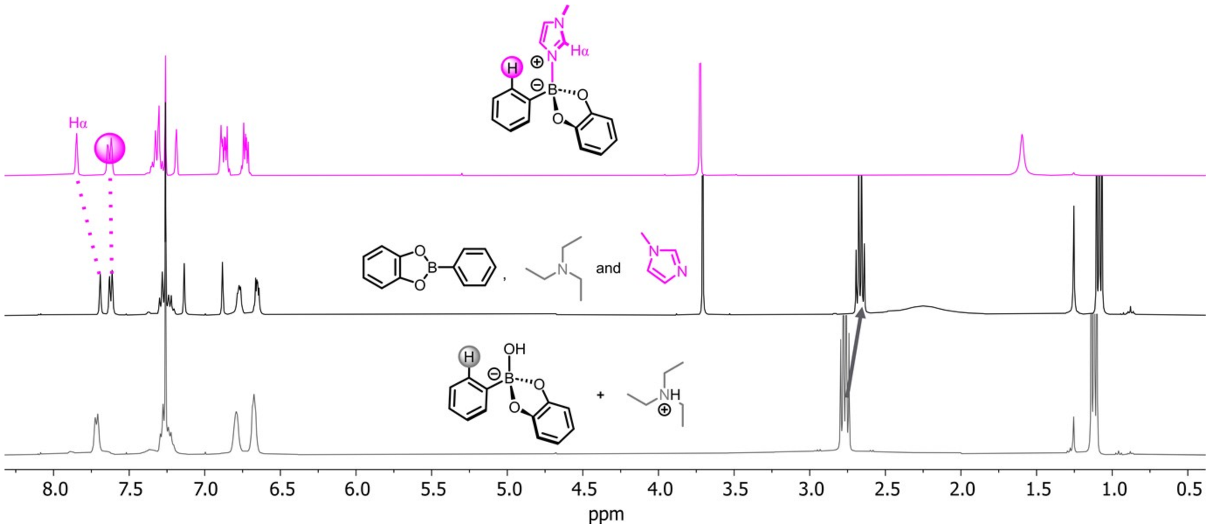

Figure S52. Bottom trace: **1** and **7**, both 12.5 mM; middle trace: **1**, **7,** and **4** (all 12.5 mM); top: **1** and **4** (both 12.5 mM). All the spectra are recorded at 25 ºC in $CDCl_3$. It is evident that, when both **7** and **4** are present in solution, protons H_{ortho} and Hα (belonging to **1** and **4** respectively) have relatively similar chemical shifts as they do when only **1** and **4** are present in solution (no addition of triethylamine, compare middle and top trace). More evidently, the addition of **4** causes all the signals related to **1** to become resolved (compare bottom and middle trace) and that the signals related to triethylamine are shifted upfield, indicating partial deprotonation.

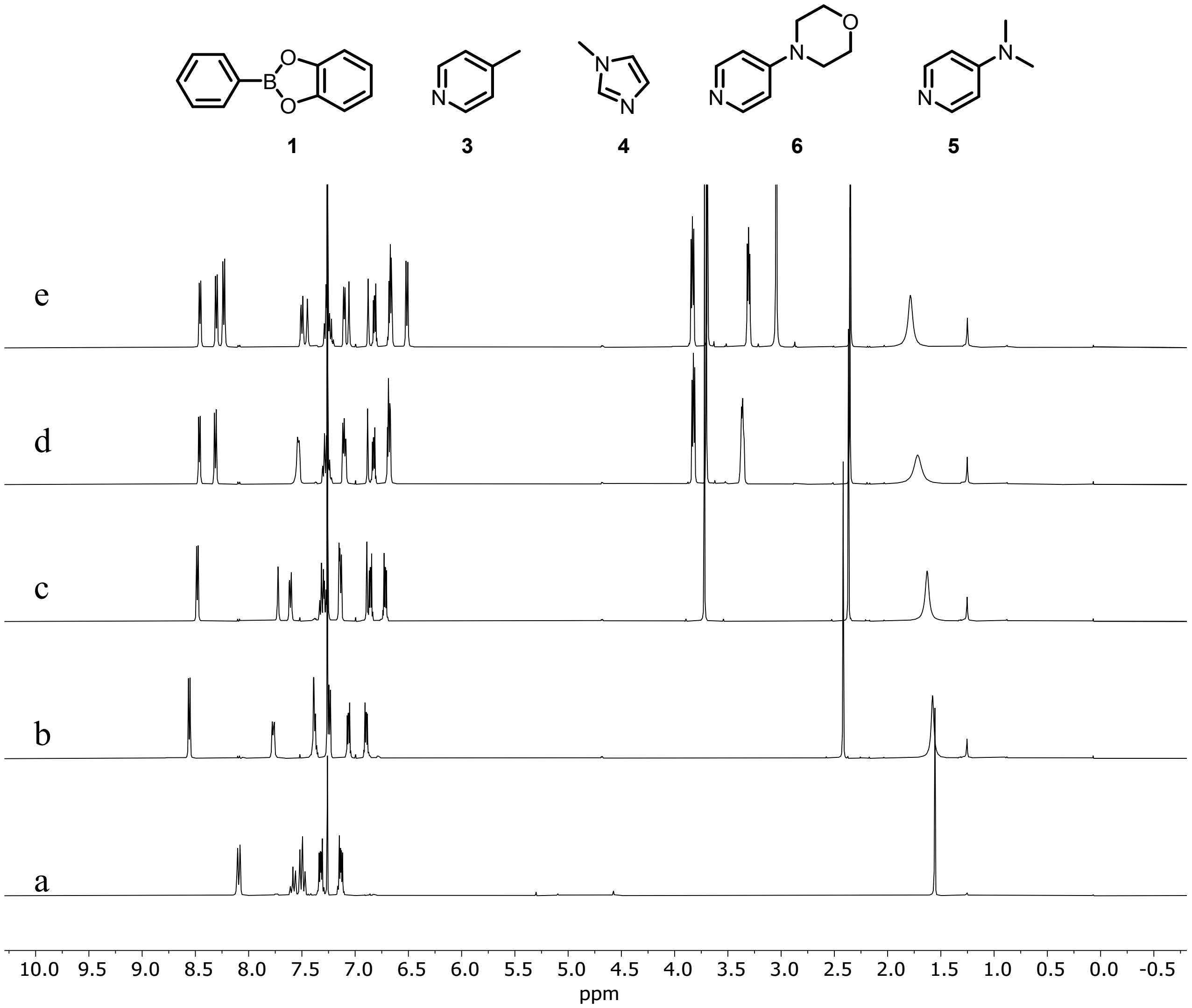

Figure S53. Generation of multiple DLs by consecutive addition of increasingly stronger binders for **1**. Top: compounds employed for the experiment (in order of addition and increasing affinity for **1**). Bottom: spectra. All the spectra are recorded in $CDCl_3$ at 25 °C Trace a: 12.5 mM **1**; trace b: 12.5 mM **1** and 12.5 mM **3**; trace c: 12.5 mM **1**, 12.5 mM **3**, and 12.5 mM **4**; trace d: 12.5 mM **1**, 12.5 mM **3**, 12.5 mM **4**, and 12.5 mM **6**; trace d: 12.5 mM **1**, 12.5 mM **3**, 12.5 mM **4**, 12.5 mM **6,** and 12.5 mM **5**.

Table S17. Z file from ChemEQL for the system whose spectrum is reported in trace b of Figure S53. The bold titles and the notes in parenthesis are from the Authors and not part of the original file. Values marked with an asterisk are calculated using Equation S1.

**Species**	**Stoich. Matrix**	**Log K**	**Conc. [mol/l]**	**Log conc.**
4MePy (**3**)	1 0	0.00E+00	4.90E-03 (39%)	-2.31
PhBCa (**1**)t	0 1	0.00E+00	4.90E-03 (39%, 44%*)	-2.31
PhBCat4MePy (**1•3**)	1 1	2.50E+00	7.60E-03 (61%)	-2.12
**Components**	**Mode**	**Initial Conc.**	**In or out of system**	
4MePy (**3**)	total	1.25E-02	----	
PhBCat (**1**)	total	1.25E-02	----	

Table S18. Z file from ChemEQL for the system whose spectrum is reported in trace c of Figure S53. The bold titles and the notes in parenthesis are from the Authors and not part of the original file. Values marked with an asterisk are calculated using Equation S1.

**Species**	**Stoich. Matrix**	**Log K**	**Conc. [mol/l]**	**Log conc.**
1-MeIm (**4**)	1 0 0	0.00E+00	3.44E-03 (28%)	-2.46
4MePy (**3**)	0 1 0	0.00E+00	9.89E-03 (79%)	-2.01
PhBCat (**1**)	0 0 1	0.00E+00	8.33E-04 (7%,15%*)	-3.08
PhBCat1MeIm (**1•4**)	1 0 1	3.50E+00	9.06E-03 (72%)	-2.04
PhBCat4MePy (**1•3**)	0 1 1	2.50E+00	2.61E-03 (21%)	-2.58
**Components**	**Mode**	**Initial Conc.**	**In or out of system**	
1-MeIm (**4**)	total	1.25E-02	----	
4MePy (**3**)	total	1.25E-02	----	
PhBCa (**1**)t	total	1.25E-02	----	

Table S19. Z file from ChemEQL for the system whose spectrum is reported in trace d of Figure S53. The bold titles and the notes in parenthesis are from the Authors and not part of the original file. Values marked with an asterisk are calculated using Equation S1.

**Species**	**Stoich. Matrix**	**Log K**	**Conc. [mol/l]**	**Log conc.**
1-MeIm (**4**)	0 1 0 0	0.00E+00	7.98E-03 (64%)	-2.098
4MePy (**3**)	0 0 1 0	0.00E+00	1.18E-02 (95%)	-1.927
DMAP_Morph (**6**)	1 0 0 0	0.00E+00	5.37E-03 (43%)	-2.27
PhBCat (**1**)	0 0 0 1	0.00E+00	1.79E-04 (1%, 4%*)	-3.747
PhBCat1MeIm (**1•4**)	0 1 0 1	3.50E+00	4.52E-03 (36%)	-2.345
PhBCat4MePy (**1•3**)	0 0 1 1	2.50E+00	6.70E-04 (5%)	-3.174
PhBCatDMAP_Morph (**1•6**)	1 0 0 1	3.87E+00	7.13E-03 (57%)	-2.147
**Components**	**Mode**	**Initial Conc.**	**In or out of system**	
DMAP_Morph (**6**)	total	1.25E-02	----	
1-MeIm (**4**)	total	1.25E-02	----	
4MePy (**3**)	total	1.25E-02	----	
PhBCat (**1**)	total	1.25E-02	----	

Table S20. Z file from ChemEQL for the system whose spectrum is reported in trace e of Figure S53. The bold titles and the notes in parenthesis are from the Authors and not part of the original file. Values marked with an asterisk are calculated using Equation S1.

**Species**	**Stoich. Matrix**	**Log K**	**Conc. [mol/l]**	**Log conc.**
1-MeIm (**4**)	0 0 1 0 0	0.00E+00	1.15E-02 (92%)	-1.94
4MePy (**3**)	0 0 0 1 0	0.00E+00	1.24E-02 (>99%)	-1.91
DMAP (**5**)	1 0 0 0 0	0.00E+00	3.30E-03 (26%)	-2.48
DMAP_Morph (**6**)	0 1 0 0 0	0.00E+00	1.04E-02	-1.99
PhBCat (**1**)	0 0 0 0 1	0.00E+00	2.79E-05 (<1%, <1%*)	-4.55
PhBCat1MeIm (**1•4**)	0 0 1 0 1	3.50E+00	1.01E-03 (8%)	-2.99
PhBCat4MePy (**1•3**)	0 0 0 1 1	2.50E+00	1.09E-04 (<1%)	-3.96
PhBCatDMAP (**1•5**)	1 0 0 0 1	5.00E+00	9.20E-03 (74%)	-2.04
PhBCatDMAP_Morph (**1•6**)	0 1 0 0 1	3.87E+00	2.14E-03 (17%)	-2.67
**Components**	**Mode**	**Initial Conc.**	**In or out of system**	
DMAP (**5**)	total	1.25E-02	----	
DMAP_Morph (**6**)	total	1.25E-02	----	
1-MeIm (**4**)	total	1.25E-02	----	
4MePy (**3**)	total	1.25E-02	----	
PhBCat (**1**)	total	1.25E-02	----	

Transiently controlling the composition of a DL generated from 1, 3 and 5 with ACA 12-CO_2H

Trace a of Figure 5 (Manuscript) corresponds to the equilibrated DL obtained combining **1**, **3**, and **5** in solution at 15.0 mM concentration ($CDCl_3$, 25 °C). The reaction scheme is shown in the top left quadrant of Figure 5 (Manuscript).
Applying Eq. S1, one can see that approximately 100% of **1** is bound to pyridines (both **1** and **3**. $\delta(H_o)$ = 7.50 ppm). From Eqs S2 and S3, we confirmed that **3** is mostly found in its unbound state ($\delta(CH_3)$ = 2.364 which, entered in Eq. S2, yields 8% bound **3**; $\delta(H_\alpha)$ = 8.491 which, entered in Eq. S3, yields 14% bound **3**).
Computer-assisted calculations on the speciation of the DL based on the K_{bind} values found for the complexes via ^1H NMR titration were performed employing the free software ChemEQL. The output is reported in Table S21.
The calculations suggest that **1** is mostly found in its bound state (99%). 5% of **3** is bound to **1** while 94% of **5** is found as complex **1•5**. Therefore, the ratio $\frac{[1•5]}{[1•3]}$ and that the ratio is 19.

Table S21. Z file from ChemEQL for the system in its initial state. The bold titles and the notes in parenthesis are from the Authors and not part of the original file.

**Species**	**Stoich. Matrix**	**Log K**	**Conc. [mol/l]**	**Log conc.**
4MePy (**3**)	0 0 1	0.00E+00	1.43E-02 (95%)	-1.85
DMAP (**5**)	0 1 0	0.00E+00	9.06E-04 (6%)	-3.04
PhBCat (**1**)	1 0 0	0.00E+00	1.71E-04 (1%)	-3.77
PhBCat4MePy (**1•3**)	1 0 1	2.48E+00	7.35E-04 (5%)	-3.13
PhBCatDMAP (**1•5**)	1 1 0	4.96E+00	1.41E-02 (94%)	-1.85
**Components**	**Mode**	**Initial Conc.**	**In or out of system**	
PhBCat (**1**)	total	1.50E-02	----	
DMAP (**5**)	total	1.50E-02	----	
4MePy (**3**)	total	1.50E-02	----	

Trace b of Figure 5 (Manuscript)corresponds to the DL obtained combining **1**, **3**, and **5** in solution at 15.0 mM concentration ($CDCl_3$, 25 °C) after the addition of **12**-CO_2H (15 mM in $CDCl_3$).
Applying Eq. S1, one can see that approximately 64% of **1** is bound to pyridines (both **1** and **3**. $\delta(H_o)$ = 7.713 ppm).
Since both protonation and complexation to **1** changes the chemical shifts of the diagnostic signals in the pyridines, Eqs S2-S4 cannot be used to determine the percentages pyridines in their bound state. However, in the presence of protonable pyridines, the signals of **1** are assumed not to be shifted significantly by the acid, so Eq. S1 is employable. Note that this assumption is only made when the acid is added in defect with respect to the total amount of pyridines.
Instead, computer-assisted calculations employing both the K_{bind} found via ^1H NMR titrations and the pK_aH^+ values of the pyridines were performed employing the free software ChemEQL. The output is reported in Table S22.
In order to simplify the system, **12**-CO_2H is treated as a strong acid. It is expected that, in the presence of 1 mol equivalents of two pyridines, only a negligible amount of **12-**CO_2H will be present in its undissociated state.
The calculations suggest that 69% of **1** is bound to pyridines (to be compared with 64% from Eq. S1).
5 is mostly found in its protonated state (74%) while the rest of it is bound to **1** (26%). **3** is present in its free form (31%), in its protonated state (26%), and bound to **1** (43%). Therefore, the ratio $\frac{[1•5]}{[1•3]}$ is 0.60.

Table S22. Z file from ChemEQL for the system in which 1 mol equiv **3** and 1 mol equiv **5** compete for 1 mol equiv of a strong acid. The bold titles and the notes in parenthesis are from the Authors and not part of the original file. H^+ (org) is a placeholder for any acid that is expected to be fully deprotonated in the reaction conditions.

**Species**	**Stoich. Matrix**	**Log K**	**Conc. [mol/l]**	**Log conc.**
4MePy (**3**)	0 0 1 0	0.00E+00	4.61E-03 (31%)	-2.34
4MePyH+ (**3H**$^+$)	0 0 1 1	5.98E+00	3.95E-03 (26%)	-2.40
DMAP (**5**)	0 1 0 0	0.00E+00	9.35E-06 (< 1%)	-5.03
DMAPH+ (**5H**$^+$)	0 1 0 1	9.12E+00	1.11E-02 (74%)	-1.96
H+(org)	0 0 0 1	0.00E+00	8.97E-07 (< 1%)	-6.05
PhBCat (**1**)	1 0 0 0	0.00E+00	4.62E-03 (31%)	-2.34
PhBCat4MePy (**1•3**)	1 0 1 0	2.48E+00	6.44E-03 (43%)	-2.19
PhBCatDMAP (**1•5**)	1 1 0 0	4.96E+00	3.94E-03 (26%)	-2.40
**Components**	**Mode**	**Initial Conc.**	**In or out of system**	
PhBCat (**1**)	total	1.50E-02	----	
DMAP (**5**)	total	1.50E-02	----	
4MePy (**3**)	total	1.50E-02	----	
H+(org)	total	1.50E-02	----	

Here follow the full time-resolved spectra of the experiment illustrated in Figure 5 (Manuscript)

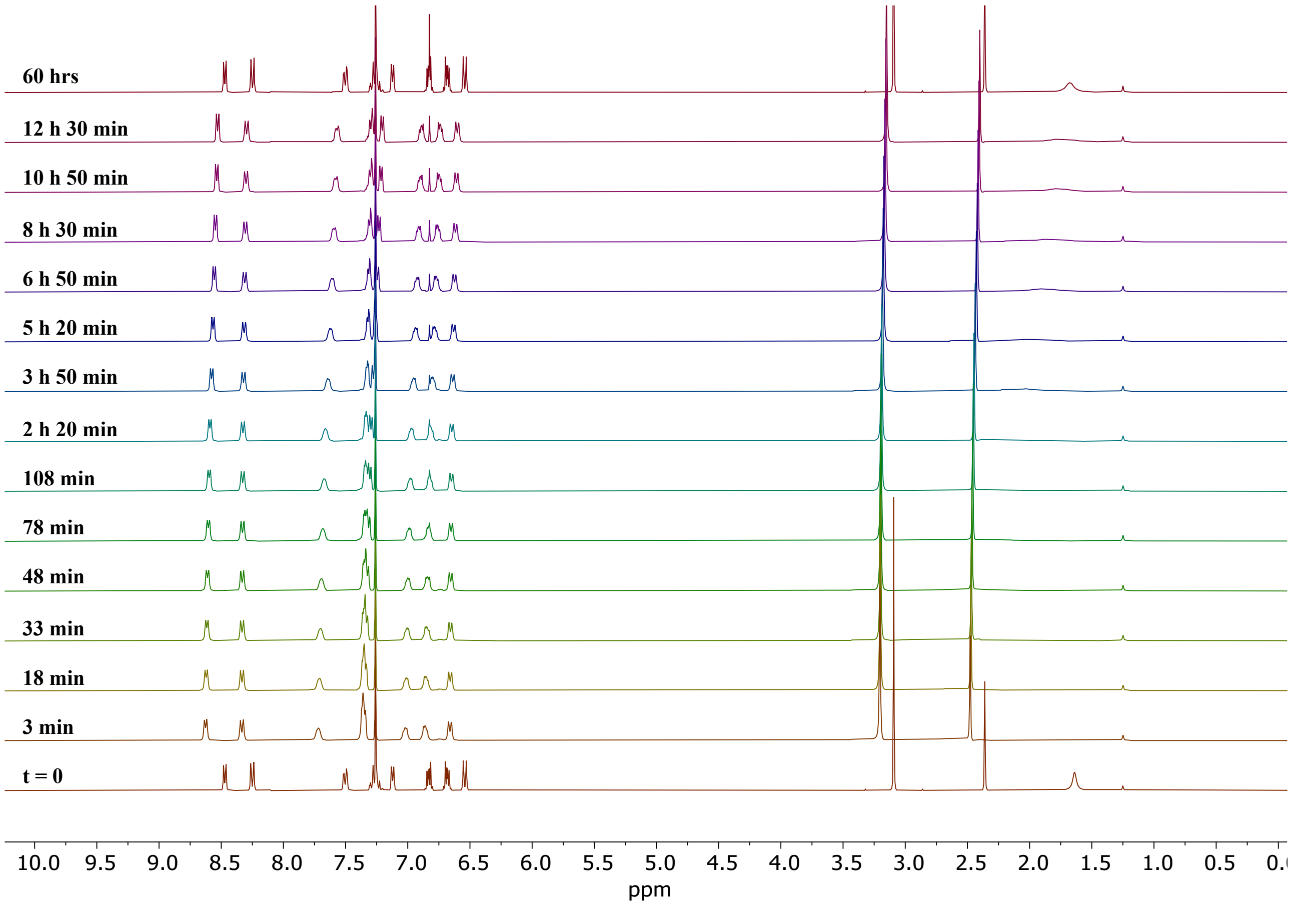

Figure S54. A DL composed of **5**, **1•3**, and related free building blocks driven by ACA **12**-CO_2H (see Main Text, where this Figure is reported as Figure 4). At t = 0, all compounds (**1**, **3**, **5** and **6**) are added in solution at 15.0 mM concentration ($CDCl_3$, 25 C). Full spectra.

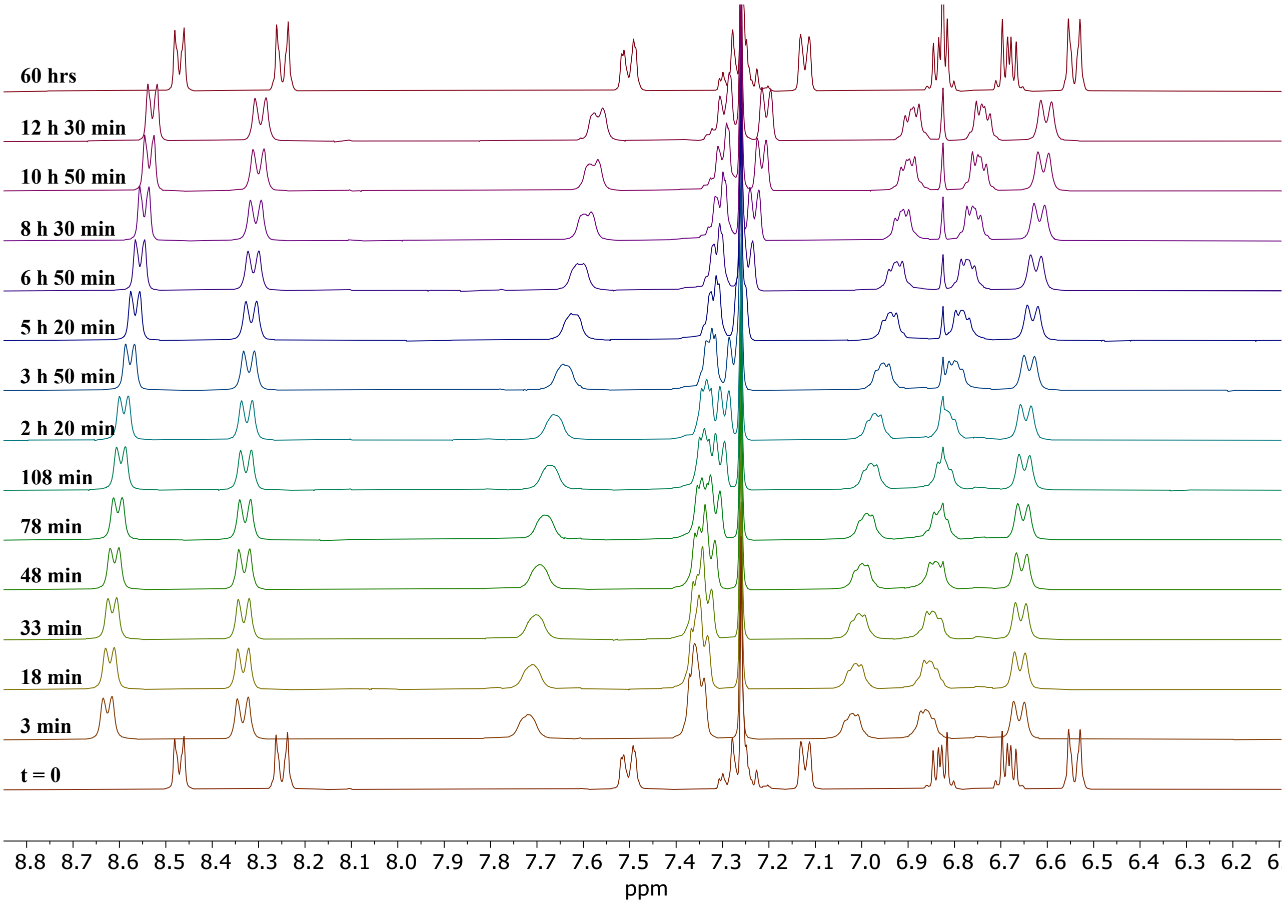

Figure S55. A DL composed of **5**, **1•3**, and related free building blocks driven by ACA **12**-CO_2H (see Main Text, where this Figure is reported as Figure 4). At t = 0, all compounds (**1**, **3**, **5** and **6**) are added in solution at 15.0 mM concentration ($CDCl_3$, 25 C). Zoom on the aromatics' region.

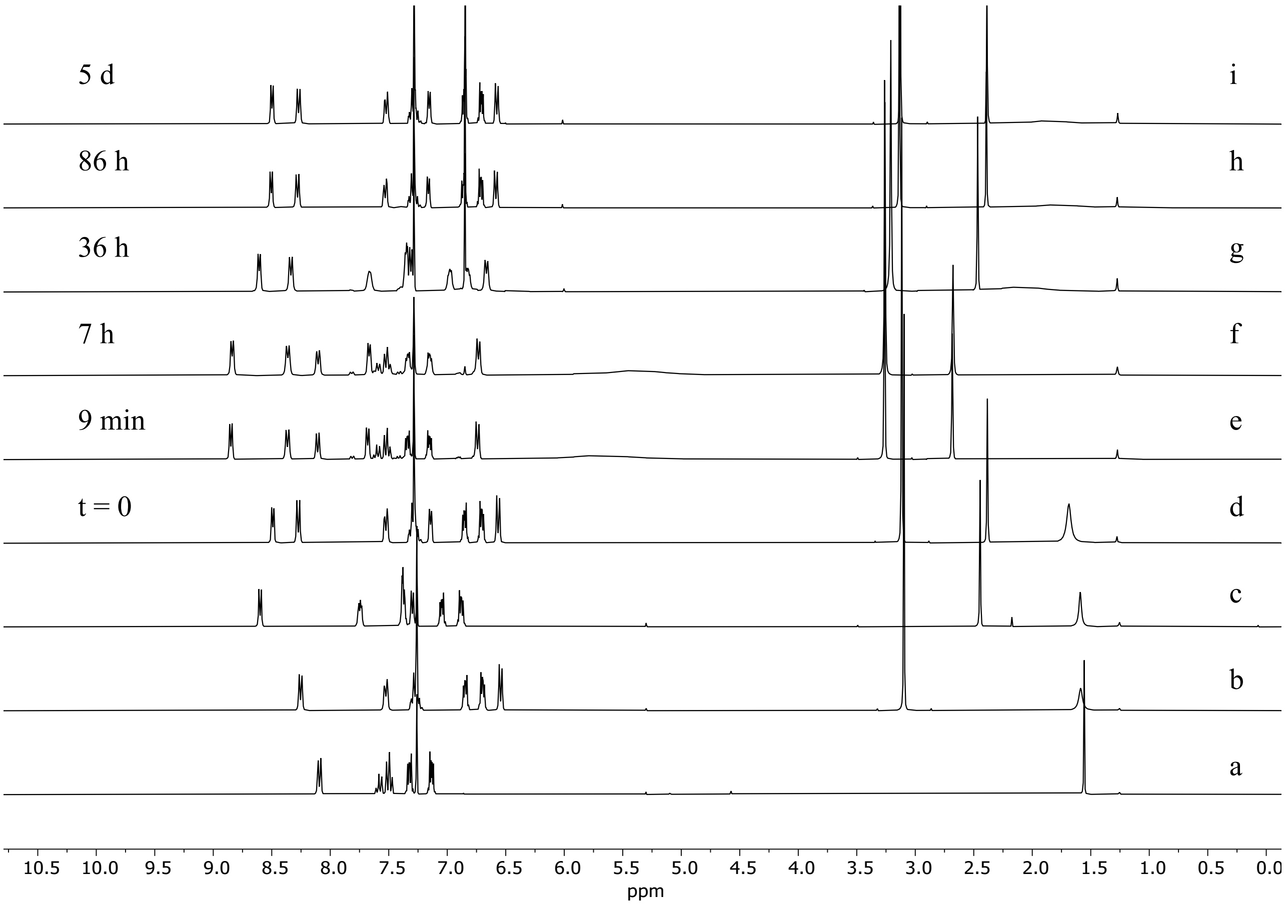

Figure S56. Behavior of a DL based on the $\equiv B^{-}{-}^{+}N\equiv$ bond under the action of excess ACA **12**-CO_2H. Traces a) 10 mM **1**; b) adduct **1•5** obtained by mixing 10 mM **1** with 10 mM **5**; c) adduct **1•3** obtained by mixing 10 mM **1** and 10 mM **3**; d) equilibrated solution of 10 mM **1**, **3** and **5** before addition of ACA **12**-CO_2H (t = 0); e-i) monitoring over time of solution in trace d after addition of ACA **12**-CO_2H. $CDCl_3$, 25 °C.

X-Ray Diffraction Analysis

The crystal structures of adducts **1•2** and **1•5** and of the cocrystals **1**•OH$^-$ + **9**•H$^+$ and **1**•OH$^-$ + **9**•H$^+$ were determined by X-ray diffraction analysis on single crystals. Crystal data and experimental details for data collection and structure refinement are reported in Table S23. Intensity data and cell parameters were recorded at 200(2) K on a Bruker D8 Venture PhotonII diffractometer (CuKα radiation λ = 1.54178 Å). The raw frame data were processed using SAINT and TWINABS/SADABS to yield the reflection data files.[16,17] The structures were solved by Direct Methods using the SHELXT program[18] and refined on F_o^2 by full-matrix least-squares procedures, using SHELXL-2019/3[19]. All non-hydrogen atoms were refined with anisotropic atomic displacements. The hydrogen atoms were included in the refinement at idealized geometry and refined "riding" on the corresponding parent atoms. In **1**•OH$^-$ + **9**•H$^+$**,** the H atoms of the ammonium cation and of the OH group were found in the difference Fourier map and refined freely. Adduct **1•2** was refined as a two-component twin using HKLF5-type data; therefore, no internal *R* value is reported. The BASF value refined to 0.44434(7). The weighting schemes used in the last cycle of refinement were $w = 1/ [\sigma^2 F_o^2 + (0.0484P)^2 + 6.555P]$ (**1•2**), $w = 1/ [\sigma^2 F_o^2 + (0.3652P)^2 + 5.9376P]$ (**1•5**) and $w = 1/ [\sigma^2 F_o^2 + (0.697P)^2 + 2.5248P]$ (**1**•OH$^-$ + **9**•H$^+$) where $P = (F_o^2 + 2F_c^2)/3$. The crystallographic data have been deposited with the Cambridge Crystallographic Data Centre as supplementary publication no. 2413767-2413768 (**1•2** and **1•5**) and 2492086 (**1**•OH$^-$ + **9**•H$^+$). They can be obtained free of charge on application to the CCDC, 12 Union Road, Cambridge, CB2 IEZ, UK (e-mail deposit@ccdc.cam.ac.uk or http://www.ccdc.cam.ac.uk).

Crystals of **1**•OH$^-$ + **8**•H$^+$ diffracted poorly, but it was nevertheless possible to determine the species which was formed and its bond connectivity (Figure S60). The main crystallographic data are as follows: $C_{17}H_{22}BNO_4$, Orthorhombic, *F*dd2, a = 30.389(2) Å, b = 34.164(2) Å, c = 6.577(4) Å, V = 6828.7(7) Å^3, Z = 16. Final R indexes [I>=2σ (I)]: R1 = 0.1448, wR2 = 0.4047.

Adduct **1•2** and **1•5** crystallize with 4 and 2.5 molecules in the asymmetric unit, respectively (see Figures S57 and S58).

Crystal data and structure refinement information for adducts 1·2 and 1·5, and for the cocrystal 1•OH⁻ + 9•H⁺

Table S23. Crystal data and structure refinement information for adducts **1•2** and **1•5** and for the cocrystal **1**•OH^- + **9**•H^+

Compound	**1•2**	**1•5**	**1**•OH^- + **9**•H^+
empirical formula	$C_{17}H_{14}BNO_2$	$C_{19}H_{19}BN_2O_2$	$C_{19}H_{26}BNO_4$
M	275.10	318.17	343.22
crys syst	Triclinic	Triclinic	Monoclinic
space group	*P*-1	*P*-1	*C*2/*c*
a/Å	9.3875(1)	10.3502(3)	17.1915(6)
b/Å	9.5728(2)	12.7006(3)	16.7124(6)
c/Å	31.7125(9)	16.1326(4)	12.9115(4)
α/°	89.935(2)	95.418(2)	-
β/°	89.950(2)	92.689(2)	103.272(1)
γ/°	81.370(1)	98.505(2)	-
V/Å^3	2817.56(10)	2084.00(9)	3610.5(2)
Z	8	5	8
ρ /g cm^{-3}	1.297	1.268	1.263
μ /mm^{-1}	0.670	0.652	0.699
F(000)	1152	840	1472
total reflections	11089	21188	20046
unique reflections (R_{int})	11089	5998 (0.0809)	3678 (0.0441)
observed reflections $[F_o>4\sigma(F_o)]$	10457	4629	3334
GOF on F^{2a}	1.026	1.089	1.046
R indices $[F_o>4\sigma(F_o)]^bR_1, wR_2$	0.0378, 0.961	0.0989, 0.2446	0.0505, 0.1348
largest diff. peak and hole (eÅ^{-3})	0.241, -0.210	0.489, -0.261	0.445, -0.383

[a]Goodness-of-fit S = $[\Sigma w(F_o^2-F_c^2)^2/(n-p)]1/2$, where n is the number of reflections and p the number of parameters. [b]$R_1 = \Sigma||F_o|-|F_c||/\Sigma|F_o|$, $wR_2 = [\Sigma[w(F_o^2-F_c^2)^2]/\Sigma[w(F_o^2)^2]]^{1/2}$.

Selected geometrical parameters (Å, °) for 1·2 and 1·5

Table S24. Selected geometrical parameters (Å, °) for **1•2** and **1•5**.

	**1•2**	**1•5**		**1•2**	**1•5**
B1A-O1A	1.473(3)	1.500(7)	B1C-O1C	1.478(3)	1.468(15)
B1A-O2A	1.475(2)	1.487(8)	B1C-O2C	1.463(2)	1.491(14)
B1A-N1A	1.664(3)	1.607(7)	B1C-N1C	1.655(3)	1.634(13)
B1A-C12A/C14A[a]	1.591(3)	1.585(8)	B1C-C12C/C14C[a]	1.600(3)	1.631(17)
O1A-B1A-N1A-C7A	66.1(2)	76.2(5)	O1C-B1C-N1C-C7C	29.4(2)	-69.0(9)
O1A-B1A-N1A-C11A	-110.9(2)	-93.5(5)	O1C-B1C-N1C-C11C	-152.7(2)	106.5(8)
B1B-O1B	1.466(2)	1.444(10)	B1D[b]-O1D	1.478(2)	1.424(12)
B1B-O2B	1.476(2)	1.475(9)	B1D[b]-O2D	1.474(3)	1.482(12)
B1B-N1B	1.659(2)	1.646(8)	B1D-N1D	1.650(3)	-
B1B-C12B/C14B[a]	1.606(3)	1.657(9)	B1D[b]-C12D/C14D[a]	1.597(3)	1.668(11)
O1B-B1B-N1B-C7B	-33.9(2)	-59.5(7)	O1D-B1D[b]-N1D[b]-C7D[b]	-179.5(2)	120.5(7)
O1B-B1B-N1B-C11B	148.2(2)	120.9(6)	O1D-B1D[b]-N1D[b]-C11D[b]	3.3(2)	-59.0(9)

[a]: for **1•5**

[b]: for **1•5** B1D is B1B, N1D is N1B, C7D is C7B and C11D is C11B

Table S25. Selected geometrical parameters (Å, °) for cocrystal **1**•OH^- + **9**•H^+

B1-O1	1.513(2)	N1-H1A···O3	165
B1-O2	1.529(2)	O1W-H1WA···O2	169
B1-O3	1.458(2)	O3-H3O·· O1W[i]	161
N1···O3	2.716(1)		
O1W···O2	2.738(1)		
O3···O1W[i]	2.775(1)		

i = -x+1,-y+1,-z+1

Ortep views

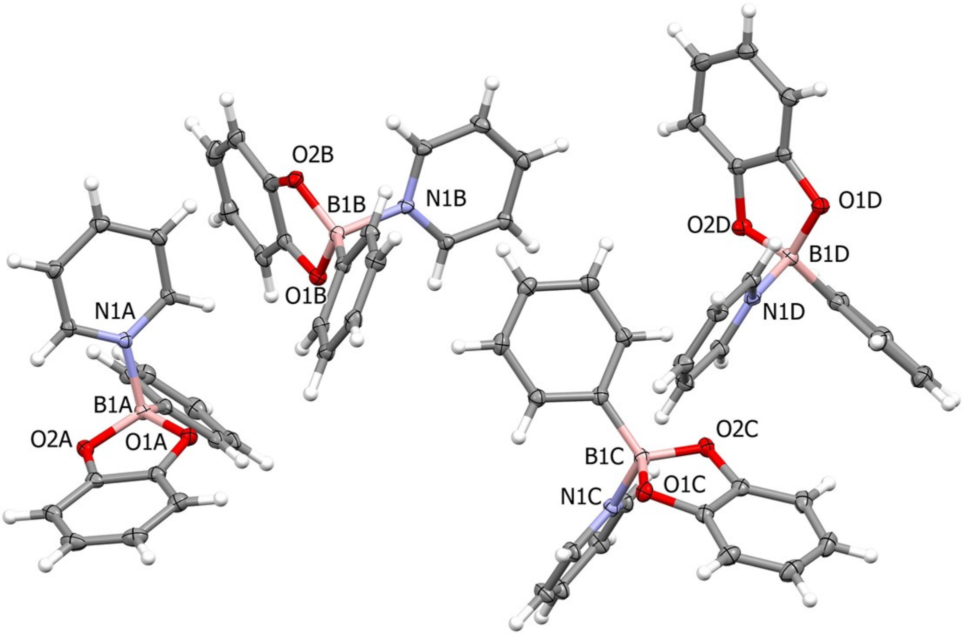

Figure S57. Ortep view of adduct **1•2** (four independent molecules labelled A, B, C and D) with partial labelling scheme.

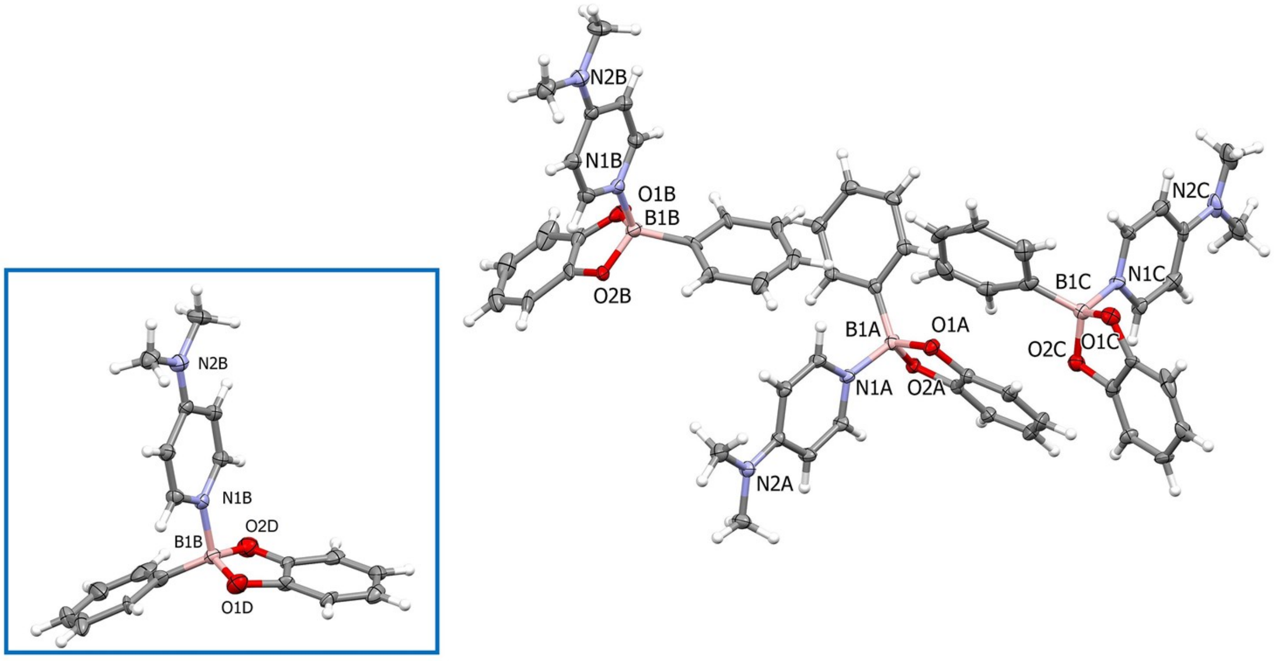

Figure S58. Ortep view of adduct **1•5** (three independent molecules labelled A, B and C) with partial labelling scheme. Molecule B is disordered over two positions (the second position labelled D is shown in the inset). Molecule C has an occupancy of 0.5.

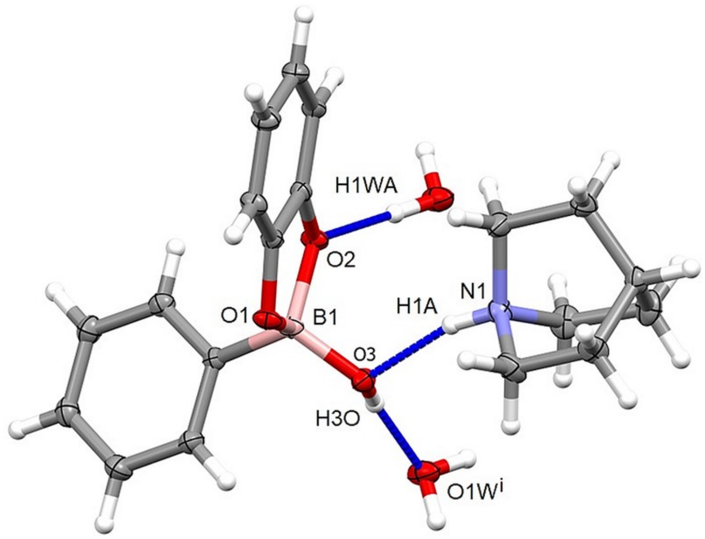

Figure S59. Ortep view of the cocrystal **1**•OH^{-} + **9**•H^{+} with partial labelling scheme. Only the major component of the ammonium cation is shown for clarity. H bonds are represented as blue dotted lines. Symmetry code i = -x+1, -y+1, -z+1.

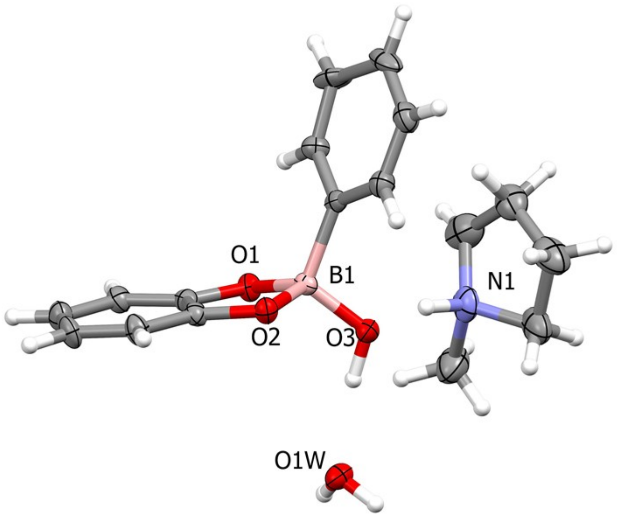

Figure S60. Ortep view of the cocrystal **1**•OH^{-} + **8**•H^{+} with partial labelling scheme.

List of B-N pyridine distances (Å) found in the CSD

Table S26. List of B–N pyridine distances (Å) found in the CSD for compounds similar to the adducts of the present study.

REFCODE	DIST	REFCODE	DIST	REFCODE	DIST	REFCODE	DIST
HIQDIY	1.619	DICSIS	1.586	EZAWAD	1.601	JUTMET	1.648
HIQDIY	1.63	DICSIS	1.62	EZAWAD	1.583	KEZTOB	1.652
HIQDIY	1.632	DICSIS	1.619	EZAWAD	1.575	KEZTUH	1.654
HIQDIY	1.634	DICSIS	1.603	EZAWEH	1.592	KEZVAP	1.663
AFISOY	1.641	DICSIS	1.602	EZAWEH	1.58	KEZVAP01	1.665
AFISUE	1.636	DICSOY	1.576	EZAWEH	1.559	KEZVET	1.664
AFISUE	1.655	DICSOY	1.609	EZAWEH	1.583	KEZVET	1.66
AFITAL	1.636	DICSOY	1.616	GAGCAV	1.655	KEZVIX	1.664
AFITAL	1.662	DICSOY	1.622	GAGCAV	1.645	KEZVUJ	1.646
ALEBIF	1.666	DICSOY	1.609	GAGCAV	1.646	KEZWAQ	1.631
ALEBOL	1.661	DICSOY	1.616	GAGCAV	1.651	KEZWAQ	1.635
ALEBOL	1.648	DICSOY	1.611	GAGCAV	1.658	KEZWEU	1.662
ALEBUR	1.628	DICSOY	1.614	GAGXIZ	1.623	KEZWIY	1.661
ALEBUR	1.652	DICSOY	1.602	GAGXIZ	1.664	KEZWOE	1.658
ALEBUR	1.691	DICSOY	1.608	GAGXOF	1.645	KEZWUK	1.655
AXIXUD	1.675	DICSUE	1.564	GAGXUL	1.654	KEZXAR	1.642
AXIXUD	1.653	DICSUE	1.597	GAGYAS	1.657	KEZXEV	1.666
AXIYEO	1.638	DICSUE	1.574	GENSAX	1.627	KEZXEV	1.671
AXIYEO	1.644	DICSUE	1.561	GENSAX	1.636	KOCQID	1.608
AXIYEO	1.643	DICSUE	1.601	GENSAX	1.639	MIQZUL	1.645
AXIYEO	1.651	DITKUN	1.674	GENSAX	1.623	MIQZUL	1.661
AXIYIS	1.649	DITKUN	1.68	GENSAX	1.625	MIRBAU	1.647
AXIYIS	1.669	DITLAU	1.702	GENSAX	1.627	NIXHIL	1.664
BEDFUP	1.661	DUDVEF	1.632	GENSEB	1.604	NOKBOG	1.694
BEXWUY	1.63	EDOLOA	1.676	GENSEB	1.641	ODAQOC	1.652
BEXWUY	1.62	EDOLOA	1.698	GENSEB	1.618	OWUMEA	1.642
BEXWU	1.624	EDOLOA	1.668	GENSEB	1.643	OWUME	1.639

Y						A	
BOQDAM	1.644	EDOLOA	1.673	GUQYEZ	1.645	OWUMEA	1.613
BOQDAM	1.66	EDOLUG	1.666	ISOWAQ	1.667	OWUMOK	1.701
BOQDEQ	1.651	EDOLUG	1.712	ISOWAQ	1.669	OWUMOK	1.627
BOQDEQ	1.654	EDOLUG	1.698	JIQKAZ	1.617	OWUMOK	1.659
CETGIV	1.682	EDOLUG	1.705	JIQKAZ	1.648	OWUMUQ	1.646
CETGOB	1.679	EDOLUG	1.635	JIQKAZ	1.648	OWUMUQ	1.667
CETGOB	1.684	EDOLUG	1.676	JIQKAZ	1.611	OWUMUQ	1.664
CETGUH	1.729	EDOMAN	1.645	JIQKAZ	1.604	OWUNAX	1.62
CETHAO	1.714	EDOMAN	1.647	JIQKAZ	1.612	OWUNAX	1.656
COYXIA	1.652	EDOMER	1.661	JIQKAZ	1.646	OWUNAX	1.656
COYXOG	1.654	EDOMER	1.696	JIQKAZ	1.61	OWUNAX	1.656
DEVCIU	1.665	EDOMIV	1.725	JIQKED	1.649	OWUNEB	1.687
DICSEO	1.59	EDOMIV	1.635	JIQKED	1.632	OWUNEB	1.682
DICSEO	1.603	EDOMIV	1.721	JIQKED	1.652	OWUNIF	1.646
DICSEO	1.624	EDOMOB	1.646	JIQKED	1.665	OWUNIF	1.674
DICSEO	1.593	EZAVUW	1.601	JUTLUI	1.646	PEBCAC	1.663
DICSEO	1.592	EZAWAD	1.589	JUTMAP	1.659	SAJFEQ	1.662
UWODUG	1.692	XUFYOP	1.68	ZAZZUZ	1.618	NOFZOB	1.71
UWOFAO	1.68	XUFYUV	1.802	ZAZZUZ	1.625	NOFZUH	1.652
UWOFES	1.697	XUQZIV	1.642	ZEBBAN	1.655	NOGBUK	1.657
UWOFIW	1.679	XUQZOB	1.643	ZEBBAN	1.662	NOGCAR	1.668
XANTAL	1.676	XUFYIJ	1.685	ZEBBER	1.645	YOQHAR	1.653
XANTAL01	1.67	XUFYOP	1.68	ZEBBER	1.656	YOQHAR	1.637
XANTAL02	1.663	XUFYUV	1.802	ZEBBER	1.625	FOYJAI	1.714
XANTEP	1.667	XUQZIV	1.642	ZEBBER	1.627	FOYJEM	1.682
XANTEP	1.65	XUQZOB	1.643	FOGTAA	1.659	FOYJUC	1.698
XAPHIJ	1.649	YIDRIO	1.66	FOGTAA	1.672	KEZVET01	1.668

XERDUV	1.664	YIXKUO	1.631	FOGTEE	1.667	POWMIB	1.649
XERDUV	1.68	YIXLAV	1.64	FOGTEE	1.661	POWMIB	1.636
XERFAD	1.66	YIXLEZ	1.648	FOGTII	1.675	POWMIB	1.646
XOZJUU	1.65	YOJBAC	1.637	FOGTII	1.658	POXBUD	1.657
XUFYAB	1.677	YONZIN	1.678	FOGYEJ	1.664	POXCAK	1.646
XUFYEF	1.674	YONZIN	1.691	FOGYEJ	1.66	POXCIS	1.671
XUFYIJ	1.685	ZALFIF	1.633	HOLNUV	1.698		

Computational Study

The initial structures of **1**, the *N*ArHets, and the **1**·*N*ArHet adducts were optimized using the following parameters in the ORCA_6.1.0 input file:

```
! r2SCAN-3c DEFGRID2 TightSCF CPCM OPT FREQ
%scf MaxIter 500
end
%geom
MaxIter 500
end
%cpcm
smd true
SMDsolvent "chloroform"
end


charge = 0, multiplicity = 1
```

List of optimized geometries in Cartesian coordinates

Phenylboronic acid catechol ester 1

```
xyz
   C       0.97509       0.45948      -0.82641
   C      -0.40405       0.69039      -0.85937
   C      -1.28602      -0.25545      -0.32924
   C      -0.79036      -1.43392       0.23559
   C       0.58797      -1.66922       0.27098
   C       1.47842      -0.72291      -0.26084
   H       1.64380       1.20339      -1.24279
   H      -0.78910       1.60256      -1.29663
   H      -2.35294      -0.07540      -0.35632
   H      -1.47436      -2.16574       0.64559
   H       0.95419      -2.58867       0.71193
   B       3.02586      -0.98675      -0.22659
   O       4.02360      -0.08924      -0.75494
   O       3.64514      -2.16831       0.32152
   C       4.95178      -1.95051       0.10882
   C       5.17176      -0.74105      -0.51756
   C       6.45860      -0.31140      -0.83774
   C       7.53864      -1.14743      -0.50475
   C       7.31357      -2.38415       0.13599
   C       6.00631      -2.79584       0.44929
   H       6.62130       0.63863      -1.32966
   H       8.54897      -0.84041      -0.74187
   H       8.15218      -3.02025       0.38755
   H       5.82378      -3.74282       0.94011
```

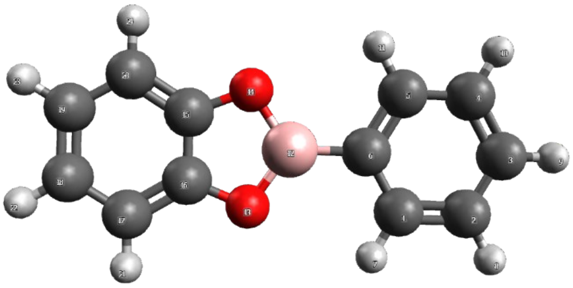

Pyridine 2

xyz

C	-1.01336	1.47854	0.00000
C	-1.97301	0.47213	0.00000
C	-1.54809	-0.85267	0.00000
N	-0.25826	-1.21377	0.00000
C	0.65571	-0.23443	0.00000
C	0.32964	1.11813	0.00000
H	-1.30700	2.52469	0.00000
H	-3.03371	0.70528	0.00000
H	-2.27719	-1.66235	0.00000
H	1.69947	-0.54691	0.00000
H	1.11423	1.86906	0.00000

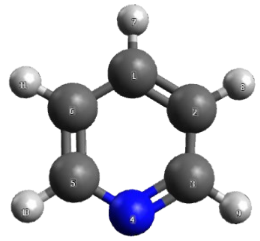

4-methylpyridine 3

xyz

C	-1.00242	1.45587	-0.00019
C	-1.95293	0.43053	-0.00068
C	-1.53243	-0.89199	-0.00046
N	-0.24336	-1.26239	0.00016
C	0.66518	-0.27957	0.00059
C	0.33872	1.07262	0.00043
C	-1.41657	2.89670	-0.00004
H	-0.54961	3.56241	-0.00330
H	-2.02515	3.12454	0.88263
H	-2.03109	3.12325	-0.87887
H	-3.01549	0.66210	-0.00123
H	-2.26663	-1.69724	-0.00085
H	1.71084	-0.58663	0.00105
H	1.12756	1.82052	0.00075

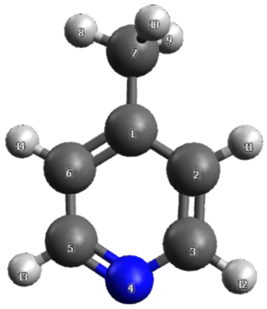

1-methylimidazole 4

xyz

N	1.35015	1.32028	2.95761
C	0.36115	0.97867	3.76541
N	0.50555	1.52651	5.00193
C	1.66514	2.26700	4.98124
C	2.17114	2.12772	3.71348
H	-0.48127	0.34419	3.51816
H	2.01028	2.80930	5.85036
H	3.07550	2.56237	3.30757
C	-0.39135	1.36431	6.13800
H	-1.22270	0.71993	5.84393
H	-0.78762	2.33504	6.45242
H	0.13722	0.90267	6.97801

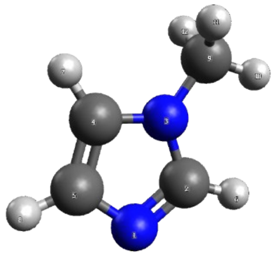

4-dimethylaminopyridine 5

```
xyz
   C       -0.23556        1.21484        0.06578
   C       -1.44854        0.48633        0.05492
   C       -1.41281       -0.89412       -0.02916
   N       -0.29367       -1.63143       -0.11378
   C        0.85436       -0.93479       -0.11862
   C        0.94594        0.44334       -0.03966
   H        1.92597        0.90499       -0.06117
   H       -2.41000        0.98301        0.10968
   H       -2.35363       -1.44516       -0.03367
   H        1.77167       -1.51906       -0.19584
   N       -0.20663        2.57341        0.17619
   C       -1.44847        3.33278        0.09580
   H       -1.93566        3.23305       -0.88654
   H       -1.23107        4.38856        0.26629
   H       -2.15783        3.01127        0.86701
   C        1.05109        3.28729       -0.00826
   H        1.80592        2.94941        0.71123
   H        0.88397        4.35203        0.16315
   H        1.45774        3.15953       -1.02333
```

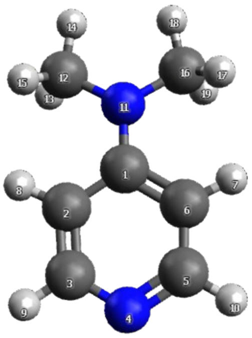

4-morpholinopyridine 6

```
xyz
   O       -1.68362        2.77996       -0.38765
   C       -2.82244        2.13763        0.18154
   C       -2.92005        0.67891       -0.23147
   H       -3.70649        2.67596       -0.17445
   H       -2.78028        2.21414        1.28149
   N       -1.68721       -0.04330        0.10337
   H       -3.13147        0.60713       -1.31276
   H       -3.76443        0.24790        0.31331
   C       -0.47814        0.65895       -0.35041
   C       -0.50596        2.11767        0.06926
   H       -0.38695        0.58693       -1.44819
   H        0.41228        0.21110        0.09572
   H        0.34825        2.63981       -0.37320
   H       -0.43838        2.19514        1.16788
   C       -1.70107       -1.42364        0.09540
   C       -2.89986       -2.16793        0.03452
   C       -2.85407       -3.55127        0.08830
   N       -1.73249       -4.27909        0.18554
   C       -0.59346       -3.57066        0.22644
   C       -0.51696       -2.18978        0.17941
   H        0.46321       -1.73178        0.22204
   H       -3.86792       -1.69304       -0.06233
   H       -3.78945       -4.10921        0.04060
   H        0.33011       -4.14515        0.29906
```

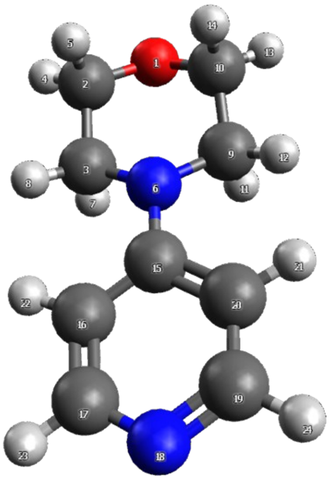

Adduct 1•2

xyz

C	-1.86625	5.17333	-0.82325
C	-2.08211	4.11514	-1.70171
C	-1.52823	2.88311	-1.41475
N	-0.78466	2.68754	-0.30967
C	-0.56812	3.70156	0.54412
C	-1.09894	4.96020	0.31435
H	-2.29313	6.15122	-1.02512
H	-2.67566	4.23749	-2.60097
H	-1.66587	2.01984	-2.05937
H	0.03924	3.47192	1.41339
H	-0.90594	5.75649	1.02482
C	-2.40260	0.37616	0.96746
C	-3.42323	-0.55475	1.14244
C	-3.40464	-1.75067	0.42634
C	-2.35934	-2.00676	-0.45762
C	-1.33820	-1.07181	-0.62145
C	-1.33833	0.13758	0.08519
H	-2.43747	1.30838	1.53079
H	-4.23557	-0.34854	1.83504
H	-4.20175	-2.47822	0.55693
H	-2.33907	-2.93720	-1.01985
H	-0.52421	-1.28403	-1.31141
B	-0.13878	1.18043	-0.07039
O	0.74920	0.94223	-1.23041
C	2.00962	1.19821	-0.78814
C	2.01466	1.39803	0.60067
O	0.75382	1.28615	1.10454
C	3.17752	1.24537	-1.52588
C	4.37185	1.50018	-0.83244
C	4.37660	1.69634	0.54590
C	3.18659	1.64548	1.29002
H	3.18237	1.79324	2.36628
H	3.16698	1.08718	-2.60056
H	5.30592	1.54429	-1.38543
H	5.31414	1.89175	1.05869

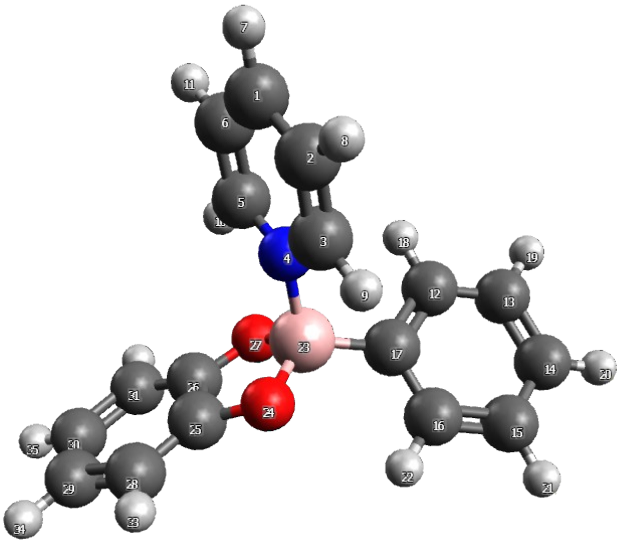

Adduct 1•3

xyz

C	-1.93156	5.21166	-0.68014
C	-2.15711	4.15116	-1.56760
C	-1.59578	2.91823	-1.32335
N	-0.82131	2.69445	-0.24257
C	-0.58911	3.69534	0.62142
C	-1.12841	4.95509	0.43044
C	-2.53476	6.55709	-0.92499
H	-2.25787	7.27003	-0.14525
H	-3.62773	6.48377	-0.96269
H	-2.20872	6.95126	-1.89431
H	-2.77445	4.28762	-2.45028
H	-1.75128	2.07120	-1.98532
H	0.03998	3.45547	1.47237
H	-0.91571	5.73476	1.15491
C	-2.43349	0.35218	0.94458
C	-3.43639	-0.59972	1.10991
C	-3.37815	-1.80142	0.40589
C	-2.31133	-2.04186	-0.45670
C	-1.30863	-1.08568	-0.61081
C	-1.34820	0.13009	0.08404
H	-2.49959	1.28855	1.49803
H	-4.26580	-0.40541	1.78557
H	-4.16111	-2.54548	0.52885
H	-2.26008	-2.97654	-1.00987
H	-0.47774	-1.28553	-1.28421
B	-0.16419	1.19431	-0.05656
O	0.71455	0.99200	-1.23306
C	1.97824	1.23828	-0.79708
C	1.99816	1.39786	0.59717
O	0.74391	1.26957	1.11155
C	3.13823	1.31003	-1.54552
C	4.33980	1.54730	-0.85814
C	4.35936	1.70264	0.52518
C	3.17747	1.62728	1.28035
H	3.18483	1.74335	2.36049
H	3.11622	1.18337	-2.62422
H	5.26763	1.60986	-1.41982
H	5.30206	1.88503	1.03333

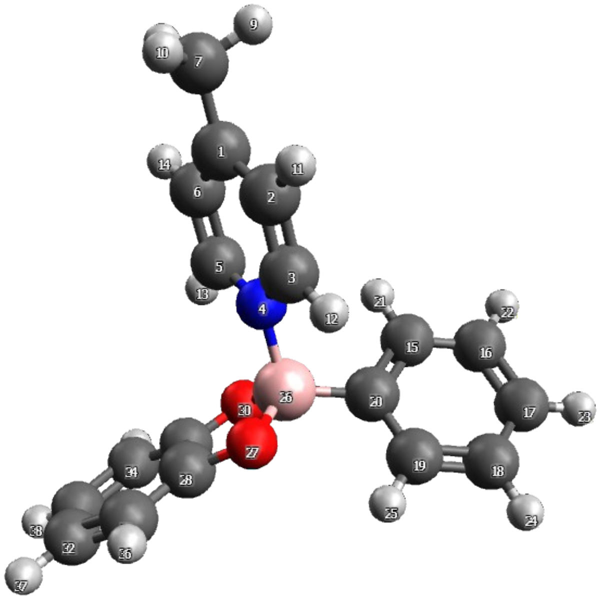

Adduct 1•4

xyz

C	-0.98269	0.56353	1.09257
C	-2.13502	-0.08807	0.66054
C	-2.03557	-1.30646	-0.00995
C	-0.77955	-1.85972	-0.24589
C	0.36877	-1.19534	0.18382
C	0.29349	0.02819	0.86137
B	1.61186	0.81790	1.30651
O	1.86101	2.08312	0.55634
C	3.18483	2.08376	0.25077
C	3.78184	0.86768	0.62264
O	2.86605	0.02944	1.16942
C	3.91518	3.08853	-0.35648
C	5.28186	2.85338	-0.58320
C	5.87467	1.65022	-0.21145
C	5.12637	0.63155	0.40205
H	5.58176	-0.31189	0.69013
H	3.44413	4.02325	-0.64813
H	5.88106	3.62577	-1.05727
H	6.93316	1.49120	-0.39767
H	1.34494	-1.63426	-0.01136
H	-1.07966	1.51157	1.62092
H	-3.11216	0.35067	0.84785
H	-2.93257	-1.82094	-0.34553
H	-0.69418	-2.81039	-0.76681
N	1.48500	1.24828	2.85607
C	1.15509	0.41555	3.90334
C	1.25791	1.12919	5.05978
N	1.65423	2.40145	4.70122
C	1.78485	2.44142	3.36654
C	1.88126	3.51495	5.61910
H	2.65773	3.24493	6.33991
H	0.95502	3.75248	6.14959
H	2.20548	4.38510	5.04600
H	2.08329	3.31198	2.79997
H	0.87191	-0.61448	3.74297
H	1.08469	0.85477	6.08967

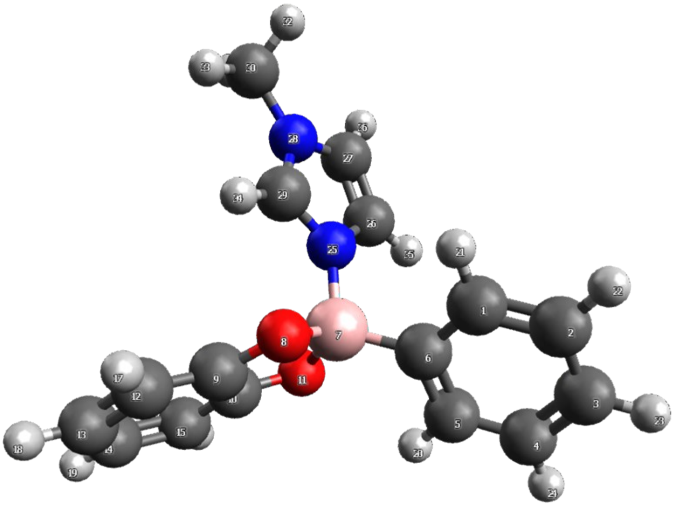

Adduct 1•5

xyz

C	0.70544	1.37064	1.55492
C	-0.52121	1.53272	0.91526
C	-1.10414	0.45828	0.24532
C	-0.44893	-0.77059	0.21784
C	0.78242	-0.92023	0.85433
C	1.38606	0.14409	1.53663
H	1.14125	2.21948	2.08119
H	-1.02603	2.49549	0.94106
H	-2.06385	0.57841	-0.25105
H	-0.89831	-1.61383	-0.30122
H	1.28717	-1.88366	0.82276
B	2.82707	-0.02008	2.21562
O	3.93869	0.59959	1.44625
O	3.23787	-1.43690	2.42305
C	4.54670	-1.50003	2.07145
C	4.96339	-0.29307	1.48639
C	6.25349	-0.12654	1.01843
C	7.13878	-1.20916	1.15379
C	6.72702	-2.40509	1.73484
C	5.41349	-2.56925	2.20520
H	6.56581	0.80855	0.56175
H	8.16000	-1.10656	0.79751
H	7.42978	-3.22841	1.82801
H	5.08437	-3.50158	2.65558
C	3.64929	2.25020	5.27732
C	3.64572	1.68649	4.02665
N	2.81551	0.67954	3.68194
C	1.95653	0.21219	4.61636
C	1.89611	0.71293	5.88849
C	2.75858	1.77475	6.27451
H	4.34664	3.05407	5.47427
H	4.31840	2.03258	3.24883
H	1.18111	0.28117	6.57677
H	1.30244	-0.59609	4.30120
N	2.72976	2.29904	7.51541
C	1.81511	1.76489	8.52185
H	0.76923	1.87050	8.20675
H	1.94723	2.31840	9.45167
H	2.01574	0.70441	8.72200
C	3.63733	3.38500	7.87738
H	4.68712	3.07328	7.79877
H	3.44480	3.68023	8.90883
H	3.48383	4.26220	7.23627

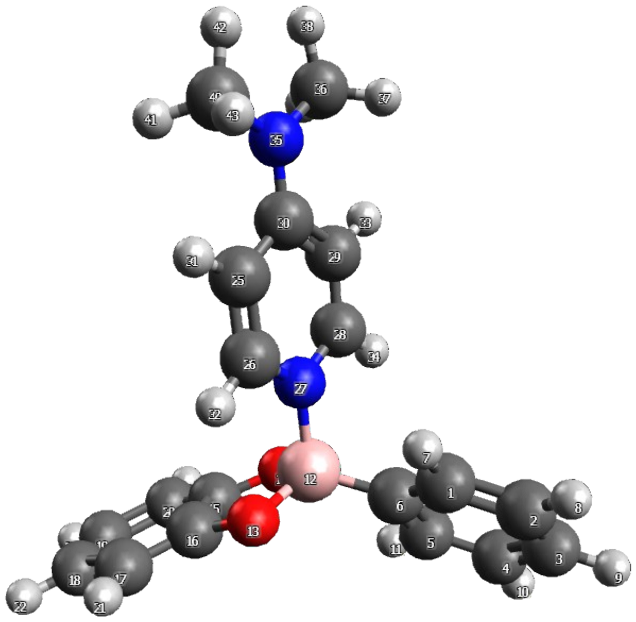

Adduct 1•6

xyz

C	-1.78798	5.38130	-1.13046
C	-2.11904	4.20972	-1.86243
C	-1.61655	2.99281	-1.48547
N	-0.78578	2.83378	-0.43239
C	-0.43746	3.93273	0.26626
C	-0.89881	5.18795	-0.04312
H	-2.76625	4.23249	-2.72817
H	-1.86980	2.09045	-2.03513
C	-2.46809	0.65467	0.97163
C	-3.49283	-0.25265	1.22902
C	-3.47681	-1.50984	0.62667
C	-2.42932	-1.84926	-0.22628
C	-1.40400	-0.93680	-0.47130
C	-1.40065	0.33328	0.11985
H	-2.50105	1.63550	1.44535
H	-4.30649	0.01959	1.89699
H	-4.27683	-2.21977	0.82120
H	-2.41097	-2.82742	-0.70093
H	-0.58833	-1.21457	-1.13564
B	-0.19019	1.35345	-0.11766
O	0.68619	0.99584	-1.26747
C	1.95948	1.19241	-0.84239
C	1.98601	1.48160	0.53167
O	0.72681	1.49158	1.04436
C	3.12576	1.11080	-1.58098
C	4.33931	1.32624	-0.90687
C	4.36483	1.61105	0.45542
C	3.17690	1.69215	1.20131
H	3.18838	1.90980	2.26570
H	3.09906	0.88359	-2.64306
H	5.27129	1.26836	-1.46227
H	5.31614	1.77368	0.95425
C	-3.87562	8.03830	-2.68646
O	-3.18845	9.20477	-2.24188
C	-2.71140	8.97773	-0.91857
C	-1.72509	7.82355	-0.86097
H	-3.56234	8.78068	-0.24481
H	-2.21004	9.89438	-0.59304
N	-2.30360	6.60231	-1.44183
H	-1.47905	7.66263	0.19039
H	-0.80100	8.08906	-1.39979
C	-2.96100	6.82646	-2.73842
H	-4.25117	8.24739	-3.69286
H	-4.73344	7.83600	-2.02305
H	-3.57978	5.96711	-3.00332
H	-2.19815	6.96313	-3.52209
H	-0.55106	6.00653	0.57141
H	0.23943	3.77270	1.09900

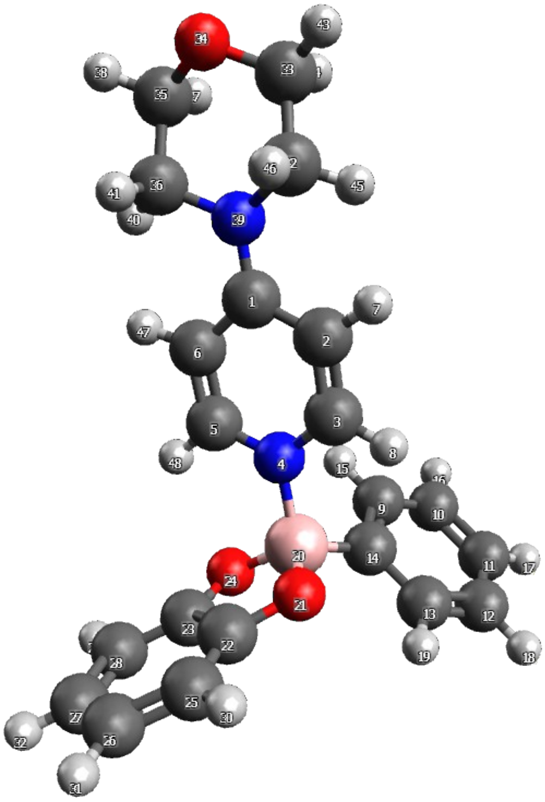

Energy calculation, computed B-N bond distances, and Hirshfeld partial charges

The optimized structures were processed with the following parameters:

```
! r2SCAN-3c def2-QZVPP RIJCOSX AutoAux DEFGRID3 VeryTightSCF CPCM SP FREQ
%scf MaxIter 500
end
%cpcm
smd true
SMDsolvent "chloroform"
end
```

From the output files, Gibbs free energy values (298.15 K, 1 atm) were extracted. These values correspond to the acceptor energy (E_a, the acceptor is **1**), donor energy (E_d, the *N*ArHets), and the adduct energy (E_{adduct}, which is the **1**-*N*ArHets). The stabilization energies of **1**-*N*ArHet were calculated using the formula $E_{stab} = E_{adduct} – (E_a + E_d)$ and the resulting values in Hartree were converted in kcal/mol. These stabilization energies were then compared with the experimental one derived from K_{bind} values (see Table below).

Table S27. List of calculated energies, and experimental energies (last column).

**1**·*N*ArHet adduct	Adduct energy E_{adduct} [Eh]	**1** acceptor energy E_a [Eh]	Donor energy E_d [Eh]	$E_{stab} = E_{adduct} – (E_a + E_d)$ [Eh]	E_{stab} [kcal/mol]	Experimental [kcal/mol]
**1•2**	-886.1082484	-637.9065415	-248.1986208	-0.00308605	-1.936505633	-2.49873627
**1•3**	-925.3970432	-637.9065415	-287.4860104	-0.00449128	-2.818291674	-3.377704022
**1•4**	-903.3505533	-637.9065415	-265.4373496	-0.0066622	-4.180550487	-4.74126425
**1•5**	-1020.003828	-637.9065415	-382.0875014	-0.00978524	-6.140267456	-6.755408044
**1•6**	-1172.590849	-637.9065415	-534.6763742	-0.00793348	-4.978282501	-5.275930484

Table S28. Computed B-N distances and Hirshfeld partial atomic charges.[20]

Compound	B-N bond length [Å]	B Hirshfeld partial atomic charge	N Hirshfeld partial atomic charges	H_{ortho1} Hirshfeld partial atomic charges	H_{ortho2} Hirshfeld partial atomic charges	$H_{ortho1,2}$ average
**1**	-	0.22902	-	0.047971	0.047925	0.047948
**1•2**	1.657	0.136059	-0.027941	0.033557	0.03238	0.0329685
**1•3**	1.648	0.135094	-0.03401	0.032268	0.032841	0.0325545
**1•4**	1.613	0.133732	-0.061619	0.030664	0.03215	0.031407
**1•5**	1.625	0.132361	-0.057639	0.031643	0.031025	0.031334
**1•6**	1.626	0.133105	-0.054269	0.031007	0.032353	0.03168

Correlation between Mayr's nucleophilicity parameters and $logK_{bind}$ (or log_{Kobs}) values

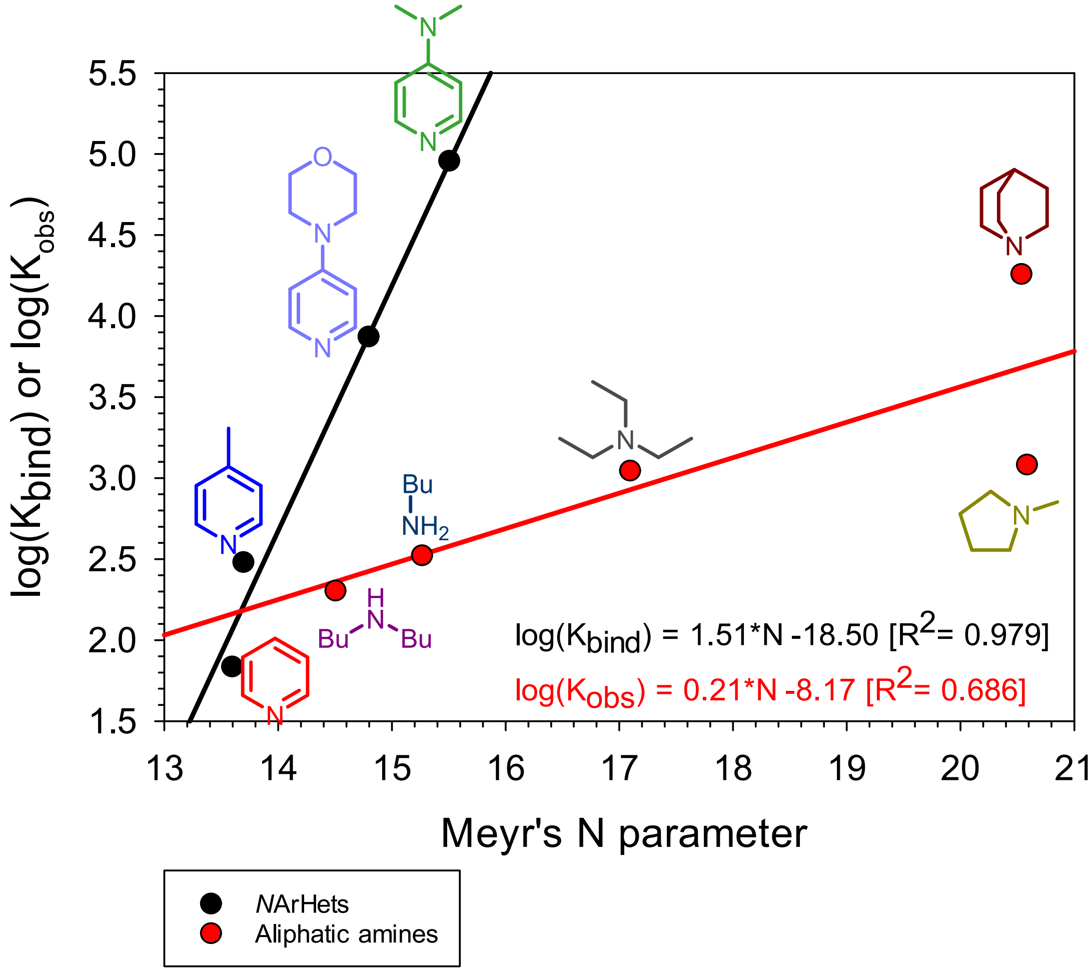

Figure S61. Correlation between the base-10 logarithm of the association constant (K_{bind} for *N*ArHets and K_{obs} for aliphatic amines) and Mayr's N parameter.

Figure S61 shows the correlation between the measured $logK_{obs}$ values (for *N*ArHets, or log K_{obs} for aliphatic amines) and Mayr's nucleophilicity parameter N. It is apparent that the log K_{bind} values are strongly sensitive to the N parameter of the *N*ArHets. The points related to aliphatic amines behave differently.

Table S29 contains all the values used to generate the graphs in Figure S61.

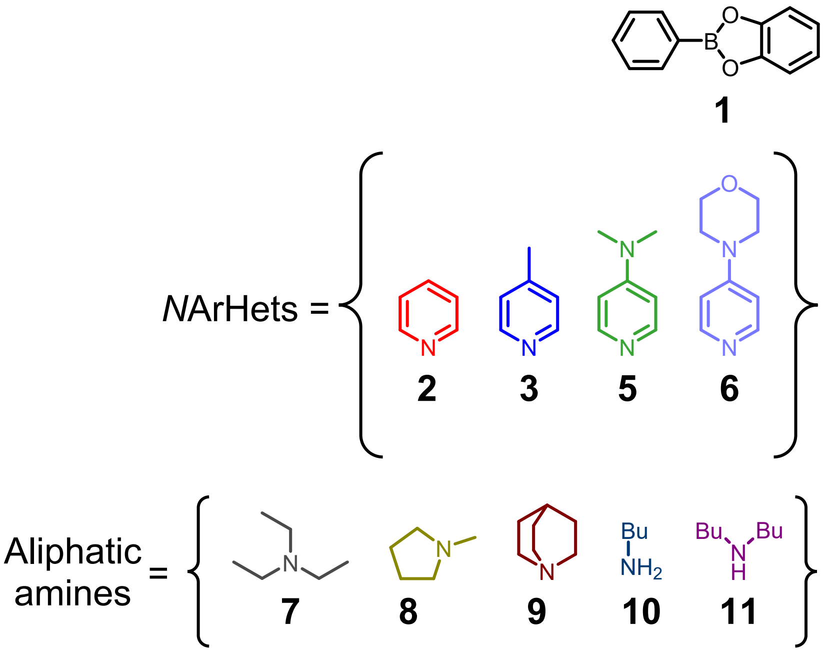

Figure S62. Structure of the *N*ArHets and aliphatic amines considered for the $logK_{bind}$ (or $logK_{obs}$)-N correlation.

Table S29. $log(K_{bind})$ or $log(K_{obs})$ and Mayr's N values for the compounds listed in Figure S62.

Compound	$logK_{bind}$ (or $logK_{obs}$) with **1**	Mayr's N Value	DOI (ref.)	Notes
**2**	1.83	13.6	10.1055/s-0036-1590504(ref.21)	
**3**	2.48	13.7	10.1002/chem.200600941 (ref.22)	in CH_2Cl_2
**5**	4.95	15.51	10.1021/jp3049247 (ref.23)	
**6**	3.87	14.8	10.1021/jp3049247 (ref. 23)	
**7**	3.04	17.1	10.1002/poc.1707 (ref.24)	
**8**	3.08	20.59	10.1002/poc.1707 (ref. 24)	
**9**	4.26	20.54	10.1002/anie.200701489 (ref.25)	
**10**	2.52	15.27	10.1002/ejoc.200900925 (ref.26)	
**11**	2.30	14.51	10.1002/ejoc.200900925 (ref. 26)	N value for diisoprolyamine

Table S29 reports the experimental data employed to generate the graphs in Figure S61. For each Mayr's N value, the original reference is reported. Unless otherwise stated, all N values are measured in acetonitrile. For compound **3**, the N value in acetonitrile was not reported in the literature; the one in dichloromethane was used in its place. It has to be noted that, when available- the N values of pyridines in acetonitrile and dichloromethane do not differ significantly. The N value of diisopropylamine was used in place of that of dibutylamine **11**.

All N values are taken from "Mayr's Database Of Reactivity Parameters". [27]

References

1 Z. Wu, R. Vlaming, M. Donohoe and D. A. Pratt, *J. Am. Chem. Soc.,* 2024, **146**, 1153–1166.
2 pK_aH is the pK_a of the conjugated acid to a given base.
3 A. Kütt, S. Selberg, I. Kaljurand, S. Tshepelevitsh, A. Heering, A. Darnell, K. Kaupmees, M. Piirsalu and I. Leito, *Tetrahedron Lett.*, 2018, **59**, 3738–3748.
4 J.-C. Hallé, J. Lelievre and F. Terrier, *Can. J. Chem.*, 1996, **74**, 613–620.
5 S. P. Anthony, S. Varughese and S. M. Draper, *J. Phys. Org. Chem.*, 2010, **23**, 1074–1079.
6 S. Kaiho, A. A. R. Hmayed, K. R. Delle Chiaie, J. C. Worch and A. P. Dove, *Macromolecules*, 2022, **55**, 10628–10639.
7 N. Bedeković, V. Stilinović, T. Friščić and D. Cinčić, *New J Chem*, 2018, **42**, 10584–10591.
8 V. I. Rybachenko; K. Y. Chotii; V. V. Kovalenko; G. Shreder *Russ. J. Phys. Chem*, 2003, **77**, 1695-1698
9 V. Frenna, N. Vivona, G. Consiglio and D. Spinelli, *J Chem Soc Perkin Trans 2*, 1985, 1865–1868.
10 R. Adams, M. Carmack and J. E. Mahan, *J. Am. Chem. Soc.*, 1942, **64**, 2593–2597.
11 J. Hine and Y. J. Chen, *J. Org. Chem.*, 1987, **52**, 2091–2094.
12 A. Gervasini and A. Auroux, *J. Phys. Chem.*, 1993, **97**, 2628–2639.
13 D. Yang, G. Zuccarello and B. R. Mattes, *Macromolecules*, 2002, **35**, 5304–5313.
14 E. Chrystiuk and A. Williams, *J. Am. Chem. Soc.*, 1987, **109**, 3040–3046.
15 Müller B., ChemEQL V3.2. (https://www.eawag.ch/en/department/surf/projects/chemeql)
16 G. M. Sheldrick, *TWINABS* 2012/1. Bruker, Madison, Wisconsin, USA, 2012.
17 L. Krause, R. Herbst-Irmer, G. M. Sheldrick and D. Stalke, *J. Appl. Crystallogr.*, 2015, **48**, 3–10.
18 G. M. Sheldrick, *Acta Cryst*., 2015, ***A71***, 3-8.
19 G. M. Sheldrick, *Acta Cryst*., 2008, ***A64***, 112-122.
20 F. L. Hirshfeld, *Theoret. Chim. Acta*, 1977, **44**, 129–138.
21 G. Berionni, E. Follet, H. Zipse, S. Lakhdar and A. Ofial, *Synthesis (Mass.)*, 2017, **49**, 3495–3504.
22 F. Brotzel, B. Kempf, T. Singer, H. Zipse and H. Mayr, *Chemistry*, 2007, **13**, 336–345.
23 T. A. Nigst, J. Ammer and H. Mayr, *J. Phys. Chem. A*, 2012, **116**, 8494–8499.
24 J. Ammer, M. Baidya, S. Kobayashi and H. Mayr, *J. Phys. Org. Chem.*, 2010, **23**, 1029–1035.
25 M. Baidya, S. Kobayashi, F. Brotzel, U. Schmidhammer, E. Riedle and H. Mayr, *Angew. Chem. Int. Ed Engl.*, 2007, **46**, 6176–6179.
26 T. Kanzian, T. A. Nigst, A. Maier, S. Pichl and H. Mayr, *European J. Org. Chem.*, 2009, **2009**, 6379–6385.
27 Mayr, H. Ofial, A. R., https://www.cup.uni-muenchen.de/oc/mayr/DBintro.html
